# Supplementary material for: The Role of Ferroptosis and Cuproptosis in Curcumin against Hepatocellular Carcinoma
Source: Molecules. 2023 Feb 8;28(4):1623. doi: 10.3390/molecules28041623 (PMC9964324; doi:10.3390/molecules28041623)
Supplement: Supplementary file 1 [file molecules-28-01623-s001.zip › Table S1.pdf]

Table S1

gene	PLC logFC	adj.P.Val	gene	KMCH logFC	adj.P.Val	gene	Huh7 logFC	adj.P.Val
ALPL	-3.52065	1.51E-05	FGA	-5.02232	3.05E-06	FADS2	-4.02891	1.86E-05
CHAC1	-3.20102	3.71E-05	HMGCS2	-4.92902	1.94E-06	SOX21	-3.45296	0.000187
SLC3A2	-2.92705	0.000107	GC	-4.50093	1.79E-06	HAMP	-3.36228	2.42E-05
F2RL2	-2.8482	1.49E-05	FGB	-4.43692	1.79E-06	SCNN1A	-3.35402	7.57E-05
INHBE	-2.80944	3.72E-05	BAAT	-4.31482	9.51E-06	PCSK9	-3.29709	9.86E-05
BEX2	-2.73076	1.97E-05	APOA1	-3.76551	2.63E-05	ITIH2	-3.10875	4.79E-05
DDIT3	-2.72919	0.000448	ADH1A	-3.74998	1.38E-05	ORM2	-2.99133	5.42E-05
SNCA	-2.67782	1.42E-05	ACSM2B	-3.70385	4.89E-05	NPPB	-2.95698	2.84E-05
IGFBP7	-2.66694	7.58E-05	C6	-3.64912	1.63E-05	MIR503HG	-2.94155	0.001241
ANKRD1	-2.63154	5.68E-05	PACSIN1	-3.63626	0.00017	INSIG1	-2.7059	0.000293
TRIB3	-2.62198	2.83E-05	NR1H4	-3.59572	0.000163	AHSG	-2.69999	4.32E-05
WARS	-2.55631	1.51E-05	ZNF385B	-3.54521	1.10E-05	SAA4	-2.69641	2.34E-05
EFEMP1	-2.52211	4.39E-05	GJB1	-3.53063	6.42E-06	THBS4	-2.6409	6.44E-05
SLC1A4	-2.501	1.51E-05	LGALS2	-3.45017	2.99E-05	HPX	-2.60383	2.84E-05
CALB2	-2.4151	1.49E-05	APOH	-3.37241	2.63E-05	EPYC	-2.58412	3.88E-05
HLA-DPA1	-2.40436	0.000127	PRODH2	-3.19559	9.84E-05	INHBE	-2.57718	2.42E-05
STC2	-2.3358	2.82E-05	HABP2	-3.19312	1.63E-05	SPACA6	-2.50555	0.001472
CXCL12	-2.25895	0.000107	KIF12	-3.19181	2.35E-05	EFHD1	-2.46677	0.000214
OLMALIN(	-2.23891	0.001073	ADH1C	-3.16985	6.77E-05	ORM1	-2.46269	0.000134
SARS	-2.1931	6.10E-05	UGT2B11	-3.13405	0.000242	CD14	-2.34675	0.000269
PCK2	-2.1501	0.00012	MTTP	-3.10449	0.000144	OLFML3	-2.3457	8.33E-05
MOXD1	-2.10274	7.58E-05	SERPINA6	-3.0867	6.18E-05	LHPP	-2.27467	0.000252
RASGRP3	-2.09204	5.45E-05	GSTA2	-3.0202	0.000269	MATN3	-2.1992	2.70E-05
FAM129A	-2.04806	9.87E-05	GSTA1	-2.94437	1.61E-05	HIST1H2BI	-2.1964	0.000383
IRS2	-2.03533	1.92E-05	CLRN3	-2.8867	4.91E-05	GPAM	-2.18167	0.000325
SULT1C4	-2.01583	4.51E-05	HTRA1	-2.88007	9.84E-05	H19	-2.16514	0.000888
KIF1A	-1.98129	3.37E-05	CALB1	-2.84852	1.61E-05	SLC7A10	-2.16487	0.00019
CGA	-1.97898	0.00099	ORM1	-2.75527	2.35E-05	FGL1	-2.16019	0.001625
SLC22A15	-1.97226	9.56E-05	RNASE4	-2.7009	6.95E-05	INHBB	-2.15627	0.000416
PDGFD	-1.96271	0.000107	CDH6	-2.69924	9.51E-06	MSMO1	-2.13781	0.000301
PRSS16	-1.95974	7.58E-05	ALB	-2.65835	2.35E-05	TRIB3	-2.13131	2.33E-05
LOXL4	-1.95867	9.93E-05	CFI	-2.61437	9.94E-05	FASN	-2.11383	0.000315
PDK4	-1.94204	0.000127	GLTPD2	-2.60206	0.000176	ABCA1	-2.11333	4.29E-05
NUPR1	-1.91196	0.0001	SEPP1	-2.5941	5.80E-05	F12	-2.11117	0.000346
AKNA	-1.90925	6.08E-05	UGT2A3	-2.57144	4.03E-05	ALPK2	-2.09514	0.00019
C9orf135	-1.87465	0.000197	ATP6V0A4	-2.5001	3.35E-05	LOXL4	-2.05566	5.79E-05
C8orf4	-1.87289	0.002879	DEFB1	-2.4892	1.42E-05	LOC10019	-1.99273	0.000483
PDE7B	-1.87275	3.77E-05	CFB	-2.42043	3.04E-05	RGS3	-1.98445	0.000493
CFTR	-1.85502	0.000282	VTN	-2.38758	6.86E-05	ANGPTL3	-1.979	5.42E-05
HYOU1	-1.84411	4.05E-05	CHST13	-2.34036	3.56E-05	CTGF	-1.96587	0.000134
ASCL1	-1.81956	0.000187	ASGR1	-2.33549	0.000955	MVD	-1.93789	0.000398
ASNS	-1.77899	0.000222	AMOT	-2.32815	0.000128	FAM150A	-1.9117	0.000145
SLC6A9	-1.76392	0.000252	ADGRV1	-2.32096	2.35E-05	MIR99AHC	-1.90051	0.000528
SESN2	-1.75078	0.006725	LIPC	-2.283	0.001237	NUCKS1	-1.89365	0.000301
TCF21	-1.73148	0.000732	VIL1	-2.26147	0.000163	HABP2	-1.84827	9.41E-05
LAMP3	-1.70611	7.58E-05	ERICH4	-2.24894	8.09E-05	ZMYM3	-1.84502	0.003438
GNG11	-1.70424	0.000222	METTL7A	-2.24712	0.000801	GABRE	-1.83495	0.004425
CSF2RA	-1.69661	0.000523	ACY3	-2.2418	0.000176	FZD2	-1.81352	0.000416
GBE1	-1.6762	0.000275	HAO1	-2.20518	0.000451	A2M	-1.80174	0.001252
CHN1	-1.66628	7.26E-05	SPTSSB	-2.19488	0.002656	LINC00261	-1.79633	0.000475
TCEA1	-1.65923	0.003212	IGFBP3	-2.1633	0.000426	APOB	-1.79452	0.000138
TUBE1	-1.65057	9.93E-05	DUSP9	-2.14266	0.002872	ASGR1	-1.79171	0.000205
SSUH2	-1.64801	0.000217	CDH12	-2.1232	0.001417	DCP2	-1.78155	0.000301
PHGDH	-1.64	0.000119	UGT1A10	-2.07519	0.00017	MIR181A2	-1.77073	0.000609

TPT1	-1.62743	0.000934	PLCXD3	-2.03729	0.000909	MAMLD1	-1.76601	0.000416
RSAD1	-1.61487	0.00057	ANKS4B	-2.02002	0.000144	OLMALIN(	-1.76478	0.000346
LYRM2	-1.61437	0.001926	ALDH1A1	-2.01327	0.000227	MICAL2	-1.76392	0.000206
EIF4EBP1	-1.61377	0.000148	ZMYND15	-2.00914	0.002186	TEX15	-1.74977	0.000139
XPOT	-1.59519	0.000593	MAOB	-1.96517	0.000734	DLC1	-1.72432	0.000781
WWC3	-1.5886	0.000491	PIPOX	-1.96078	7.28E-05	IRX3	-1.71928	0.000339
SOCS2	-1.57369	0.00013	PAPLN	-1.95861	0.000388	SDC2	-1.69963	0.000193
PSAT1	-1.57107	7.48E-05	MACROD1	-1.95383	6.33E-05	GPX8	-1.69045	0.001872
MTHFD2	-1.56155	0.000264	ANXA4	-1.94202	0.002258	EEF1A2	-1.683	0.000212
FST	-1.55725	0.000123	EPHX2	-1.93584	9.44E-05	ACAT2	-1.68209	0.000316
ZFAS1	-1.55619	0.006225	COL21A1	-1.91886	0.003913	ARHGAP3:	-1.67816	0.003368
FLRT2	-1.55153	0.00014	SERPINF1	-1.91122	0.001641	ENPP2	-1.67288	0.00034
SLC38A1	-1.53548	0.000127	CDH17	-1.8898	0.000345	ANXA9	-1.65665	0.001899
TMEM45A	-1.52896	0.002792	FOLR1	-1.88054	3.48E-05	SHC2	-1.65415	0.004814
ERO1B	-1.51914	0.000127	NCALD	-1.87057	6.00E-05	FADS1	-1.65204	0.000293
ULBP1	-1.51104	0.000123	ANG	-1.85459	0.000147	DGAT2	-1.64185	0.002338
SOX21	-1.50634	0.000539	CXCL2	-1.85374	0.000688	NES	-1.62597	0.000324
AP1S2	-1.48674	0.000103	IFITM1	-1.84624	0.000941	PAH	-1.61266	0.000825
ARG2	-1.48528	0.000295	PDZK1	-1.83998	0.000273	NEU4	-1.6118	0.000781
HIST1H4K	-1.48323	0.001423	AMBP	-1.83215	0.000329	ADARB1	-1.60508	0.00019
ZFP69B	-1.47047	0.00376	ALDH6A1	-1.8202	0.000819	BGN	-1.60381	0.000416
HTRA1	-1.46809	0.001806	BBOX1	-1.80934	7.55E-05	EBP	-1.58979	0.001125
CCPG1	-1.46422	0.000148	EHHADH	-1.79952	0.000896	TMSB15A	-1.58952	0.000826
CADPS2	-1.46122	9.00E-05	RAB17	-1.78799	0.001048	TESC	-1.58518	0.000212
UBASH3B	-1.46117	0.000469	LAMA5	-1.77239	0.000227	LGALS14	-1.57552	0.003824
ANGPTL8	-1.46023	0.014238	VWA5B2	-1.72044	0.001033	NUPR1	-1.558	0.000609
FAM127B	-1.45189	0.000295	ITIH2	-1.71898	0.01539	LAMA5	-1.54973	0.000609
SLC33A1	-1.45049	0.000147	CD24	-1.71005	0.000132	PDGFRA	-1.54351	9.41E-05
PYCR1	-1.44516	0.007975	ASB9	-1.68951	0.004054	AP1M2	-1.54298	0.003325
HKDC1	-1.44422	0.000174	AKR1C4	-1.6861	0.000906	FAM84B	-1.54204	0.000285
NEBL	-1.44206	0.00132	CXXC4	-1.68318	9.39E-05	TMPRSS6	-1.53324	0.004394
P3H2	-1.44148	0.000762	GNGT1	-1.65725	0.000266	RAB17	-1.51188	0.002751
CTGF	-1.43829	8.20E-05	PROC	-1.64165	0.006354	ZBTB8OS	-1.50891	0.001173
HSPA5	-1.42789	0.000127	SERPINF2	-1.62573	0.003597	CADM1	-1.50428	0.006201
LYPD6B	-1.42743	0.002655	AKR1B15	-1.62284	0.000778	XIST	-1.50216	0.0011
LOC72997	-1.4268	0.000309	TSPAN8	-1.61015	0.000493	AGO2	-1.49475	0.001285
CUL4B	-1.42642	0.000892	DBP	-1.60978	0.000569	AQP10	-1.48099	0.00045
WIPI1	-1.42463	0.000372	SLC17A3	-1.60475	0.001233	MVK	-1.47568	0.002685
KRT19	-1.41818	0.000675	CYP2C18	-1.6023	0.003989	TMEM170I	-1.47541	0.001473
SYBU	-1.41529	0.000479	HPGD	-1.59767	0.000905	ENAH	-1.47277	0.003074
IARS	-1.41381	0.000123	RARRES2	-1.59735	5.26E-05	RNASE4	-1.4713	0.001332
MTHFD1L	-1.41244	0.000127	GJB2	-1.58097	0.001329	GYG2	-1.46849	0.002993
TRIM61	-1.41157	0.001968	NPB	-1.58047	0.00297	HNRNPA2	-1.46519	0.004655
ATOH8	-1.39623	0.000239	SAMD11	-1.57917	0.000269	ANGPTL2	-1.46194	0.001682
TTC17	-1.3892	0.000158	FCGRT	-1.57662	6.00E-05	PROC	-1.45356	0.004322
MED8	-1.38615	0.000684	AGT	-1.57621	0.001164	IDI1	-1.43829	0.000983
TMEM156	-1.38592	0.000127	SULT1E1	-1.56452	0.000975	E2F7	-1.438	0.000315
TP53INP1	-1.38545	0.000114	UGT1A6	-1.56069	6.18E-05	ACSL3	-1.43753	0.006377
ZHX2	-1.38345	0.000641	AKR1C3	-1.55213	9.44E-05	NSDHL	-1.43622	0.000262
KRT81	-1.38197	0.010519	TYMSOS	-1.55137	0.001012	KIF12	-1.43479	0.002839
GARS	-1.38089	0.000295	RNLS	-1.53757	0.000555	DPYSL3	-1.42926	0.000528
RRAGC	-1.37969	9.56E-05	KCNF1	-1.53718	0.001767	EPHA1	-1.42838	0.021132
ZNF664	-1.37279	0.00305	LRRC31	-1.53585	0.004137	COL4A1	-1.42642	0.000239
TFAP2C	-1.37205	0.00143	SLC39A5	-1.53086	0.035199	CXCR4	-1.4214	0.005668
MOSPD2	-1.37151	0.000191	MAOA	-1.52774	0.00062	SREBF1	-1.42013	0.001325
CEBPG	-1.37125	0.000127	WDR72	-1.52542	0.000132	GAS2L3	-1.4186	0.001231
FAIM	-1.36863	0.000448	AFP	-1.51866	0.00026	XPOT	-1.41713	0.005944
QKI	-1.36362	0.001966	CTAG1A	-1.50715	0.000776	SNORD31	-1.41578	0.003956

PELI1	-1.3508	0.000378	HHLA2	-1.49439	0.009689	GLIPR2	-1.41371	0.000193
TFPI2	-1.34962	0.002703	GHR	-1.47786	0.001641	SMCR5	-1.40762	0.000794
RPL13AP6	-1.34487	0.001447	PPFIBP2	-1.46741	0.00017	CGNL1	-1.40045	0.000346
SHMT2	-1.34306	0.000222	PRDM1	-1.46518	0.000277	CACNA1H	-1.39533	0.001007
SLC39A11	-1.34008	0.000805	PROS1	-1.43683	0.000809	CEBPA	-1.39386	0.000251
EIF1	-1.33194	0.000269	PLEKHS1	-1.43446	0.000438	MYO5C	-1.39133	0.002212
GEMIN8	-1.32875	0.000468	SLC44A3	-1.42545	0.00073	SQLE	-1.39021	0.007236
ADAM9	-1.32825	0.000661	ARSE	-1.42361	0.000776	IFFO2	-1.38534	0.001132
CRIM1	-1.32719	0.000847	RHOU	-1.42247	0.000481	ZNF695	-1.3849	0.021641
FKBP10	-1.32443	0.001804	IGSF9	-1.41431	0.000434	CRIP2	-1.38425	0.014036
TCEAL9	-1.31682	0.006756	CDHR2	-1.41372	0.004537	SMAD6	-1.381	0.001959
MFGE8	-1.31491	9.00E-05	GSTM4	-1.40475	0.000327	ZNF786	-1.3807	0.001899
DOK6	-1.31052	0.000822	CCL2	-1.40341	0.000707	PAXIP1-AS	-1.36673	0.001131
SLC7A1	-1.30788	9.87E-05	UGT2B10	-1.40261	0.000865	LIG1	-1.36487	0.001458
LYRM4	-1.30463	0.001367	SMOC1	-1.40106	0.022408	THBS1	-1.35863	0.001641
TBCK	-1.29956	0.000503	TMEM86B	-1.39691	0.000183	TMEM184A	-1.35656	0.019835
GFPT1	-1.29695	0.000168	CNTNAP1	-1.38099	0.000566	UGCG	-1.35582	0.004734
SYP	-1.29656	0.000302	ALG1L	-1.37559	0.001681	POTEE	-1.35411	0.005693
MTMR4	-1.28888	0.000232	DBNDD1	-1.37111	0.00733	HMGB3	-1.35402	0.001237
IDH1	-1.2876	0.000237	PKP3	-1.37042	0.004281	DHCR7	-1.35366	0.000447
SBDSP1	-1.28453	0.000786	NAT8B	-1.36943	0.004023	CCND1	-1.34909	0.002422
GCC1	-1.2842	0.000188	APOM	-1.36915	0.019654	COL4A5	-1.34728	0.001146
PIEZO2	-1.28081	0.000711	LRG1	-1.36531	0.001641	DYNC1H1	-1.34205	0.000193
FAM92A1	-1.28076	0.018178	COL4A5	-1.35634	0.001948	DACT2	-1.3413	0.001078
POGLUT1	-1.28041	0.009257	STARD10	-1.35399	0.001185	FMO1	-1.33754	0.028761
BEX3	-1.27829	0.000786	AKR1B10	-1.35136	0.000572	MIR1228	-1.3356	0.014234
PLPP7	-1.27033	0.001643	DDC	-1.35036	0.002065	HHEX	-1.32934	0.000315
GNL3	-1.27032	0.000259	LCN2	-1.3489	0.001073	GADD45B	-1.32652	0.002577
UCA1	-1.27024	0.00143	UBD	-1.33475	0.000969	PLD5	-1.32541	0.000524
KRT80	-1.26686	0.002271	MOB3B	-1.3315	0.001935	ASAP3	-1.3237	0.000528
PYGB	-1.26675	0.000145	ACSM3	-1.33049	0.005137	ZMYND15	-1.32182	0.012717
H1F0	-1.26519	0.000583	TMEM37	-1.33044	0.00049	LOC10013	-1.31821	0.002839
DKFZP564	-1.26227	0.000156	GBA3	-1.32738	0.001012	SLCO4C1	-1.31577	0.000559
SPIRE1	-1.26193	0.000301	GLYCTK	-1.32011	0.000637	HNRNPDL	-1.31382	0.002876
MARS	-1.26058	0.00136	UGT2B17	-1.31216	0.00416	IGF2	-1.3073	0.000399
RND3	-1.26038	0.000355	NFKBIZ	-1.30609	0.004413	APOA1	-1.30548	0.002462
PDE4B	-1.25646	0.0032	ACADS	-1.29986	0.007791	SMARCD3	-1.3008	0.000624
BCAT1	-1.25645	0.000458	CRYL1	-1.29907	0.000778	F7	-1.29459	0.009451
TRUB1	-1.25541	0.001583	ABCB6	-1.29531	0.000572	ZAK	-1.28623	0.000641
SMOX	-1.25304	0.000197	BAIAP2L2	-1.28599	0.000637	SLC51B	-1.28554	0.010582
TCEAL3	-1.25249	0.000148	SCD	-1.28241	0.000149	CREB1	-1.28267	0.009039
HIST1H4J	-1.25214	0.002155	KLKB1	-1.28113	0.003457	DCDC2	-1.28233	0.016142
CTBP2	-1.24399	0.000574	HNF4A	-1.27615	0.019053	CXXC5	-1.28109	0.002042
C12orf76	-1.24389	0.001275	TNFSF10	-1.27513	0.005683	PCDHB19P	-1.27992	0.044366
RAB3IL1	-1.2335	0.000173	GSTA5	-1.2638	0.004245	AASS	-1.27693	0.002459
ARMCX6	-1.23228	0.004139	SMIM24	-1.26307	0.032112	ZNF281	-1.27572	0.004611
OXNAD1	-1.23118	0.021243	PTOV1	-1.26283	0.000426	GC	-1.27487	0.000369
PLAU	-1.22917	0.001008	FOS	-1.26072	0.000548	GRB7	-1.2731	0.001998
FADS1	-1.22267	0.000412	ATP1B1	-1.25711	0.001012	SPC24	-1.27251	0.001682
STT3A	-1.22069	0.000516	MCCC1	-1.25206	0.000625	SKA2	-1.26899	0.001048
GPT2	-1.21144	0.000125	SEMA3B	-1.24915	0.016423	DKFZP564	-1.2689	0.001722
EPB41L4A	-1.2109	0.007164	TMEM213	-1.24826	0.001203	CENPF	-1.25814	0.005611
IL18R1	-1.21027	0.000902	GTSF1	-1.24703	0.00295	LBR	-1.25675	0.001682
INHBB	-1.19971	0.000918	LRIG3	-1.24632	0.012575	NGEF	-1.25624	0.00207
CSRP1	-1.19933	0.000384	SLC3A1	-1.23492	0.013265	NPM2	-1.25566	0.007877
DHCR7	-1.19713	0.001495	ACMSD	-1.23373	0.008781	TP53INP1	-1.24943	0.004912
PRKAR1A	-1.19479	0.000266	TMEM170B	-1.22407	0.00394	PPL	-1.24909	0.007737
C12orf57	-1.19115	0.000178	HNF4G	-1.223	0.006234	ST3GAL6	-1.24548	0.000815

UAP1L1	-1.18689	0.007176	HADH	-1.21985	0.000327	USP49	-1.24454	0.005367
LYSMD1	-1.18627	0.01105	RUNDC3B	-1.21939	0.005923	HMGCR	-1.23979	0.000646
MSMO1	-1.18618	0.000456	SCN9A	-1.21004	0.000227	LRRC20	-1.23849	0.00034
KIF5C	-1.18559	0.000513	CFD	-1.20948	0.002532	FLRT2	-1.23688	0.003537
BCL7B	-1.18409	0.001162	AGMAT	-1.20672	0.000664	LRCH4	-1.23595	0.001981
TPP1	-1.18374	0.000425	SREBF1	-1.20203	0.005196	NID1	-1.23541	0.001943
CCNDBP1	-1.18314	0.001109	NREP	-1.1991	0.000906	WDR74	-1.23529	0.016044
IL11	-1.18136	0.000461	HLF	-1.1953	0.012436	HYOU1	-1.2351	0.038353
TNFRSF12/	-1.18109	0.001864	HNMT	-1.19499	0.014964	PHACTR2	-1.23241	0.000383
ITGB1	-1.18101	0.000259	RARB	-1.19052	0.004282	ASGR2	-1.23141	0.001682
HNRNPDL	-1.17994	0.001417	CX3CL1	-1.1854	0.000572	SAMD11	-1.23028	0.007101
PHF1	-1.17898	0.001661	TDO2	-1.16911	0.001012	FRAS1	-1.22924	0.001819
NRP1	-1.17724	0.000788	COL4A6	-1.16861	0.000269	TNNC1	-1.22824	0.005622
UBL7	-1.17709	0.002139	CDK14	-1.16792	0.008882	LYL1	-1.22475	0.003726
MOSPD1	-1.17177	0.00132	LINC0052(	-1.16552	0.01195	PRKD1	-1.22228	0.001903
ARHGEF2	-1.16663	0.000869	BTG1	-1.16498	0.000231	S100A4	-1.22169	0.002943
BET1	-1.16583	0.006182	MUC13	-1.1643	0.019851	TLN1	-1.22162	0.002622
UMAD1	-1.15895	0.003689	FOXA1	-1.16262	0.000572	MICALCL	-1.22104	0.045073
SIAH2	-1.15594	0.000572	REEP6	-1.16131	0.006786	SLC25A18	-1.2204	0.002834
SQLE	-1.15567	0.000503	MATN2	-1.16069	0.001377	NAT8B	-1.22035	0.006334
FGF7P6	-1.15445	0.00174	TBC1D19	-1.16062	0.002969	SCARA3	-1.22014	0.006877
PROM1	-1.15143	0.00205	EDN1	-1.1593	0.000906	HAUS2	-1.21963	0.004447
AP1G2	-1.14649	0.000679	TCEA2	-1.15879	0.013265	HSD17B7	-1.21881	0.004393
CRY1	-1.14446	0.000762	TRIM15	-1.15861	0.000471	SNORA61	-1.21834	0.001931
CFDP1	-1.1435	0.009509	SPDEF	-1.15776	0.001523	TRPC4	-1.21698	0.005367
YWHAZ	-1.13907	0.009687	C1orf115	-1.14756	0.00054	BEGAIN	-1.21164	0.004772
CALU	-1.13888	0.000734	DDAH2	-1.14577	0.006782	IPO7	-1.20762	0.021684
SNAP23	-1.13661	0.000503	STK3	-1.14491	0.001377	BLZF1	-1.20563	0.03231
DCAF4L2	-1.1324	0.005909	ADRA2B	-1.14191	0.004318	IGFL3	-1.20561	0.004339
SUPT3H	-1.12387	0.014893	ANXA3	-1.14051	0.036584	KRT23	-1.20498	0.000597
ALDH6A1	-1.12248	0.001926	PKDREJ	-1.14041	0.016138	BCAT1	-1.19812	0.002057
GPR89B	-1.12126	0.009123	PFKL	-1.13592	0.008559	PAFAH1B3	-1.19808	0.000315
LGALS3BP	-1.11413	0.001355	PIK3C2G	-1.13535	0.000762	PDGFRL	-1.19781	0.00206
C5	-1.11368	0.000731	KLHDC2	-1.12515	0.00434	SRCAP	-1.19558	0.004447
DESI2	-1.10996	0.000746	ZBTB44	-1.12317	0.001549	CBL	-1.19543	0.008492
CBX1	-1.10934	0.00918	AQP11	-1.11877	0.001225	SNORA70	-1.1924	0.022353
SLC1A5	-1.10891	0.000394	MYL5	-1.11491	0.005211	FAM72D	-1.19232	0.004877
MCFD2	-1.10433	0.002297	NIPSNAP1	-1.11029	0.00041	SCD	-1.19191	0.002287
MCRIP1	-1.10432	0.00484	CDKN1B	-1.10702	0.022254	TSPAN4	-1.19129	0.00042
CSRNP2	-1.10307	0.001478	PRDM11	-1.10521	0.012626	ACLY	-1.19114	0.005248
AHI1	-1.10229	0.002055	C5	-1.09783	0.000448	ARAP3	-1.19039	0.003613
FRAS1	-1.10194	0.003191	POLR1D	-1.09751	0.001523	SNORD14/	-1.18968	0.030599
TMIE	-1.10186	0.001515	KRT24	-1.09636	0.00751	PRRC2C	-1.18724	0.004934
ARHGAP9	-1.10062	0.026035	UGDH	-1.09556	0.001312	GINS2	-1.1856	0.000313
PNPLA8	-1.10014	0.007885	ACPP	-1.09231	0.002019	CDK14	-1.1855	0.007191
C21orf58	-1.09444	0.023788	RARRES1	-1.0923	0.005337	FARP1	-1.18267	0.001466
TES	-1.09334	0.004198	LACTB2	-1.08799	0.002656	UBR4	-1.18008	0.000659
C5orf51	-1.09174	0.000661	PCDH20	-1.0845	0.016762	EIF3CL	-1.17995	0.000716
ZFAND1	-1.09146	0.001256	TNFRSF11I	-1.07985	0.022834	C1orf162	-1.17464	0.01616
BEST1	-1.09145	0.000187	PLXNB1	-1.07694	0.009196	SLC1A3	-1.17224	0.001869
CYBRD1	-1.09059	0.002094	TCEA3	-1.07393	0.000846	TCEAL3	-1.16738	0.036309
UFM1	-1.08685	0.000922	FAM117B	-1.07192	0.001958	NPC1L1	-1.16681	0.001015
TBC1D4	-1.08554	0.000464	DDN	-1.06892	0.004054	TGFB2	-1.1647	0.004772
FAM163A	-1.083	0.000812	RPS23	-1.06885	0.000572	VASH2	-1.16256	0.018652
BCL2L2	-1.08108	0.002094	SGSH	-1.06678	0.001377	PAQR9	-1.16241	0.002334
TICAM2	-1.08069	0.004866	TUBD1	-1.06614	0.007252	NAALAD2	-1.15893	0.005762
C10orf54	-1.07978	0.002036	RASL11A	-1.06359	0.022227	TMEM178/	-1.15887	0.005933
ZFAND3	-1.07963	0.007514	ALDH5A1	-1.06121	0.001994	FBN1	-1.15776	0.00684

RNF128	-1.07686	0.001393	INTS3	-1.05679	0.001958	ARHGAP32	-1.15695	0.001626
EPB41L5	-1.07624	0.002134	FAM183A	-1.05198	0.009465	APOC3	-1.15273	0.005464
GLI1	-1.07562	0.001211	ENPEP	-1.05113	0.03269	ZNF519	-1.15163	0.025298
CHST3	-1.07515	0.003846	PTBP2	-1.04741	0.01637	BOLA2	-1.15161	0.017535
COG5	-1.07305	0.001827	MYRF	-1.04607	0.005902	FOXM1	-1.15127	0.005935
ZFAND6	-1.07242	0.007902	CFLAR	-1.03584	0.001064	S100A16	-1.15021	0.002751
GALK2	-1.07205	0.00118	ELF3	-1.03531	0.018657	SNORA25	-1.14992	0.002763
SLC25A6	-1.07146	0.000259	TBCD	-1.03058	0.010989	COL4A6	-1.14979	0.001111
PDE3A	-1.07012	0.000927	TMEM135	-1.0303	0.013933	C1orf54	-1.14963	0.002811
RBM24	-1.06856	0.002967	DDAH1	-1.02812	0.012963	EVI5	-1.14917	0.003465
ATXN10	-1.06815	0.001187	FUOM	-1.0262	0.020089	ANLN	-1.14805	0.001981
COPG1	-1.06644	0.004417	ZNF395	-1.0254	0.000896	FDFT1	-1.14726	0.00522
MFSD14A	-1.06444	0.020868	COQ8B	-1.02436	0.001047	TMEM97	-1.1468	0.001241
APPL2	-1.06319	0.000875	PCCA	-1.02298	0.000688	DUSP9	-1.14537	0.01172
DUSP6	-1.0625	0.000889	TMEM97	-1.02185	0.000732	TTC39C	-1.1413	0.000493
BAAT	-1.05874	0.000458	ZNF618	-1.02063	0.00141	KCNJ8	-1.14103	0.001213
DSC3	-1.05679	0.00129	SLC27A1	-1.02027	0.024213	SPAG5	-1.13946	0.007681
CTSL	-1.05427	0.007097	SF3B3	-1.01689	0.003375	PAQR4	-1.13699	0.00049
SUMF2	-1.0541	0.000406	PGAP3	-1.01572	0.028795	MEF2C	-1.13226	0.008471
HAX1	-1.05211	0.002659	PCYT2	-1.01568	0.000922	GPD1L	-1.13209	0.001016
KRT23	-1.05049	0.008883	WNT4	-1.00813	0.04114	CNIH2	-1.13164	0.009191
MPST	-1.0497	0.001547	SACM1L	-1.0044	0.006354	ID4	-1.13092	0.021754
HEG1	-1.04824	0.00305	CAND1	-1.00189	0.006786	HSPA5	-1.1302	0.000324
RHOQ	-1.0479	0.000747	ACOT2	-1.00077	0.005137	CYP51A1	-1.12799	0.000566
FAM127C	-1.04788	0.037833	ITGB5	1.002863	0.000976	FANCG	-1.12536	0.000609
CHST4	-1.04771	0.000267	TM4SF18	1.004162	0.000955	LMOD3	-1.12463	0.006005
PPARGC1A	-1.04437	0.002055	LETM2	1.005803	0.020754	S100A9	-1.12444	0.003753
ARL4A	-1.04363	0.001404	BCAR3	1.011315	0.001958	CPB2	-1.12388	0.000321
TARS	-1.04098	0.001261	CAV1	1.013549	0.001538	CTXN1	-1.12352	0.005378
ANXA3	-1.03952	0.010028	SNORA61	1.014811	0.003005	TMEM191A	-1.12289	0.016179
PLAGL1	-1.03908	0.002968	SPINK13	1.023626	0.005196	NRP1	-1.12102	0.003347
MANBA	-1.03585	0.000412	ULK1	1.025228	0.004702	CLCN6	-1.1199	0.002577
USE1	-1.0349	0.019395	PLP2	1.027719	0.001338	QKI	-1.11522	0.027979
AJUBA	-1.03372	0.007611	CD82	1.03227	0.005463	JAG1	-1.11187	0.001048
PPP2R5B	-1.03236	0.007957	TGM2	1.033549	0.005496	PRR11	-1.10975	0.003933
C1orf43	-1.03204	0.001905	IRS2	1.03506	0.00159	PHGDH	-1.10953	0.013561
SCD	-1.03002	0.002408	2-Mar	1.0357	0.00159	PTAR1	-1.10766	0.000729
AFF4	-1.02861	0.009232	GPRC5A	1.036044	0.011583	HMGCS1	-1.09494	0.000946
TMEM39A	-1.02394	0.000557	LACTB	1.039517	0.004511	APOA2	-1.09419	0.014788
SMIM13	-1.02282	0.026939	ARPC1B	1.03964	0.001366	HIST1H4C	-1.09353	0.016249
ASB3	-1.02094	0.003171	IRAK2	1.041276	0.00289	FEN1	-1.09271	0.001401
INSIG1	-1.01761	0.002883	ARHGEF2	1.041441	0.001064	CYP4F3	-1.09267	0.000786
MORN4	-1.01521	0.027164	CD9	1.042029	0.014494	TTR	-1.0918	0.002287
RASSF9	-1.01438	0.01968	MICB	1.043443	0.00387	PHKA2	-1.09063	0.005817
KRT10	-1.01421	0.007363	CCDC84	1.043966	0.002099	RBMS2	-1.08859	0.002454
ATF4	-1.01411	0.000546	FBXO32	1.044973	0.004252	SOX9	-1.08777	0.000416
BBS10	-1.01388	0.005331	FKBP14	1.048759	0.001437	ALPP	-1.08413	0.000492
IFRD1	-1.012	0.001293	KLF2	1.049	0.001125	ASNS	-1.08313	0.001235
TMEM55B	-1.01026	0.004543	MYH9	1.053963	0.000688	GLTPD2	-1.08254	0.003134
PRMT1	-1.00911	0.013442	RNU4-2	1.057597	0.010875	SUGP2	-1.08121	0.016865
ZSCAN9	-1.0058	0.001904	EEPD1	1.059746	0.007086	KIF15	-1.07871	0.002195
CLCN6	-1.00553	0.003922	RNA18S5	1.061884	0.001829	SGO1	-1.07395	0.000455
HRK	-1.0001	0.002136	FAM89B	1.067296	0.001331	FBXO4	-1.07306	0.001532
CDK1	1.000276	0.013411	SMOX	1.073099	0.025556	LOC64493	-1.07227	0.005447
GAGE2E	1.002714	0.037555	GAD1	1.075502	0.003458	HUWE1	-1.07135	0.002763
NACC2	1.004753	0.006749	EAF2	1.078834	0.005303	NANOS3	-1.07068	0.013999
TLE3	1.00627	0.002601	LMLN	1.079412	0.030943	NR0B2	-1.07017	0.002306
AURKA	1.006299	0.000456	FBXL13	1.080496	0.004662	LPIN1	-1.06995	0.00162

GAGE7	1.008132	0.00241	TGFBI	1.08223	0.000538	MYCN	-1.06786	0.030505
TRIP13	1.008921	0.000287	SLCO3A1	1.083566	0.043871	BIRC6	-1.06781	0.014982
TYMS	1.010498	0.000762	CAPRIN2	1.088077	0.000398	OAT	-1.06566	0.000946
AURKB	1.010622	0.001729	PID1	1.089202	0.00196	PYCR1	-1.06115	0.009128
PHEX	1.010974	0.008107	B4GALT1	1.092586	0.023344	RGN	-1.06048	0.017535
ZCCHC14	1.011914	0.004635	CD151	1.092746	0.001599	UAP1L1	-1.0586	0.012871
MGEA5	1.012674	0.000918	RUNX2	1.098296	0.00733	MFSD14C	-1.05626	0.002719
TNFAIP3	1.013069	0.001747	POLD4	1.100404	0.030284	A1CF	-1.05404	0.013524
PBK	1.013677	0.000523	TNFRSF10I	1.106326	0.001349	MCM7	-1.05223	0.001523
HIST2H2A.	1.014082	0.002335	TTC14	1.108235	0.003108	SLC1A7	-1.04923	0.004611
ERBB3	1.015355	0.002134	CSF1R	1.108733	0.004483	NFIX	-1.04838	0.00717
EDC4	1.016464	0.00871	ARID3A	1.113989	0.002491	LUC7L3	-1.04573	0.004499
ADH1A	1.016523	0.000305	DGKG	1.115939	0.013555	DDB1	-1.04482	0.012988
HIST1H1C	1.017155	0.004813	ISG15	1.116367	0.001338	SLC7A1	-1.04445	0.00254
FGB	1.017706	0.000701	LOC10192	1.117951	0.004079	SLC13A5	-1.04086	0.002392
CYB5A	1.024406	0.001118	CLDN2	1.118707	0.001125	LIN7C	-1.03705	0.026254
SMCO4	1.02468	0.002431	OGFRL1	1.119383	0.002128	6-Mar	-1.03593	0.003056
BRAT1	1.025072	0.003614	GALNT10	1.121797	0.002969	SYTL5	-1.03592	0.004877
WDR74	1.025424	0.003614	ASNS	1.122997	0.001098	CENPM	-1.03518	0.040706
FAM162A	1.025604	0.002691	SOX9	1.124378	0.002999	TBC1D16	-1.03136	0.002681
CAMSAP3	1.027116	0.001047	HMOX1	1.124924	0.000688	FDPS	-1.03077	0.001982
TUBB4B	1.02758	0.002691	RNU6-1	1.127603	0.000729	ADAMTSL4	-1.03008	0.049402
AK1	1.028133	0.005892	PROCR	1.128825	0.001543	STMN3	-1.02795	0.002141
CA9	1.029662	0.004042	TXNIP	1.131934	0.002484	BUB1B	-1.02765	0.034332
SKIDA1	1.029831	0.003082	FAM129B	1.136147	0.001042	EP400	-1.02734	0.000767
DYNLL2	1.032501	0.000448	MORC4	1.138455	0.017867	NT5C2	-1.02573	0.001912
NOTCH1	1.033101	0.035782	IER5	1.138841	0.01248	ACACB	-1.02055	0.001208
MAF	1.033812	0.001753	HES4	1.139528	0.00089	CTNND2	-1.01984	0.007201
DSE	1.035472	0.001545	HIST2H2A.	1.144252	0.014513	ARHGEF28	-1.01782	0.035341
ITGA2	1.035615	0.005385	SLC16A5	1.147228	0.005208	GALNT3	-1.01421	0.024226
ACSL4	1.036371	0.00041	CARD10	1.147573	0.000896	TET1	-1.01403	0.005248
ANKS4B	1.039174	0.000661	SMG1P1	1.15053	0.010262	PCED1B	-1.01216	0.006428
GNG4	1.045384	0.000907	MFSD14C	1.153783	0.002956	MMAB	-1.01089	0.04508
PKMYT1	1.045599	0.008809	ARNT2	1.158557	0.000505	THAP10	-1.0085	0.001764
SLX4	1.04572	0.001316	C10orf90	1.159513	0.001384	KLB	-1.00819	0.004121
METTL7A	1.046349	0.001495	TIPARP	1.16336	0.000906	NBEA	-1.00626	0.041428
SUN2	1.047435	0.012428	IDH3G	1.164978	0.020954	C9orf40	-1.0062	0.010064
PPDPF	1.047558	0.001995	ICAM4	1.169282	0.01044	ENO3	-1.00405	0.006877
CALD1	1.048178	0.001955	C15orf48	1.171588	0.003399	CHD7	-1.00085	0.000964
ZNF738	1.049576	0.014905	AGPAT2	1.177213	0.004307	G2E3	-1.00019	0.013322
MYO1A	1.049891	0.007282	RBMS2	1.177304	0.001945	AFAP1L1	1.000514	0.00194
TF	1.050524	0.000275	MBP	1.178622	0.000905	TMEM179I	1.00106	0.001587
STIL	1.050717	0.00174	SLC35E4	1.179803	0.004085	IL10RB	1.001068	0.027043
SLC45A3	1.050849	0.000448	SNORA6	1.180003	0.006908	C4orf32	1.001334	0.002392
ACMSD	1.05201	0.003913	FAM200B	1.183116	0.001292	ADORA2A	1.001802	0.04982
CDS1	1.052519	0.004588	UCA1	1.185821	0.002614	AADAC	1.002176	0.012438
KBTBD11	1.053064	0.000915	SLC7A5	1.19269	0.000434	FSTL1	1.002785	0.014234
SSTR2	1.054796	0.035878	CRABP2	1.192814	0.001921	EFR3B	1.003109	0.005792
CDCA8	1.055073	0.002086	MVP	1.193328	0.000992	OCA2	1.003225	0.003352
DLGAP5	1.055618	0.000503	SARDH	1.195003	0.000555	PIK3IP1	1.00351	0.004295
HMGB2	1.05792	0.005916	SLC43A2	1.195656	0.001292	FAM134C	1.004253	0.001682
NOCT	1.058174	0.000766	NOTCH2N	1.196244	0.000664	CEACAM1	1.007498	0.005464
TM6SF2	1.058276	0.001779	MPRIP	1.198322	0.02449	NME2	1.009255	0.018322
CX3CL1	1.05862	0.005947	CRACR2B	1.201567	0.009465	NEIL2	1.009378	0.032537
RAD23B	1.060199	0.000269	TFF2	1.206272	0.004023	NFKBIB	1.010318	0.013605
CAMKV	1.060482	0.000939	NFKB2	1.211966	0.004023	MOXD1	1.015104	0.001196
SHPK	1.061851	0.002269	LARP6	1.220965	0.001338	RNU6ATAC	1.016148	0.030599
KIF20B	1.061969	0.000264	SAT1	1.227344	0.0033	RFPL4B	1.016919	0.002151

GAGE12J	1.062366	0.042879	IRF1	1.232591	0.005976	GNAI1	1.016953	0.004451
GC	1.062437	0.00221	CAPN2	1.238138	0.000328	RAB3C	1.018018	0.016441
DDIAS	1.067066	0.000953	LRP5L	1.245035	0.006164	C3orf58	1.018114	0.001772
CDCA5	1.067245	0.001696	STEAP1	1.248103	0.006647	ABCC5	1.023695	0.016179
CCDC106	1.068252	0.001281	WNT7B	1.256762	0.021161	LINC0113٩	1.02594	0.023208
CEBPZ	1.069158	0.001212	HSF2BP	1.265889	0.000598	GNA13	1.027631	0.001043
SMAD6	1.070607	0.001998	FSCN1	1.277936	0.000504	PELI1	1.027638	0.001582
GSG2	1.072321	0.000593	FAM20C	1.279708	0.001297	ZNF816	1.028093	0.001382
C7orf49	1.072896	0.001162	LHB	1.280349	0.000539	STX7	1.028793	0.002751
KIF15	1.073867	0.000824	IL15RA	1.280889	0.001042	RPL26	1.03	0.025541
RAB40B	1.07519	0.001204	GSDMB	1.290406	0.000457	OXSM	1.030011	0.003806
TICRR	1.076458	0.007747	GEM	1.30783	0.003756	HIST2H4B	1.03064	0.029933
TMEM91	1.078172	0.000847	N4BP2L2	1.310216	0.001681	SPTSSA	1.034045	0.002255
ACD	1.078308	0.000469	FSTL3	1.310861	0.006614	EMP1	1.034698	0.002608
PRRG2	1.078485	0.002227	IFRD1	1.312393	0.000784	BTG1	1.036215	0.000737
MAP6D1	1.078502	0.003522	IL1RAPL1	1.317321	0.001302	C6orf52	1.03699	0.001939
POLQ	1.079259	0.002847	MT1X	1.319927	0.001656	IRAK3	1.037385	0.019591
SEMA4D	1.080513	0.001696	PCED1B	1.321552	0.000555	LACTB2	1.038922	0.001235
DHX38	1.080812	0.001495	MYOF	1.324383	0.000571	RBP7	1.040142	0.044384
TLDC2	1.083123	0.000847	NNMT	1.33532	0.006912	NDP	1.04049	0.003074
BATF3	1.083202	0.001162	LRRC8E	1.344649	0.000647	GJC2	1.041044	0.012514
ZMYM3	1.084286	0.003202	CTSL	1.34915	0.000633	TRIM16L	1.041822	0.006428
SLC2A4RG	1.084722	0.002655	ALDH1A3	1.35257	0.013098	FHL2	1.042827	0.001575
GAGE12F	1.085595	0.042136	GPR3	1.354447	0.000647	BBS2	1.0432	0.004295
MPV17L	1.087692	0.003721	MAFF	1.359592	0.010766	METTL2B	1.044376	0.014159
FOXA3	1.08776	0.002691	VPS37D	1.36941	0.001048	WWC1	1.045605	0.001173
HOXB8	1.088349	0.001489	NKD2	1.371525	0.000548	TRAFD1	1.046265	0.000905
ZNF256	1.092726	0.000777	CD68	1.378829	0.000102	MPP6	1.049931	0.000912
KIAA0922	1.094572	0.011487	NCEH1	1.381945	0.001234	LIMA1	1.05093	0.007468
ZMYND15	1.094947	0.000458	MYEOV	1.384104	0.005048	MT2A	1.052497	0.010695
FHL2	1.09643	0.003171	TMEM156	1.390595	0.00733	TOX3	1.055442	0.00134
ID1	1.097591	0.001803	FHL2	1.391935	0.001933	PIM2	1.055834	0.007878
DAGLA	1.100754	0.009404	MOK	1.392036	0.000958	CD163L1	1.055883	0.001015
C1orf115	1.10097	0.000513	TRNP1	1.398086	0.000178	DUSP10	1.056346	0.030588
C1orf106	1.101233	0.008364	HEY1	1.399403	0.000117	UBE2D4	1.057862	0.000856
SEL1L3	1.101453	0.001207	PTK2	1.401403	0.000426	UXS1	1.059541	0.021077
TMEM8B	1.10162	0.019438	ALPK2	1.412139	0.005078	PRIM2	1.059759	0.030216
SSX3	1.102492	0.003116	CUEDC1	1.416995	0.000371	RPL29	1.061116	0.014965
HPN	1.102921	0.018926	SNORD3A	1.422974	0.000798	SP110	1.062122	0.004772
DDX46	1.104728	0.001132	MMP14	1.425403	0.000354	RNF145	1.06217	0.001772
CYP26B1	1.104989	0.003655	SIK1	1.426509	0.004888	ACBD5	1.063028	0.022675
PIGX	1.105352	0.015203	SLC9A7	1.433507	0.00338	ARMCX5	1.064112	0.006907
NUSAP1	1.106744	0.001204	IDS	1.455056	0.001529	ADRB2	1.065075	0.002338
MAT1A	1.106969	0.005831	MILR1	1.47217	0.002396	SLC38A4	1.066878	0.008086
HELZ2	1.106977	0.005475	SFR1	1.477087	0.001048	NNMT	1.068169	0.007242
KCNMB4	1.107105	0.006805	MMD	1.485083	0.000206	LGALS1	1.069525	0.02007
NFKBIA	1.107557	0.003754	SERPINA1	1.485274	0.00189	LRRN3	1.070278	0.001495
TRIM31	1.111747	0.000325	HSPA7	1.494279	0.004035	ZNF543	1.070572	0.01469
TMEM184/	1.113367	0.007074	BHLHE40	1.496555	0.000208	FAM86C1	1.070797	0.001458
ZNF215	1.11354	0.000818	CYP2S1	1.500567	0.001052	MAF	1.071049	0.002422
PLK1	1.113638	0.007172	CDA	1.507752	0.000144	LYRM5	1.071819	0.000664
AQP12A	1.114633	0.010617	FOXQ1	1.511296	0.000772	TMEM35B	1.077828	0.037784
DTL	1.115176	0.002464	MT2A	1.520424	0.000572	SLC18B1	1.077914	0.002042
NRGN	1.115938	0.002792	GPAT3	1.531976	0.000614	GDF15	1.082062	0.000884
ZNF684	1.11635	0.000762	SERPINB8	1.535065	0.007508	NAGPA	1.084627	0.001761
GAGE12D	1.116413	0.027448	KIAA1683	1.537369	0.002002	GM2A	1.090493	0.035341
ST3GAL2	1.12023	0.017713	C1orf106	1.544278	7.55E-05	PARP12	1.091782	0.000664
CD9	1.122168	0.009163	EPAS1	1.546261	0.00026	SLC31A2	1.093904	0.035275

LBP	1.122591	0.00539	SDSL	1.546291	0.000215	ZNF319	1.094656	0.001235
SERPIND1	1.1227	0.000296	ANPEP	1.558863	0.000664	TAP2	1.094832	0.010599
RAB13	1.122779	0.010226	TOX2	1.565695	0.000572	FRK	1.097921	0.004923
TIPARP	1.124478	0.002134	LYL1	1.577562	0.013265	PSMB10	1.100252	0.000415
GAGE2A	1.124813	0.018239	DDIT3	1.602512	0.001516	ACP6	1.100978	0.003155
TUBB2A	1.130761	0.000765	C15orf52	1.617875	0.000896	FOXQ1	1.102008	0.003753
GPR160	1.131089	0.000389	PLXNA1	1.636652	0.000409	PDCD2	1.103184	0.003537
CCNB1	1.132663	0.000389	ADORA2B	1.640657	0.000468	ABCG2	1.103315	0.003564
B4GALNT1	1.133485	0.002978	CACNA2D	1.645358	0.001758	OPTN	1.104578	0.006413
LOC81691	1.134442	0.001417	TSPYL2	1.660497	0.001028	RGS2	1.106032	0.002008
HES1	1.136462	0.000582	TNFRSF12.	1.680606	0.000316	EEF2KMT	1.106456	0.001682
KIAA1524	1.138561	0.001799	ZFAND2A	1.699266	0.00191	ATP1B1	1.106518	0.000416
DNAJC22	1.138757	0.000198	GAS2L1	1.700082	0.00753	MPP1	1.111543	0.014705
CD68	1.138855	0.000458	PHLDA2	1.708687	0.000227	FAM102A	1.111971	0.002699
GAD1	1.141075	0.000816	RNA28S5	1.714585	0.011793	EPHX2	1.113914	0.000649
RPL21	1.143733	0.034844	P2RY6	1.722831	0.000569	TMEM114	1.117024	0.001165
CXCL2	1.144794	0.001799	CPN2	1.734102	0.000369	IFI27L2	1.117379	0.038465
PHLDA2	1.146493	0.000479	RMRP	1.738539	0.000233	NCEH1	1.120072	0.017978
TGM2	1.14758	0.00108	PTHLH	1.741708	0.000132	IL12A	1.120543	0.003644
SLC37A4	1.150778	0.002269	LGALS1	1.747883	6.18E-05	PLBD1	1.122622	0.000506
NCAPG	1.15081	0.000583	SGK1	1.755645	0.000339	NETO2	1.122881	0.004629
CHAF1B	1.152313	0.002333	PANX2	1.761709	0.000896	EGR1	1.125759	0.001458
METTL7B	1.15282	0.001713	IL20RB	1.777106	0.000345	PLA2G16	1.127038	0.001722
APOBEC3E	1.153154	0.000892	HP	1.778471	0.000598	ISG15	1.127955	0.003755
AGMAT	1.15447	0.000466	SERTAD1	1.7865	0.000132	ALDH8A1	1.12896	0.00611
KIF11	1.157644	0.000789	ACOX2	1.787231	0.000327	HS6ST2	1.132712	0.005425
ONECUT1	1.162338	0.003171	TGFA	1.790468	0.001527	SLC44A3	1.133171	0.000896
SLC15A1	1.164322	0.00283	HSPA6	1.79412	0.000776	MTTP	1.134423	0.001523
KIF22	1.164762	0.002605	GRAMD1A	1.797451	0.001538	TTLL1	1.140608	0.002202
TOP2A	1.165489	0.000523	EPHA2	1.803352	7.28E-05	TLR7	1.142049	0.001903
CHST13	1.166172	0.000309	SNORA12	1.803421	0.001125	RBM47	1.142248	0.001625
KIF14	1.166222	0.000222	CLCF1	1.811898	0.000688	TRMT61B	1.142966	0.005246
FAM171A1	1.172085	0.000433	CNGB1	1.829127	0.000593	RAPGEF5	1.145408	0.000777
ATP8B1	1.172087	0.007125	UBR4	1.852933	4.44E-05	TSPYL2	1.146871	0.000912
KIF2C	1.17268	0.004139	SH2B3	1.878343	4.12E-05	GPNMB	1.148529	0.002787
ZFPM1	1.174033	0.001043	GDF15	1.889036	0.001917	SPSB1	1.149195	0.001819
ITGAL	1.174573	0.010794	FOSB	1.893205	0.00017	CAV1	1.151281	0.006556
NMT2	1.177007	0.004617	PPP1R15A	1.907333	0.000409	KDM3A	1.15132	0.037731
ARHGEF39	1.179479	0.004568	ITGA2	1.909954	0.00171	OAS1	1.153233	0.005721
CENPF	1.181151	0.001724	CST7	1.915074	0.000572	EHD4	1.155358	0.001285
DDX27	1.182668	0.000874	SPANXB1	1.920219	0.000287	GPAT3	1.157817	0.001813
GPX2	1.184072	0.008827	VEGFC	1.925217	0.000657	ICAM1	1.158693	0.0036
MCM4	1.186123	0.000441	RND1	1.927012	0.000327	WBSCR27	1.162686	0.00403
SSX5	1.186312	0.001595	RNU1-1	1.9378	0.000481	FTH1P3	1.167027	0.006231
BUB1	1.189971	0.000456	IL6	1.94119	0.00027	MGST3	1.17005	0.002278
CDC45	1.190202	0.000343	UBASH3B	1.954134	5.58E-05	IER5	1.171073	0.0053
IQCC	1.1905	0.008193	SNORD3D	1.967535	0.005078	P2RX4	1.173341	0.00635
CENPW	1.191832	0.001023	SCG5	1.985856	0.004936	CYSTM1	1.174588	0.001903
NSMF	1.193327	0.000661	CDKN1A	2.006309	0.000132	TBC1D9B	1.178239	0.007673
SERPINA6	1.195327	0.000217	SLC2A5	2.029806	0.000955	SPACA9	1.178676	0.003119
GSTO2	1.198247	0.001264	CSF2	2.040185	0.000313	STX4	1.178896	0.004909
POP1	1.198601	0.000305	SLC16A3	2.046047	0.000269	FOS	1.180369	0.002133
TMEM175	1.200268	0.001453	HBEGF	2.084029	7.79E-05	SOD2	1.182261	0.011883
PPP1R14A	1.201132	0.001731	HPCAL1	2.084506	9.46E-05	ZNF425	1.18387	0.001015
CDT1	1.20701	0.014557	SMAD3	2.089443	0.000572	TMEM182	1.184259	0.002763
GJB1	1.207114	0.00099	TSC22D1	2.10349	7.20E-05	STAT3	1.184522	0.004842
POMT1	1.210765	0.004238	PHLDA1	2.118155	0.000147	MAP7	1.185854	0.014036
LRG1	1.212672	0.002586	RNVU1-18	2.167569	0.001338	CCDC71	1.185943	0.00036

PLEKHG2	1.213342	0.000171	RNVU1-7	2.170362	0.000227	ISM1	1.191996	0.007027
ADM	1.213844	0.00081	SPANXC	2.176957	6.00E-05	RPL41	1.198046	0.011941
MET	1.214836	0.002696	RNU1-3	2.196465	0.000801	JADE2	1.198591	0.002334
SPAG5	1.215521	0.000448	DUSP5	2.198239	6.74E-05	CFH	1.20091	0.001007
C4orf19	1.215594	0.003564	C19orf33	2.237937	4.91E-05	HS3ST3A1	1.204405	0.006629
MED29	1.216079	0.00697	KRT80	2.245104	4.91E-05	NOV	1.207478	0.00049
CASC10	1.219705	0.000178	MLPH	2.252725	2.32E-05	FAM200B	1.207977	0.000459
KDM3A	1.220565	0.001146	AXL	2.254041	4.03E-05	HMGCS2	1.208454	0.005464
POLR2E	1.226817	0.002212	RIMBP3	2.288593	0.000572	SPPL2A	1.208571	0.002042
ARL14	1.228046	0.000854	SNAR-A1	2.29238	0.00107	KYNU	1.209423	0.001903
CCNF	1.228165	0.001148	LY96	2.297756	0.000192	AGTRAP	1.210078	0.002459
FTH1	1.228913	0.000894	RNU6ATA(	2.314793	0.000896	COA6	1.210251	0.007167
DEF8	1.240651	0.000776	SLC20A1	2.323537	5.38E-05	YJEFN3	1.215523	0.03352
SERPINF2	1.24116	0.001148	TFF3	2.345748	3.04E-05	TMEM106I	1.220868	0.001761
SLC44A1	1.242429	0.00049	TINAGL1	2.353677	0.000492	ACKR2	1.2216	0.000871
BCL6	1.244041	0.000719	FAM43A	2.354593	0.000285	TRIB1	1.2219	0.000293
RBM12	1.245535	0.003327	SERPINB2	2.392615	0.000271	PARP10	1.223265	0.001408
JAM3	1.246313	0.001303	SLC3A2	2.412624	0.000329	KLF4	1.223921	0.011351
RRBP1	1.248269	0.00343	SPANXA2	2.419858	0.000664	DTX3L	1.226563	0.001401
ETS2	1.249027	0.000237	NT5E	2.444446	5.36E-05	LRP10	1.231252	0.001078
RPL10L	1.251404	0.006419	LINC0070(	2.463229	5.80E-05	GALNT4	1.234796	0.000813
TTK	1.2524	0.000516	PTGES	2.470325	0.000131	LGALS3BP	1.235705	0.002475
EXO1	1.254034	0.0014	KIAA1324	2.509118	2.63E-05	SLC22A18,	1.237789	0.005762
TCP10L	1.255469	0.007079	GADD45B	2.542289	3.48E-05	RNA28S5	1.238997	0.00904
ITCH-IT1	1.25699	0.039504	FAM167A	2.62727	4.91E-05	BCAP29	1.252407	0.000786
ANKRD37	1.257116	0.015462	TMEM158	2.63177	3.48E-05	AKR1B15	1.253106	0.00041
DHFR	1.257881	0.006151	RRAD	2.635387	4.91E-05	HSD3B7	1.253478	0.00254
RRM2	1.259678	0.000892	PRKCH	2.638081	0.00017	CTSZ	1.255244	0.002018
MOGAT3	1.261677	0.001346	CCL26	2.685209	0.000322	CEACAM7	1.256556	0.00393
APOA4	1.264001	0.002515	CCND1	2.774811	6.87E-06	PNMA2	1.257647	0.000475
CXCL1	1.26432	0.000156	FOSL1	2.805619	6.18E-05	POLD4	1.25937	0.048557
ENPP3	1.265291	0.000176	KRT81	2.892108	2.32E-05	DUSP18	1.265999	0.002018
NASP	1.267225	0.000474	COL17A1	2.894151	2.63E-05	PDCD4	1.267323	0.000794
FOSB	1.270334	0.000466	PLAU	2.896152	0.000227	PLS1	1.268359	0.007026
GGTLC2	1.270623	0.000284	ADM	2.919737	3.48E-05	CDC42EP2	1.26913	0.02145
STAP2	1.271946	0.003336	COL7A1	2.930695	0.000164	ABTB1	1.269783	0.001819
HCP5	1.27265	0.001103	SPANXA1	2.976875	0.000345	ZMYM6	1.271991	0.001401
CKAP2L	1.274015	0.000776	UCN2	3.000625	3.48E-05	HEY2	1.274918	0.001035
SPC25	1.274304	0.000506	LPXN	3.004885	6.78E-05	C4orf19	1.279189	0.001722
CCL14	1.275774	0.001522	KRT7	3.183556	0.00035	CSF1	1.279367	0.011239
ASGR1	1.276336	0.000267	SLC1A7	3.213282	0.000215	RFX5	1.280971	0.00134
SAA2	1.283687	0.024827	GFPT2	3.225667	1.63E-05	MTMR11	1.281865	0.00043
DPYSL2	1.284092	0.001204	PFKFB4	3.347126	2.63E-05	LIMK2	1.282123	0.000649
OIP5	1.284229	0.00174	CYP1A1	3.354119	4.49E-05	TIMP4	1.284081	0.001033
PFKFB4	1.288274	0.000392	RASD1	3.359194	5.44E-05	PARP14	1.287801	0.000492
SRPX	1.289733	0.000264	SPANXD	3.429027	2.35E-05	GJB2	1.292906	0.000767
ZC3H12A	1.292849	0.000819	MB	3.52162	1.61E-05	PIK3AP1	1.296005	0.013228
EGR1	1.293092	0.000366	STX1A	3.617332	5.53E-05	TOR1B	1.306868	0.00134
GAMT	1.29334	0.000216	ANXA10	3.845267	1.42E-05	CAB39	1.308621	0.000214
ASPM	1.298663	0.002605	SERPINE1	3.935365	3.35E-05	SDPR	1.31066	0.00112
RND1	1.302938	0.000661	RN7SK	4.17603	6.00E-05	FBXO34	1.31223	0.000888
GAGE12H	1.306219	0.0006	EMP1	4.290851	1.94E-06	ANXA13	1.313394	0.037246
NDN	1.307508	0.000251	PLAUR	4.422429	2.51E-06	PLA2G4C	1.320854	0.001159
KNG1	1.311241	0.000345	RNA5S9	4.470341	8.49E-05	IL32	1.325806	0.000476
TFF1	1.311613	0.011664	CEMIP	4.474591	3.48E-05	SLC16A10	1.325966	0.000262
POLA2	1.313987	0.001386				GRN	1.332445	0.002792
BAG4	1.31475	0.002				OAF	1.342804	0.001814
ASF1B	1.316736	0.000934				GPRC5B	1.348696	0.007817

ETNPPL	1.325112	0.004564
SLC2A2	1.325703	0.000166
SNAPC4	1.327069	0.006563
DPYSL4	1.332495	0.005276
TCEA3	1.332536	0.000169
HMOX1	1.33499	0.000362
CENPE	1.33554	0.000165
SLC22A18/	1.339847	0.001174
ABCA1	1.340786	0.000765
FAM83D	1.347715	0.000187
CEP295	1.347932	0.00132
PAGE1	1.350947	0.003061
ESPL1	1.355045	0.001874
SSX2	1.356024	0.003507
CYP4F3	1.36094	0.000167
KIFC1	1.361585	0.001126
NAT8B	1.36552	0.001996
GAL3ST1	1.366577	0.0003
LRRC75B	1.3674	0.000123
RAB3B	1.367885	0.000413
SEMA6C	1.368237	0.000166
RAD51AP1	1.376241	0.000267
TMEM74B	1.383238	0.000147
PROX1	1.384884	0.003202
TMEM141	1.387718	0.000203
CALB1	1.389248	0.002967
ACOT11	1.391317	0.002335
CYP4F11	1.392639	0.000684
KYNU	1.394369	0.001126
TROAP	1.394714	0.001261
AHSG	1.395426	0.001566
TMPRSS2	1.401165	0.000142
NREP	1.403244	0.001824
IRX3	1.404952	0.000187
VSNL1	1.404953	0.000196
BBOX1	1.406301	0.000558
ACOX2	1.40766	0.000168
PTTG3P	1.411638	0.000513
CDK11A	1.419088	0.002703
APOC2	1.420435	0.001302
TACC3	1.427155	7.58E-05
NCAPD2	1.430957	0.000145
NDC80	1.432575	0.000397
MCM10	1.433735	0.000216
PODXL2	1.44452	0.000379
HILPDA	1.444881	0.000237
GAGE12C	1.446276	0.000203
ADAM23	1.446441	9.00E-05
CGN	1.452815	0.000509
REEP6	1.475258	0.000289
MIS18A	1.4857	0.000237
CCK	1.486352	0.002031
RAB17	1.486768	0.000211
CDCA2	1.487218	0.000142
VCAM1	1.493743	0.000675
LGALS2	1.495536	0.00349
OVOL2	1.500555	0.000368
GAGE12I	1.502256	0.000148
BTN3A2	1.361227	0.00144
SNORD13	1.362141	0.029584
HLA-E	1.36225	0.002907
TIPARP	1.364784	0.002018
RNF19B	1.365936	0.002719
STX11	1.373464	0.000649
IL27RA	1.381145	0.007432
CLDN12	1.382359	0.000304
PLA1A	1.389182	0.001981
PTGR1	1.390257	0.000664
LOC10041	1.391243	0.002763
PTGER4	1.394658	0.000314
TGDS	1.398395	0.000951
SH3BP1	1.4002	0.001016
RELB	1.402409	0.005057
SAMD4A	1.413266	0.002194
IFIT2	1.414726	0.000162
CXCL1	1.418263	0.000193
MYB	1.418413	0.000221
HIST1H2BI	1.43093	0.000324
TRPV2	1.434144	0.001875
CYP2J2	1.435788	0.000759
UGT8	1.440149	0.000556
PELI2	1.444099	0.005301
MGLL	1.44539	0.004447
SLC10A4	1.44609	0.003108
PLA2G12B	1.464037	0.00019
C2CD4B	1.465176	0.00295
NAT1	1.473351	0.000794
CMBL	1.481721	0.000825
ADM	1.48215	0.000875
RDH10	1.487224	0.001157
TMEM163	1.495136	0.001173
RNF148	1.49726	0.000324
RASGRP3	1.500206	0.001457
IGSF3	1.504116	0.00033
STAP2	1.508804	0.000938
HLA-DMA	1.512002	0.000372
TNFRSF11	1.513332	0.000221
HMOX2	1.52545	0.001701
MIR302C	1.536507	0.008886
LRRC31	1.537037	0.000154
IDNK	1.542878	0.000671
ADGRG7	1.54513	0.001981
GPX2	1.547406	0.0006
ICAM2	1.555674	0.001235
LRIG3	1.556632	0.000313
ATP9B	1.557688	0.002343
TXNRD1	1.562386	0.000901
KCTD13	1.565333	0.000664
RPL10A	1.574049	0.009706
PHLDA1	1.580571	6.24E-05
B2M	1.583498	0.006519
LRG1	1.589407	0.001401
SPRY2	1.592816	0.000506
CCL20	1.600861	0.000189
SLC29A3	1.607381	0.001007
MXD4	1.609046	0.000888

CCL3L3	1.5032	0.000762
EVA1C	1.504412	7.26E-05
E2F2	1.510777	0.000491
NAT2	1.513783	0.000907
PLOD2	1.515874	0.000176
HJURP	1.520365	0.000178
CDC25C	1.523415	0.001335
MEGF6	1.526283	0.000127
SNRNP25	1.527098	0.000176
LRRC14	1.533931	0.000433
SUSD2	1.535247	0.000125
SEPW1	1.551881	0.000127
LRP3	1.555742	0.000372
CDA	1.570177	0.000199
TMEM158	1.570972	0.000309
SOS1	1.578272	0.000486
CFI	1.579896	0.003906
NCCRP1	1.586606	0.000158
NOSTRIN	1.587845	0.000147
IQGAP2	1.589782	0.000389
NR1H4	1.592992	0.002914
ZFP36	1.595946	0.000123
SNAR-A1	1.606024	0.000264
AKR1C3	1.607467	0.000203
KRT222	1.608833	5.90E-05
TGM3	1.612261	0.000264
NFKBIZ	1.612526	4.36E-05
CDKN1A	1.628783	0.000187
PDZK1	1.647758	0.000107
COL4A1	1.675679	0.000132
KIAA0101	1.675914	0.000423
GLYCTK	1.683669	7.84E-05
KIF20A	1.684916	7.58E-05
CLDN7	1.686311	0.000142
HOXA13	1.699163	0.000267
OTC	1.706129	0.000251
VIPR1	1.709875	6.65E-05
AHNAK2	1.727476	0.000589
CPE	1.727892	4.79E-05
LINC00261	1.763945	0.00014
EEF1A2	1.7668	0.000279
ITLN2	1.768002	4.59E-05
TUBA4A	1.781911	7.48E-05
TSKU	1.786788	0.000257
RAB11FIP4	1.792663	0.0004
BACE2	1.794777	0.001264
PRODH2	1.816158	7.58E-05
CDX2	1.823401	0.000407
SH3RF2	1.831192	3.37E-05
F12	1.837458	0.000368
ORM2	1.842159	0.003475
PPP1R3C	1.853493	8.34E-05
CEMIP	1.861482	3.96E-05
CA12	1.868323	7.30E-05
CAV2	1.868931	0.00012
SLC39A5	1.869557	0.000927
CIDEB	1.882874	0.000182
CYP1A1	1.884047	0.001332

CD55	1.610132	0.006377
EDN1	1.631826	0.000222
ATG14	1.632701	0.000345
ZNF280A	1.634573	0.000574
STEAP1	1.64383	0.000291
NAT2	1.651284	0.000741
JUP	1.654067	0.000109
ANXA1	1.655798	5.79E-05
ANXA4	1.659906	9.86E-05
AKR1B10	1.663629	0.000122
PIGZ	1.669527	0.0025
IRF1	1.670778	0.000649
EREG	1.680343	0.002041
VNN3	1.691684	0.000301
STOML1	1.701727	0.000722
CYP3A5	1.705911	0.000447
RPL21	1.708612	0.001458
RENBP	1.715628	0.000415
FAM101A	1.716884	0.000134
HIST2H2A	1.722572	0.000214
LCN2	1.740706	0.001458
MX1	1.745312	0.001091
SLC43A2	1.754036	0.000269
PHYHIPL	1.758239	0.000649
MYC	1.759368	0.000226
MAFF	1.759818	0.000285
ASRGL1	1.760615	0.000206
DUOXA2	1.770558	0.002699
FUT4	1.773916	0.000867
CD59	1.776156	0.000528
PLAC8	1.776972	0.00235
PRSS3	1.777643	0.000492
NMB	1.781908	0.000185
NFKBIZ	1.786416	8.35E-05
GLIPR1	1.789153	0.000912
SERTAD1	1.791088	9.87E-05
ADPRHL1	1.800091	0.000487
BCL2L11	1.802208	0.000416
VPREB3	1.827067	0.000536
NFKB2	1.828292	0.001236
TPK1	1.834956	0.000193
IDS	1.83965	9.41E-05
CROT	1.8525	0.000649
RUNDC3B	1.864716	0.000263
LAMC2	1.870465	0.002287
F2R	1.905822	9.89E-05
ARL14	1.916617	0.000145
RND1	1.918501	0.000622
CA2	1.91865	0.000134
ACMSD	1.920556	0.00033
ERO1A	1.929025	0.000459
OSBPL6	1.93505	0.000419
COTL1	1.93551	0.002205
TRIM25	1.969835	0.003434
SORD	1.978374	0.001225
HKDC1	1.978628	0.000187
LYPD1	1.980838	0.000528
VNN2	1.985117	9.41E-05

HPX	1.890003	0.000922
GUCY2C	1.912698	0.000187
SLC25A18	1.914762	7.29E-05
UBD	1.927767	0.001392
FOXN4	1.933247	0.000162
SULT2A1	1.958793	0.000125
RDH5	1.966328	5.45E-05
TMEM86B	1.968911	0.000363
ARHGEF16	1.96975	6.50E-05
AKR1B10	1.975202	4.05E-05
GPX3	1.984868	0.000145
HBQ1	2.004406	0.000119
LTB	2.025314	0.000244
KIAA1683	2.033412	0.000927
CYP4F12	2.033918	2.23E-05
EPCAM	2.057929	3.58E-05
SLPI	2.093189	0.002136
PITX1	2.098727	0.000107
BIRC3	2.105047	0.000188
CITED2	2.125057	5.73E-05
BASP1	2.132299	0.000123
ORM1	2.141383	0.00251
HABP2	2.151871	1.84E-05
NINJ2	2.157002	3.37E-05
CYP2J2	2.186256	0.00012
IL17RB	2.195569	9.93E-05
ERICH4	2.198863	3.71E-05
SCGN	2.224194	4.79E-05
ESPN	2.240835	0.000142
SERPINE2	2.245203	3.72E-05
TTR	2.254091	0.000176
LCN15	2.263752	6.86E-05
KCNK1	2.321211	7.30E-05
MSX1	2.341034	0.000194
PTGR1	2.389981	8.03E-05
FAM13A	2.405964	1.54E-05
TMEM37	2.414476	4.05E-05
VIL1	2.419799	2.23E-05
HMGCS2	2.424745	1.54E-05
VCY	2.437502	8.56E-05
PANX2	2.45392	2.35E-05
TXNIP	2.531354	0.000178
TNFAIP8L1	2.543204	9.00E-05
MTTP	2.567587	6.92E-05
HULC	2.580497	0.002907
CCL20	2.596184	1.63E-05
SAA4	2.607629	0.000107
GJB2	2.631135	2.67E-05
KRT20	2.651919	6.50E-05
PLA2G12B	2.663817	1.49E-05
VCX2	2.70669	1.22E-05
TUBAL3	2.721495	3.72E-05
CCR6	2.939928	1.17E-05
SLC7A9	2.999416	1.02E-05
ALB	3.025043	7.91E-05
DDC	3.034282	1.58E-05
VCX	3.160175	1.58E-05
SGK1	3.164424	5.56E-06

GPRC5A	1.986581	0.000664
HMOX1	1.992089	0.001903
TNFAIP3	2.003729	0.016196
FUT2	2.014849	0.001962
C19orf33	2.027332	0.000154
ATP8B1	2.047033	0.000149
ELF3	2.048755	2.81E-05
CD47	2.066732	0.000193
PCSK5	2.075202	0.000609
MAP3K8	2.087831	0.000647
OLR1	2.088679	8.19E-05
TNF	2.104962	2.70E-05
CA3	2.112327	0.005136
MYOF	2.112939	2.41E-05
RAC2	2.116114	0.000218
GBP2	2.1344	4.81E-05
IFI6	2.135599	0.000416
GBP1	2.139527	0.000155
C10orf54	2.14509	0.000328
CXCL8	2.155406	0.000326
RASD1	2.18333	5.57E-05
TNFAIP2	2.185265	0.000742
TRIM15	2.196227	9.41E-05
ASPH	2.225178	0.000108
LTB	2.278816	0.000433
TNIP1	2.308287	4.32E-05
TMEM45B	2.334263	0.000269
SAA2	2.343212	0.001532
CYP1A1	2.353789	0.000163
KRT20	2.359322	2.02E-05
SEMA6A	2.360907	9.51E-05
MAATS1	2.36168	9.98E-05
PCDH20	2.387413	5.42E-05
GCNT3	2.418117	0.000129
SHD	2.423025	0.000332
BATF3	2.425201	0.000154
NPFFR2	2.434658	0.000123
GAD1	2.448255	0.000609
SLC37A1	2.452217	0.000212
HLA-DMB	2.462723	0.000287
UGT1A1	2.470232	0.000145
TMEM27	2.47177	0.000206
VGF	2.487365	0.000345
RARRES3	2.49462	4.65E-05
BIRC3	2.529307	2.33E-05
SPINK13	2.532655	0.000136
S100A3	2.557449	2.08E-05
BIK	2.563263	9.89E-05
IKBKE	2.604495	5.42E-05
SLC30A1	2.632237	9.41E-05
NT5E	2.650073	1.27E-05
TMEM51	2.659991	2.34E-05
RAMP1	2.662074	0.000177
NDRG1	2.707992	7.98E-05
SLC26A3	2.730196	2.42E-05
TFPI2	2.749011	1.56E-05
CDKN1A	2.752093	4.32E-05
MEP1A	2.766077	2.02E-05

VCX3B	3.169894	1.22E-05
CREB3L3	3.289422	3.85E-05
UGT1A1	3.298838	2.23E-05
APOC3	3.373427	1.58E-05
TRIM15	3.418471	1.49E-05
CDH17	3.612492	2.35E-05
LCN2	3.805955	2.23E-05
APOA1	3.976464	1.54E-05
NDRG1	4.023618	2.19E-06
CEACAM6	4.528682	3.81E-06

ZC3H12A	2.771795	5.42E-05
ADH1C	2.780617	1.86E-05
ABCB1	2.792671	1.86E-05
SGK1	2.834366	2.70E-05
CEBPD	2.850171	2.53E-05
HS3ST1	2.866458	1.27E-05
CDX2	2.886622	8.27E-06
PRR15L	3.003963	0.000193
PLAU	3.046644	9.72E-05
LINC0054[illegible]	3.070205	2.70E-05
F2RL1	3.140264	2.53E-05
CXCL10	3.212476	8.27E-06
CDH17	3.271484	1.27E-05
CA4	3.305042	5.42E-05
CD68	3.413283	1.67E-05
CXCL2	3.42249	1.56E-05
CDC42EP[illegible]	3.469705	2.53E-05
ADH1A	3.564092	2.02E-05
SOCS1	3.566701	4.32E-05
ACE2	3.583101	1.27E-05
TAP1	3.72256	2.02E-05
UBD	3.75683	5.42E-05
GUCY2C	4.087151	7.75E-06
CXCL5	4.123515	2.70E-05
PI3	4.225142	7.63E-06
TRIM31	4.320582	1.27E-05
CXCL6	4.707532	8.27E-06
NPY	4.967586	7.63E-06
MUC13	6.28026	5.03E-06
