# Supplementary material for: The Role of Ferroptosis and Cuproptosis in Curcumin against Hepatocellular Carcinoma
Source: Molecules. 2023 Feb 8;28(4):1623. doi: 10.3390/molecules28041623 (PMC9964324; doi:10.3390/molecules28041623)
Supplement: Supplementary file 1 [file molecules-28-01623-s001.zip › Table S2.pdf]

Table S2

gene	log_rank_p
GAGE1	9.85E-08
GPSM2	2.39E-05
OTOG	5.38E-05
DNER	7.28E-05
CT83	8.24E-05
ERVMER61-1	0.000146
GPR115	0.000153
LINC01559	0.000204
TMEM40	0.000208
PCDHA1	0.000275
FGFRL1	0.000334
HOXD9	0.000344
DYNLT1	0.000408
SHISA9	0.000433
SPP1	0.000465
MTFR2	0.000528
BBS7	0.000554
FABP6	0.000556
PRKCQ	0.00059
SESN2	0.000641
PLXNA1	0.000666
LIN28B	0.000671
MAGEA10	0.000672
RP3-522D1.1	0.000678
APOA1	0.000702
HOXC8	0.000795
GRIA2	0.000854
CLIP4	0.00093
SLC16A3	0.000931
ACTR8	0.001032
PAEP	0.001036
CHRNA5	0.001059
XAGE5	0.001085
SKA3	0.001291
ARHGEF5	0.001295
NR0B1	0.0013
ZNF391	0.001345
ATP5J2-PTCD1	0.00138
FAM83D	0.001444
HAPLN1	0.001498
ANXA10	0.001563
RIBC2	0.001565
LMNB2	0.001619
COL22A1	0.001691
NEIL3	0.001696
SH3RF3	0.001776
CES3	0.001787
MAGEA4	0.001828
FUT4	0.001869
ZGRF1	0.001906
RP11-74C13.3	0.001974
PPT2-EGFL8	0.001988
RP11-883G14.4	0.001989
RP11-440G9.1	0.002042
RP11-758M4.4	0.002044

FAM57B	0.002089
RP11-776H12.1	0.002102
OTC	0.002124
CCDC163P	0.002204
N4BP1	0.002229
MCM7	0.002232
CCDC185	0.002266
EIF5A2	0.002273
LINC00491	0.002286
EPO	0.002404
MMP3	0.002408
RP11-1070N10.7	0.002418
FAM99A	0.002424
SNX5	0.002472
SSPN	0.002505
HMGB2	0.002587
RP11-757F18.5	0.002618
NPIPB3	0.00273
ETV4	0.00274
RP11-184E9.1	0.002783
RP11-503C24.4	0.002848
TEX261	0.002962
PPP1R14C	0.002977
TMTC3	0.003063
MIR4435-1HG	0.003064
ZNF43	0.003135
LRP2	0.003142
KLK13	0.003186
TNNI3	0.003243
C11orf21	0.003248
KIF23	0.00327
FSD1L	0.003298
SLC25A19	0.003303
RAD1	0.003369
NBPF1	0.003523
BIRC5	0.003549
SUCO	0.003566
SGPP2	0.003736
RP11-141M1.3	0.003783
NUP43	0.003882
DPH2	0.003916
CDCP1	0.003938
ATP10D	0.003986
CCDC88A	0.004035
RP11-465N4.5	0.004065
TREM2	0.004104
RP11-180C1.1	0.004118
C9orf41	0.004135
SETBP1	0.00417
MAP3K1	0.004248
S100A9	0.004355
GS1-600G8.5	0.004415
ANAPC5	0.004444
ING5	0.004501
CITED2	0.004511
KIAA0319	0.004537
LINC01518	0.004629
CCDC91	0.004694

RTN1	0.004736
SCGB1D2	0.004756
RP11-738E22.3	0.004761
SLC2A1	0.004795
CCAT1	0.004887
UMAD1	0.004972
AC025016.1	0.004977
SLCO6A1	0.00505
CPXM1	0.005057
MFF	0.005061
APCDD1	0.005139
ARHGAP40	0.005155
ATP5S	0.005207
MKKS	0.005233
C11orf84	0.005304
LINC00648	0.005311
NDUFB6	0.005314
CHRND	0.005371
INMT	0.005383
SULT1C4	0.00548
ITGB5	0.005531
FRAS1	0.005559
TUBA1C	0.005562
BATF2	0.005598
SLC6A17	0.005635
BUB1	0.005665
PAPOLA	0.005692
MTRNR2L8	0.005712
HP1BP3	0.005748
TREM1	0.005828
ARHGAP10	0.005849
ACER2	0.005907
TRNP1	0.005941
LINC00348	0.00598
ABHD4	0.005982
TAZ	0.006031
POPDC3	0.006083
DSCC1	0.006085
LINC00402	0.006089
CHRDL1	0.006162
EGLN3	0.006185
CCL14	0.00619
RP11-766F14.2	0.006199
TM4SF19	0.006269
INTS6	0.006283
NRBP2	0.006294
CENPO	0.006366
CDC45	0.006392
4-Sep	0.006424
NUDT12	0.006425
S100P	0.00649
MANEAL	0.006502
LINC00242	0.006514
BPIFB4	0.00653
SUN1	0.006544
ZC3HC1	0.006576
TFAP4	0.006634
WNT3A	0.006714

FHOD3	0.00673
DNM1L	0.006904
MTRR	0.007025
GNPDA2	0.007053
CLSTN3	0.007086
RP11-421M1.8	0.007138
RP11-317N12.1	0.007184
ORC1	0.007221
ACSL6	0.007235
EPB41L1	0.007289
SOHLH2	0.007329
PABPC4L	0.007355
PPFIA4	0.007407
CDX2	0.00744
SAP30	0.007519
MT1G	0.007565
SMC4	0.007586
MAGEA3	0.007626
RASGRP2	0.007632
AF230666.2	0.007648
AC092580.4	0.007655
RP11-383J24.1	0.007671
ATP6AP1L	0.00768
MT2A	0.007714
WAC-AS1	0.007715
CTD-2006C1.2	0.00774
RP11-968O1.5	0.007744
C7orf43	0.007757
B4GALNT2	0.007796
SYT6	0.00781
IRX5	0.007813
DRD1	0.007893
FAM230C	0.007919
PQLC2L	0.008016
RP11-772C9.1	0.008022
PMAIP1	0.008041
TDRD5	0.008054
PARD3	0.008077
HOXD10	0.008111
AP004372.1	0.008136
LINC00661	0.008168
DGAT2	0.008198
TMEM246	0.008217
AP2S1	0.008228
CDCA8	0.008279
PDZD4	0.008379
ACOT12	0.008389
FBXW11	0.00839
TTC28	0.008418
WDR77	0.008464
ALPI	0.008479
SULT2A1	0.00862
RP11-440D17.4	0.008666
C3orf14	0.008672
COASY	0.008738
BRINP3	0.008764
LGSN	0.008801
TRAPPC13	0.008904

HPD	0.008905
MYH9	0.008937
ZBTB7B	0.00898
RALGAPB	0.008995
LRRC39	0.009023
CXCL8	0.009026
SLC15A1	0.009058
LINC01224	0.009063
P4HA1	0.009153
NAP1L6	0.009155
NCDN	0.009298
NUCB1	0.009525
SP110	0.009554
ADAM12	0.009578
HCN1	0.009595
CTD-2529O21.1	0.009625
RP11-1103G16.1	0.009711
RP11-213H15.1	0.009753
ART5	0.009788
SOX17	0.009803
LINC00942	0.009809
DNAJC6	0.009809
SLC6A1	0.009813
SRM	0.009841
TECR	0.009851
ANKMY2	0.009929
SUPT20H	0.009992
HSP90AB1	0.010051
RP3-412A9.16	0.010061
ARID4A	0.01019
VPS45	0.010286
CHODL	0.010364
C3orf52	0.010479
CH507-513H4.5	0.010541
DCAF8L1	0.010541
RP11-76C10.5	0.010577
ACAP3	0.010635
MAP2K1	0.010683
STK26	0.010692
MAGEA11	0.010765
SLC22A1	0.010823
CTB-129O4.1	0.010837
RP11-587P21.2	0.010859
DTYMK	0.010929
MTRNR2L1	0.010958
RP11-38L15.8	0.010958
RAP1GDS1	0.011112
NCKAP5L	0.011164
CARD6	0.01118
CTD-2171N6.1	0.011198
KIF2C	0.011262
LIMS2	0.011273
LNPEP	0.011295
SBK3	0.011385
CHL1	0.011472
GLI3	0.011506
FAM133A	0.011533
GLS	0.011545

RP11-214O1.2	0.011553
ZNF502	0.011559
NAA38	0.011629
TMEM55A	0.011643
KDELC1	0.011655
LHX4-AS1	0.011892
CRISP3	0.011908
BTAF1	0.011958
IDH2	0.011971
FAM166A	0.011971
SEC61A1	0.011981
OAS1	0.012017
SYN1	0.012063
H2AFY	0.012075
CTD-3064M3.3	0.012165
STK39	0.012168
BTN2A1	0.012168
GRAMD3	0.01223
LINC01139	0.012244
SLC23A1	0.012249
RP11-649A16.1	0.012344
S100A2	0.012363
SERPINF2	0.012435
PPP6R3	0.012442
TOB1	0.012487
LPIN1	0.012514
TGFA	0.012533
FEN1	0.012553
GDF10	0.012591
HNRNPU	0.012611
RP11-145A3.1	0.012671
ZNF10	0.012674
PRSS16	0.01268
CHRAC1	0.012777
PILRA	0.01278
TMEM182	0.012818
WRN	0.012859
PADI3	0.012936
MAP1B	0.01298
RAB11B	0.012995
HLA-DOB	0.013116
RP11-115N4.1	0.013162
ETAA1	0.013163
CTC-459F4.9	0.013211
TMEM44	0.013247
CTC-558O2.1	0.013332
ARMCX1	0.013539
KIF25-AS1	0.013579
PAGR1	0.013636
LAMB1	0.013682
HMCN1	0.013733
DYNC1LI1	0.013757
MURC	0.013805
GABRA2	0.013944
KIFC1	0.013946
NDUFAF5	0.013957
SP8	0.013958
CCNA2	0.013961

TTC13	0.014138
TUBG1	0.014199
SHC4	0.014212
CLIC5	0.014266
NSD1	0.014299
REG4	0.014314
CYP19A1	0.01446
UBE2Q2	0.014466
ENOPH1	0.014521
NEO1	0.014564
RP11-944C7.1	0.014592
DENND1B	0.014676
CREBZF	0.014821
LINC01269	0.014845
GMEB1	0.014875
AC006946.16	0.015012
PSMF1	0.015033
FBXO39	0.015035
RP11-101E14.3	0.015065
TKT	0.015066
ATP6V1H	0.015098
IVNS1ABP	0.015151
RP11-268J15.5	0.015312
BPIFB1	0.015355
MRPL34	0.015372
CNKSR2	0.015413
C15orf40	0.015441
PHB	0.015445
DERL1	0.015449
ZNF714	0.015473
KHNYN	0.015517
PPAP2C	0.015552
RP11-479G22.8	0.015584
LINC01508	0.01561
ZNF225	0.015774
LUZP2	0.015824
POC5	0.015851
PRKCH	0.015863
CENPI	0.015874
CMB9-22P13.1	0.01589
NCAPG	0.015896
RP4-777L9.2	0.01592
NFU1	0.016009
BARD1	0.016111
TPRXL	0.016131
RP11-103J17.2	0.016184
UGT2B10	0.016294
WISP2	0.016339
GABRA3	0.016345
MMP16	0.016352
TTC33	0.016371
SFRP2	0.016478
SMPX	0.01648
CKS2	0.016551
SULF2	0.016575
E2F3	0.016611
TK1	0.016657
HTR2B	0.016684

CADM2	0.01683
HIF1A	0.016959
AAK1	0.016962
CFHR4	0.016982
DNAJC27	0.016994
ZUFSP	0.017036
MAP6	0.017042
PRSS8	0.017083
SFMBT1	0.017116
MEDAG	0.017139
LINC00893	0.017169
LINC00668	0.017208
LINC01234	0.017214
RP11-286N22.8	0.017288
RP3-446N13.5	0.017301
TMEM221	0.017302
IBSP	0.017337
DEGS1	0.017345
11-Sep	0.017399
RP11-614F17.2	0.017485
ZWINT	0.017485
ACSL3	0.017486
CLEC2L	0.017496
LOXL3	0.017508
SF3B5	0.017543
CCPG1	0.01756
RNF19B	0.01756
RPRM	0.017636
WDR20	0.017636
C14orf142	0.017656
ANXA5	0.017757
TBC1D32	0.017775
AC009410.1	0.017904
IFT81	0.01791
LINC01226	0.017979
COL2A1	0.018002
CLEC3B	0.018098
RP11-736K20.5	0.018198
NPEPPS	0.018294
TTK	0.018303
INTS5	0.018308
RNF17	0.018358
MTIF2	0.018368
OSTN-AS1	0.018429
TMEM220-AS1	0.018574
FEM1A	0.01858
RBM19	0.018611
ZDHHC15	0.018635
PSMA7	0.018698
GREB1	0.018744
C17orf49	0.018759
SLC5A9	0.018975
LCMT1	0.018985
RP11-15A1.7	0.019015
ENAM	0.019112
CCNL2	0.019142
ECT2	0.019174
MAFF	0.019234

A1BG	0.019258
NUDC	0.019293
GPATCH3	0.019303
RP11-214F16.8	0.019338
LINC01370	0.019353
ZNF335	0.019438
RP11-619A14.3	0.019495
CIC	0.019538
HMGXB4	0.019604
SLC22A7	0.019707
PHF20	0.019741
NT5DC4	0.019742
ZNF526	0.019774
PNCK	0.019833
TRAPPC1	0.019877
CTD-2538C1.2	0.019885
TBL1X	0.019904
CTC-435M10.12	0.020045
CENPK	0.020048
FAM60A	0.020053
GPD2	0.020105
AOC2	0.020113
CXCL1	0.020131
TMEM175	0.020151
RP11-379H18.1	0.020152
PCDHB8	0.020165
BEST4	0.020228
RP11-452L6.5	0.020248
EIF5A	0.02025
SLC22A25	0.020286
MEA1	0.020335
MAGEA6	0.020345
SULT1B1	0.020374
DACT2	0.02038
FUT2	0.020385
RP11-400D2.2	0.020422
NAPRT	0.020426
PIF1	0.020435
SLC25A38	0.020435
UGT1A8	0.020449
TMEM120A	0.020512
KCND2	0.020527
ATP6V1C2	0.020566
NCR3LG1	0.020588
TEX30	0.020605
VLDLR-AS1	0.020616
GPN3	0.020689
ANKRD13B	0.020786
CDC42BPA	0.020864
CTAG2	0.020927
MAPK14	0.020955
RP11-588H23.3	0.020967
S100A6	0.020968
RP11-320G10.1	0.021036
ZNF575	0.021072
CARD10	0.021178
PAPOLG	0.021262
RP11-284G10.1	0.021409

KRTCAP2	0.021423
CDC26	0.021543
IGFBP3	0.021544
ARHGAP32	0.021566
SPG11	0.021581
ANGPT2	0.021594
SLC22A8	0.021641
TMEM132D	0.021715
GNS	0.02185
GOLGA4	0.021902
GHET1	0.021989
LINC00160	0.022018
ADAMTS5	0.022039
CDC40	0.0222
CTNNA2	0.022205
MXI1	0.022244
RP11-1109F11.3	0.022267
KRT13	0.022343
MFHAS1	0.022371
CIDEC	0.022412
PASK	0.022476
PITX2	0.022492
BCL2L12	0.022502
CHMP4B	0.022556
RP11-1038A11.3	0.02259
RP11-802E16.3	0.022653
TINAG	0.02267
AC005537.2	0.022728
SLC48A1	0.022919
CFAP70	0.022929
TXNRD1	0.022967
ERAL1	0.022997
RP11-932O9.10	0.023026
BRD8	0.023055
PCDHB2	0.02307
ZNF28	0.023129
SLC6A13	0.023148
TFF1	0.023292
BRF2	0.023301
PLAU	0.023364
PFKFB4	0.023442
PRPF4B	0.023488
ANKRD52	0.023501
RP11-864I4.1	0.023573
DNAJC5	0.023615
LINGO4	0.02362
ATOH7	0.023631
ERGIC2	0.02366
ARMCX4	0.02374
RP11-1070N10.3	0.023748
AC009506.1	0.02382
PDF	0.023823
GDNF-AS1	0.023903
KCNF1	0.023906
GABRG2	0.023934
LINC00954	0.02397
KLHL29	0.024104
CASC9	0.024142

IGF2	0.024237
MYO9B	0.024255
RP11-359I18.5	0.024299
TPX2	0.024492
AP001437.1	0.024544
TRAPPC2B	0.024547
VNN2	0.024633
ATP6V0D2	0.024641
RP11-158K1.3	0.024664
PAGE1	0.024702
CHGA	0.024746
BPIFA2	0.024897
RPRD1A	0.025015
METTL5	0.025043
FOXN3	0.025066
SLC26A1	0.025078
CCDC15	0.025099
SPC25	0.025104
HNF4G	0.025169
LRRN4CL	0.025222
RP11-28F1.2	0.025321
PPP1R16A	0.025335
WLS	0.025339
AC245100.1	0.025368
C1orf106	0.025382
B3GNT5	0.02545
KCNQ3	0.02546
CXADR	0.025597
GATAD1	0.025686
PI4KA	0.02569
CENPM	0.025898
CDK5R1	0.025914
CFLAR	0.025922
FBXO28	0.025975
LMO7	0.025998
SPOP	0.026019
ZBTB17	0.026036
JAK2	0.02611
ATG2A	0.02612
LINC00702	0.02619
BTD	0.026258
CDC20	0.026261
MESP2	0.026337
CD1A	0.026346
CTB-33O18.3	0.026348
RP11-442O1.3	0.026384
RANBP3L	0.026553
PLN	0.026774
CYSRT1	0.026778
SIRT2	0.026786
CORIN	0.027077
HIST3H2BB	0.027143
MAGEA8	0.027154
KIF1A	0.027179
RP11-903H12.5	0.027207
HIRA	0.027238
CCND2	0.027311
MAST3	0.027339

MESDC2	0.02734
SCRN1	0.027401
PRSS22	0.02748
EBF3	0.02749
CTD-2147F2.1	0.027497
LPP	0.027606
RER1	0.027606
STK11IP	0.02769
SDF2L1	0.027705
EPHB1	0.027746
SPATS2	0.027856
FDXACB1	0.027864
SLC38A3	0.027977
MAB21L3	0.028016
KCNK2	0.028045
IL1B	0.028088
C11orf85	0.028128
AC007292.3	0.028137
PYCR1	0.028153
WIPF2	0.028341
EFHB	0.028378
FCHO1	0.028419
ZXDC	0.028441
KB-1460A1.1	0.028591
CCT5	0.02861
PPM1A	0.028679
IL20RB	0.028685
C9orf147	0.028707
ROR2	0.02873
ARPC2	0.028769
C2orf72	0.028807
SGMS1-AS1	0.028811
MRPS18B	0.028815
PXYLP1	0.02884
CFHR3	0.028922
LGALS9C	0.02893
TMEM151A	0.028985
KLHL7	0.029083
CDC25B	0.029095
C16orf58	0.029205
MAP3K9	0.029215
HSPA9	0.029242
CCDC51	0.029246
NCF4	0.02926
BYSL	0.029268
TMEM62	0.029319
SLC22A3	0.029405
PSMC5	0.029445
FNDC4	0.029489
MSH2	0.0295
ATP13A1	0.029521
RAB42	0.029615
RRNAD1	0.029636
IBTK	0.029638
CDAN1	0.029699
PICK1	0.029728
PCDHB5	0.029821
XXbac-BPG299F13.1[illegible]	0.029847

NLRP14	0.029851
LINC01126	0.029892
LYAR	0.029921
MAPK6	0.029942
CHST4	0.030076
MIS18A	0.030086
HBB	0.030096
TIMMDC1	0.030121
IFT172	0.030133
OXER1	0.030154
NSUN2	0.030263
IPO9	0.030334
B3GAT3	0.030355
ADAM11	0.03036
SYTL2	0.030369
WDR62	0.030454
AGER	0.030542
AP001626.2	0.030569
ESYT1	0.03061
PLA2G6	0.030632
XPO6	0.030687
FOXP2	0.030833
MMP24	0.030946
F7	0.030979
RP1-239B22.5	0.030995
FRG1	0.031001
G3BP1	0.031134
MCM3	0.03116
CYP2E1	0.031203
CAPN9	0.031208
MIR100HG	0.031216
LEFTY1	0.031282
ATP6V0B	0.031284
KRT17	0.031315
RP11-274H2.5	0.031328
RP11-142A22.4	0.031331
IMPACT	0.031364
CD300LG	0.031429
HEXA	0.031455
LINC01587	0.031457
MNS1	0.031497
SNRPD1	0.031564
TM7SF3	0.031587
CERS1	0.031687
NCBP2	0.03173
NEK4	0.031746
SF1	0.031811
ZNF783	0.031839
FBLN7	0.031905
LSM7	0.031952
VPS26A	0.032044
SLC34A2	0.032149
MAGEB1	0.032184
TTLL1	0.032192
PTPRE	0.032211
EIF2D	0.032269
CDC42EP1	0.032309
RP11-161I6.2	0.032356

EMC4	0.032391
SCARB2	0.032393
CDC7	0.032446
C14orf159	0.032478
HMGA2	0.03248
RCL1	0.03258
UGT8	0.032591
SRSF9	0.032606
SOAT2	0.032614
STAG3	0.03278
SLC6A15	0.032791
ANKRD50	0.032835
LINC01359	0.032887
ABCB9	0.032911
SEMA6A	0.032914
APOA5	0.032989
MIAT	0.032994
PAK1	0.033098
STRIP2	0.033164
HACE1	0.033228
CLEC10A	0.03323
GJB3	0.033509
AC006273.5	0.033541
MFAP5	0.033636
CNOT11	0.033642
RP11-109D9.4	0.03365
WHAMM	0.033681
MBNL3	0.033686
CTB-147N14.6	0.03371
ISCA1	0.033718
CDCA4	0.033765
MCOLN1	0.03382
C4orf47	0.033844
RP11-382D12.2	0.033918
ZNF385D	0.033957
DNAJA2	0.033994
LRRC8C	0.034024
RP11-16E12.2	0.034089
ZBTB47	0.034164
CCNJL	0.034244
ZNF727	0.034471
CARM1	0.034507
EXOC2	0.034521
THOC5	0.034532
RP11-57G10.8	0.034567
MGC39584	0.034716
RP11-1012E15.2	0.034764
TFE3	0.034793
STC2	0.034899
ELFN1	0.035002
RP11-105N14.1	0.03508
ZSCAN32	0.035125
ITIH1	0.035333
RP11-153K11.3	0.035396
ST3GAL4-AS1	0.035403
STARD3	0.035561
HSD17B12	0.035565
PPFIBP1	0.035566

TUBA1B	0.035574
CECR1	0.035655
DUXAP8	0.035659
DTD1	0.035756
LL22NC03-N14H11.1	0.035765
PDS5B	0.035778
MYH3	0.035833
ANG	0.035901
HMGA1	0.035925
CPB2	0.035989
ANKH	0.035992
EPN2	0.036129
MIR210HG	0.036138
RP11-96H17.1	0.036173
RP5-1136G13.2	0.036282
IFT88	0.036314
SEC31A	0.03633
CBX1	0.03633
RP5-888M10.2	0.036333
HOXB2	0.036362
IYD	0.036467
C12orf56	0.036503
SIRT4	0.036551
WNK2	0.036586
KAT2B	0.036705
DNAJC15	0.036828
CSMD3	0.03695
IER3	0.03696
ERN1	0.036962
PRKCD	0.037051
FZD7	0.037261
SFPQ	0.037289
CCDC121	0.037311
SLC38A1	0.037405
COX7A2	0.037468
SLC16A10	0.037534
SDC4	0.037621
RAB36	0.037706
UNQ6494	0.037729
SMPD4	0.03777
OSBPL2	0.037819
LINC01348	0.03782
TFDP1	0.037825
SUSD4	0.037955
IFNLR1	0.038039
RP11-669N7.2	0.038152
SEMA3F	0.038173
PLVAP	0.038193
FANCD2	0.038215
RP11-407A16.3	0.038309
WDR75	0.038447
TAF1A	0.038586
HSH2D	0.038598
HHIPL2	0.038698
ESPL1	0.038731
AC098973.2	0.038736
RP11-178L8.4	0.038752
LINC00958	0.038868

RAP2A	0.038911
EMILIN2	0.038911
SFN	0.038981
CACNA1E	0.039056
P3H2	0.039059
LRRC3-AS1	0.039122
RP11-332J15.2	0.039166
GPRIN1	0.03918
RASSF4	0.03924
TNFRSF17	0.039326
ILDR2	0.03934
PREX2	0.039385
TAT-AS1	0.039409
LMAN2L	0.039419
RP11-322E11.5	0.039472
PRDX4	0.039483
C10orf95	0.039518
SRP9	0.039526
RP11-402P6.9	0.039546
MED10	0.039556
TENM4	0.039633
STX7	0.039646
CELSR3	0.039671
OSR1	0.039685
RP11-242C19.2	0.03969
SPATA41	0.039762
TMEM2	0.039824
PEX12	0.039829
TMEM252	0.039847
TOX4	0.039976
RUNDC3B	0.040017
SFR1	0.04006
LATS1	0.040082
RP5-881L22.5	0.040085
ABT1	0.040207
CSRNP3	0.040271
SPRY3	0.0403
ACSM2B	0.04033
KIAA1109	0.040357
PDCD10	0.040403
DOCK11	0.040515
GRIN2D	0.040523
BLCAP	0.040536
RP11-983P16.4	0.040644
USP43	0.040658
TMEM50A	0.040805
BAD	0.041021
RP11-260M2.1	0.041049
FLRT1	0.041079
SSR3	0.041146
DDB2	0.041163
ABHD16A	0.041193
TINCR	0.041247
PDE8B	0.041267
TCF19	0.041294
NEK8	0.041342
ZNF518B	0.041353
NUP214	0.041415

CEACAM5	0.041432
XRN2	0.041437
PLEKHJ1	0.041442
AGXT	0.041475
GPS1	0.041486
SH2D5	0.041526
SPAG5	0.041533
RP11-579D7.4	0.041599
CYP3A4	0.041748
RP11-15L13.4	0.041792
APH1A	0.041816
RRP8	0.041913
ZNF226	0.041963
SMC2	0.04197
PFN2	0.041977
RP11-495P10.5	0.042042
HHLA2	0.042067
RP11-308B16.2	0.042119
RP11-276H19.2	0.042203
AC004862.6	0.042223
CD5L	0.042237
LINC00982	0.042275
CTB-92J24.3	0.042332
USP2	0.042343
FBXO30	0.042388
FGB	0.042531
YWHAZ	0.042567
FUOM	0.042709
UBE2K	0.042725
TGFB2-AS1	0.042752
CTB-92J24.2	0.042808
RFXANK	0.042928
NECAP1	0.04297
UNC13D	0.043012
DKFZP434I0714	0.043013
LAMTOR5	0.043081
TBRG4	0.043149
CNDP2	0.043152
C3orf67	0.043179
RP11-469N6.1	0.043239
POF1B	0.04324
GSTM3	0.043242
ATP5O	0.043248
C17orf85	0.043264
SSX5	0.043271
NRG2	0.043273
RP5-1125A11.6	0.043371
RP11-511B23.2	0.043461
MRAS	0.043474
COLGALT1	0.04352
CCDC136	0.043542
RALY	0.043551
RGS20	0.043591
XX-FW83563B9.5	0.043596
RP11-672L10.6	0.043683
ZNF223	0.043747
CYP2C8	0.043804
DIO3OS	0.043809

TSPAN5	0.043904
RP11-770J1.3	0.04395
ZBTB6	0.043983
GCA	0.04401
RP11-445P17.8	0.044028
MMP17	0.044123
CYP26B1	0.044127
NUP210	0.044181
ABCA3	0.04425
TAX1BP1	0.044256
CEBPG	0.044408
TBC1D7	0.044455
MEF2A	0.044469
RBM38	0.044475
CTC-480C2.1	0.044636
EVI5	0.044682
POSTN	0.044702
KIAA0895L	0.044715
ZNF287	0.044727
HOXB6	0.044736
RP11-800A18.4	0.044758
SCIMP	0.044814
METTL7B	0.044859
RP4-769N13.6	0.044955
IGSF3	0.04498
ATP13A4	0.044986
ACKR3	0.044986
CLDN7	0.045025
RP11-565A3.2	0.045087
SLC35A4	0.045263
DDX53	0.04533
TRMT44	0.04547
HOXC13	0.045496
LINC00997	0.04558
UBA1	0.045589
NBPF10	0.045675
POLR2B	0.045713
SYN2	0.045732
LSM5	0.045781
PPAP2B	0.045796
CAMKMT	0.045807
NLRP11	0.045813
DGKA	0.045848
CA8	0.045921
TBC1D19	0.046044
MYL6	0.046098
HEBP2	0.046156
ERCC6L	0.046244
SMC5	0.046332
KIAA1522	0.046339
ZFY-AS1	0.046387
PIBF1	0.046526
DCAF8L2	0.04654
VEZF1	0.046551
FKBP1A	0.046613
CAND2	0.046615
CLDN10	0.04668
KIAA1841	0.046718

RPL23A	0.046725
NUS1	0.046736
FMN2	0.04676
PHLDB2	0.046977
RP11-379F4.8	0.047018
UQCR10	0.047095
SLC25A15	0.047115
RP11-408B11.2	0.047128
CTC-203F4.2	0.04716
RP11-94A24.1	0.047171
TGFBR3	0.047209
RUNDC1	0.047246
HDAC5	0.047264
TLDC1	0.04727
KIF3A	0.047354
RP4-640H8.2	0.047357
LINC01014	0.047386
RP11-180M15.7	0.047393
CDC25C	0.047402
LINC01352	0.047437
PPARD	0.047489
EPB41L3	0.047491
EBLN2	0.047585
RP11-443B20.1	0.047585
DPH5	0.047633
CDKN2A	0.047683
ACTA2-AS1	0.047717
RP1-167O22.1	0.047724
BAIAP2L1	0.047763
RP5-1112D6.7	0.047879
PPP2R3B	0.047908
GDE1	0.047942
SAR1A	0.048019
SULT1A2	0.048058
ZNF469	0.048062
COLEC12	0.048067
EYA1	0.048158
MRVI1	0.048219
HSPA4	0.048222
CPA5	0.048276
TYW3	0.048344
CECR6	0.048497
UBAP2L	0.048499
RP3-370M22.8	0.04857
OFD1	0.048614
AP000688.14	0.048635
STAU2-AS1	0.048652
CCDC149	0.048657
ZNF169	0.048734
CRYBG3	0.04875
RNF8	0.048782
ZNF559-ZNF177	0.048811
ZNF70	0.048847
TFF2	0.049032
ADH4	0.049039
RP11-347C12.11	0.049172
RTN4IP1	0.049233
SSTR2	0.049309

FETUB	0.04932
TOMM70A	0.049324
TM9SF2	0.049505
ACAD9	0.049571
KATNBL1	0.049585
CYP4F2	0.049663
GOLPH3	0.049735
RP11-284F21.10	0.049821
AC093495.4	0.049918
SEC23B	0.049975
