# Supplementary material for: The Role of Ferroptosis and Cuproptosis in Curcumin against Hepatocellular Carcinoma
Source: Molecules. 2023 Feb 8;28(4):1623. doi: 10.3390/molecules28041623 (PMC9964324; doi:10.3390/molecules28041623)
Supplement: Supplementary file 1 [file molecules-28-01623-s001.zip › Table S3.pdf]

Table S3

log2FC	pct.1	pct.2	p_val_adj	cluster	gene
2.472505	0.51	0.138	0	0	KLRB1
2.381132	0.875	0.288	0	0	CD52
2.320252	0.684	0.197	0	0	LTB
2.300745	0.787	0.185	0	0	TRAC
2.113378	0.634	0.225	0	0	CCL5
2.090396	0.813	0.197	0	0	CD3D
1.978965	0.865	0.405	0	0	S100A4
1.9425	0.686	0.2	0	0	CD69
1.933617	0.848	0.332	0	0	CXCR4
1.832409	0.707	0.181	0	0	CD2
1.790669	0.797	0.313	0	0	HCST
1.771261	0.653	0.181	0	0	TRBC2
1.724429	0.697	0.169	0	0	CD3E
1.644468	0.765	0.485	0	0	ZFP36L2
1.59234	0.894	0.535	0	0	IL32
1.52556	0.782	0.309	0	0	CORO1A
1.485823	0.53	0.173	0	0	GZMA
1.43346	0.994	0.968	0	0	RPS29
1.360265	1	0.982	0	0	B2M
1.334391	0.646	0.429	0	0	TSC22D3
1.330118	0.607	0.225	0	0	CD48
1.301039	0.58	0.258	0	0	DUSP2
1.300244	0.567	0.27	0	0	EVL
1.292484	0.994	0.979	0	0	RPLP2
1.292223	0.82	0.432	0	0	ARHGDIB
1.272827	0.578	0.289	0	0	ANXA1
1.270342	0.642	0.357	0	0	FXYD5
1.265247	0.95	0.871	0	0	RPS10
1.259891	0.998	0.968	0	0	MALAT1
1.248726	0.93	0.683	0	0	BTG1
1.245984	0.994	0.975	0	0	RPL21
1.234537	0.998	0.933	0	0	TMSB4X
1.233744	0.84	0.603	0	0	JUNB
1.22548	0.565	0.244	0	0	RAC2
1.189308	0.757	0.568	0	0	SARAF
1.167528	0.989	0.957	0	0	RPL23A
1.155192	0.993	0.972	0	0	RPS15A
1.128106	0.501	0.198	0	0	ALOX5AP
1.12719	0.998	0.996	0	0	RPS27
1.116736	0.578	0.254	0	0	CD37
1.10568	0.729	0.602	0	0	RPL17
1.102795	0.993	0.977	0	0	RPL34
1.08919	0.51	0.241	0	0	IL2RG
1.077811	0.98	0.93	0	0	RPL31
1.060782	0.641	0.345	0	0	GMFG
1.033673	0.988	0.964	0	0	RPL27A
1.012508	0.985	0.963	0	0	RPS25
1.013153	0.566	0.335	9.72E-288	0	EMP3
1.043926	0.524	0.296	1.31E-285	0	LDHB
1.052311	0.501	0.267	1.97E-274	0	CRIP1
1.059895	0.567	0.366	6.60E-273	0	RARRES3
1.081263	0.519	0.388	3.63E-156	0	CREM
1.098925	0.541	0.449	1.12E-129	0	ID2
1.523763	0.594	0.573	8.40E-97	0	HBB
4.072545	0.971	0.333	0	1	HLA-DRA

3.359065	0.981	0.598	0	1	CD74
3.340151	0.927	0.343	0	1	HLA-DPB1
3.285336	0.934	0.336	0	1	HLA-DRB1
3.269909	0.904	0.309	0	1	HLA-DPA1
3.255703	0.654	0.211	0	1	LYZ
3.21552	0.543	0.146	0	1	C1QA
3.135981	0.521	0.138	0	1	C1QB
3.108699	0.821	0.155	0	1	HLA-DQB1
3.102123	0.821	0.196	0	1	TYROBP
2.986795	0.765	0.113	0	1	AIF1
2.909519	0.65	0.085	0	1	HLA-DRB5
2.84305	0.76	0.117	0	1	HLA-DQA1
2.763952	0.839	0.514	0	1	CST3
2.753111	0.752	0.135	0	1	FCER1G
2.519839	0.67	0.115	0	1	LST1
2.473553	0.683	0.346	0	1	CTSB
2.363507	0.579	0.054	0	1	MS4A6A
2.311734	0.713	0.259	0	1	CTSS
2.273338	0.62	0.12	0	1	CD68
2.271989	0.772	0.179	0	1	HLA-DMA
2.184839	0.519	0.039	0	1	MS4A7
2.177239	0.822	0.448	0	1	NPC2
2.062374	0.621	0.269	0	1	CTSZ
2.00852	0.792	0.509	0	1	PSAP
2.0069	0.894	0.567	0	1	SAT1
1.981334	0.598	0.085	0	1	CD83
1.869447	0.617	0.081	0	1	HLA-DMB
1.781455	0.88	0.56	0	1	GPX1
1.75608	0.571	0.05	0	1	SPI1
1.749997	0.711	0.359	0	1	GRN
1.600431	0.561	0.106	0	1	C1orf162
1.43125	0.523	0.154	0	1	CAPG
1.392258	0.548	0.159	0	1	AP1S2
1.369169	0.923	0.594	0	1	CYBA
1.357418	0.605	0.216	0	1	PYCARD
1.342519	0.822	0.383	0	1	LAPTM5
1.308232	0.518	0.139	0	1	LY96
1.395194	0.721	0.365	7.60E-295	1	RNASET2
1.61934	0.698	0.345	8.58E-292	1	TYMP
1.214407	0.734	0.331	8.86E-291	1	GSTP1
1.292736	0.996	0.984	3.42E-289	1	FTH1
1.70938	0.654	0.321	1.96E-283	1	FCGRT
1.772029	0.999	0.974	4.20E-255	1	FTL
1.239878	0.522	0.194	1.95E-251	1	CTSH
1.133199	0.5	0.185	5.69E-237	1	PPT1
1.123333	0.55	0.201	1.48E-234	1	RGS10
1.276656	0.809	0.571	7.35E-210	1	S100A11
1.233081	0.652	0.332	2.20E-180	1	COTL1
1.399936	0.561	0.265	1.60E-176	1	RGS2
1.307348	0.525	0.256	1.31E-175	1	ASAH1
1.331077	0.509	0.239	3.41E-169	1	MARCKS
1.077215	0.716	0.497	3.19E-166	1	VAMP8
1.012573	0.804	0.548	1.02E-157	1	FOS
1.244132	0.764	0.553	2.18E-151	1	LGALS1
1.074845	0.773	0.555	6.40E-145	1	DUSP1
1.214446	0.673	0.495	1.31E-92	1	NFKBIA
1.401459	0.54	0.372	2.65E-77	1	SOD2
1.671754	0.63	0.526	1.07E-65	1	CTSD

5.207328	0.994	0.042	0	2	PGA5
4.640355	0.975	0.04	0	2	CHGA
4.231186	0.998	0.32	0	2	SNHG25
3.237192	0.892	0.029	0	2	PAGE1
3.11071	0.847	0.033	0	2	TFPI2
2.867251	0.916	0.164	0	2	CD24
2.829485	0.821	0.059	0	2	HES6
2.801936	0.825	0.027	0	2	GAGE2A
2.711307	0.815	0.019	0	2	CTAG2
2.681713	1	0.478	0	2	APOA1
2.678085	0.926	0.289	0	2	AGT
2.667065	0.946	0.253	0	2	ALDH1A1
2.665395	0.939	0.491	0	2	C9orf16
2.633313	0.911	0.346	0	2	CTSA
2.567265	0.88	0.113	0	2	TFF3
2.37585	0.765	0.037	0	2	MACROD2
2.348817	0.754	0.014	0	2	NEUROD1
2.346783	0.796	0.027	0	2	GAGE1
2.329935	0.91	0.381	0	2	HN1
2.301742	0.808	0.125	0	2	C10orf10
2.244022	0.947	0.606	0	2	MIF
2.240764	0.965	0.666	0	2	UQCRH
2.223308	0.749	0.011	0	2	GAGE12H
2.204411	0.835	0.108	0	2	AGR2
2.160746	0.958	0.595	0	2	ATP5G3
2.149212	0.953	0.601	0	2	ATP5J
2.14219	0.846	0.308	0	2	SLC25A39
2.136855	1	0.924	0	2	GAPDH
2.109785	0.78	0.169	0	2	DPH3
2.09286	0.763	0.089	0	2	PKIB
2.068726	1	0.363	0	2	TTR
2.061156	0.876	0.341	0	2	TIMM13
2.014507	0.767	0.148	0	2	SOX4
1.989845	0.773	0.111	0	2	GSTM3
1.977293	0.775	0.117	0	2	G6PD
1.95444	0.793	0.203	0	2	SMIM4
1.942616	0.987	0.68	0	2	CD63
1.940039	0.922	0.517	0	2	NDUFB9
1.920071	0.93	0.553	0	2	CYCS
1.911049	0.878	0.485	0	2	BSG
1.904752	0.919	0.506	0	2	DDT
1.882412	0.905	0.507	0	2	ATP5H
1.87909	0.965	0.62	0	2	ENO1
1.848154	1	0.974	0	2	FTL
1.832669	0.864	0.235	0	2	FABP5
1.832151	0.676	0.068	0	2	SLC2A1
1.828362	0.671	0.038	0	2	OLFML3
1.803027	0.693	0.021	0	2	PAGE5
1.795944	0.774	0.22	0	2	HIST1H1C
1.786358	0.908	0.522	0	2	NDUFS6
1.771543	0.855	0.255	0	2	LGALS3
1.759327	0.873	0.463	0	2	AURKAIP1
1.73429	0.804	0.242	0	2	MDK
1.727473	0.863	0.389	0	2	CYC1
1.722102	0.934	0.652	0	2	UQCR10
1.710988	0.978	0.785	0	2	UQCRB
1.709914	0.862	0.291	0	2	CES1
1.703644	0.941	0.669	0	2	USMG5

1.703192	0.672	0.016	0	2	LY6K
1.690948	0.741	0.183	0	2	SPON2
1.67814	0.928	0.659	0	2	C14orf2
1.668233	0.769	0.282	0	2	SPATS2L
1.667554	0.776	0.272	0	2	APOA1BP
1.666012	0.787	0.288	0	2	LAGE3
1.659939	0.971	0.773	0	2	COX6C
1.6563	0.823	0.382	0	2	TIMM8B
1.634098	0.782	0.298	0	2	MRPS7
1.623514	0.693	0.138	0	2	HMGB3
1.61177	1	0.983	0	2	FTH1
1.601936	0.889	0.541	0	2	ATP5O
1.591863	0.808	0.262	0	2	MARCKSL1
1.586222	0.754	0.21	0	2	SDC2
1.5853	0.684	0.056	0	2	GPX7
1.577673	0.649	0.074	0	2	LINC00261
1.571111	0.951	0.554	0	2	TUBA1B
1.568729	0.969	0.821	0	2	ATP5L
1.563944	0.751	0.262	0	2	MKKS
1.563838	0.952	0.646	0	2	TPI1
1.535438	0.874	0.457	0	2	CHCHD10
1.530848	0.766	0.298	0	2	UQCC2
1.526622	0.836	0.437	0	2	SCAND1
1.510489	0.774	0.299	0	2	BNIP3
1.506421	0.729	0.23	0	2	GPI
1.487594	0.881	0.365	0	2	CLU
1.487005	0.984	0.841	0	2	PPIA
1.484709	0.599	0.031	0	2	FABP3
1.484028	0.882	0.549	0	2	NDUFB4
1.480551	0.761	0.287	0	2	PNKD
1.474294	0.843	0.448	0	2	NDUFA6
1.47209	0.738	0.21	0	2	TSTA3
1.471899	0.95	0.759	0	2	COX7A2
1.471602	0.724	0.105	0	2	RBP1
1.468387	0.633	0.047	0	2	SCGN
1.468226	0.651	0.078	0	2	ING2
1.465753	0.786	0.309	0	2	UQCRC1
1.462032	0.789	0.316	0	2	SNRPN
1.458697	0.818	0.337	0	2	PSMB5
1.450625	0.755	0.223	0	2	SNHG9
1.446147	0.884	0.608	0	2	ATP5D
1.436824	0.593	0.014	0	2	TKTL1
1.424206	0.93	0.722	0	2	POLR2L
1.42311	0.818	0.432	0	2	PGAM1
1.408298	0.795	0.384	0	2	PDCD5
1.407245	0.661	0.122	0	2	RHOBTB3
1.406013	0.816	0.466	0	2	EIF2S2
1.397469	0.856	0.469	0	2	PRDX2
1.397259	0.757	0.308	0	2	POLR2E
1.390835	0.615	0.073	0	2	PLCXD2
1.388767	0.833	0.452	0	2	SLIRP
1.38534	0.581	0.097	0	2	CCDC34
1.377771	0.937	0.638	0	2	ATP5J2
1.377444	0.911	0.674	0	2	COX8A
1.362442	0.833	0.302	0	2	KRT8
1.354026	0.761	0.306	0	2	FAM162A
1.348825	0.904	0.64	0	2	NDUFB2
1.348349	0.866	0.544	0	2	NDUFB7

1.325819	0.532	0.006	0	2	PGA4
1.319708	0.935	0.688	0	2	ALDOA
1.318758	0.947	0.704	0	2	ATP5I
1.317541	0.793	0.387	0	2	NDUFB3
1.313404	0.874	0.536	0	2	PRDX5
1.311174	0.602	0.16	0	2	SEL1L
1.301906	0.739	0.259	0	2	PSMF1
1.300889	0.866	0.52	0	2	ATP5B
1.298144	0.585	0.01	0	2	CSAG1
1.295405	0.876	0.492	0	2	ROMO1
1.290009	0.511	0.01	0	2	ANGPT4
1.284258	0.661	0.164	0	2	CD320
1.28294	0.773	0.313	0	2	PCBD1
1.282732	0.877	0.644	0	2	NDUFS5
1.280524	0.776	0.244	0	2	STMN1
1.280007	0.667	0.127	0	2	PAFAH1B3
1.270781	0.641	0.154	0	2	METTL12
1.265842	0.645	0.073	0	2	NQO1
1.265645	0.683	0.226	0	2	PIGT
1.242439	0.559	0.022	0	2	[illegible]11-525A16.4
1.241748	0.613	0.063	0	2	PCBP4
1.225346	0.943	0.756	0	2	NDUFA4
1.218662	0.89	0.626	0	2	COX7B
1.215961	0.581	0.024	0	2	GTSF1
1.215277	0.69	0.238	0	2	FKBP3
1.2111	0.724	0.267	0	2	MTCH2
1.209022	0.653	0.164	0	2	EXOSC4
1.202962	0.554	0.009	0	2	NR0B1
1.188357	0.713	0.281	0	2	MRPS16
1.183884	0.535	0.011	0	2	PHACTR3
1.17846	0.738	0.292	0	2	CCT5
1.170735	0.57	0.061	0	2	CCDC28B
1.167499	0.531	0.117	0	2	ANGPTL4
1.167351	0.65	0.182	0	2	MRPL15
1.164503	0.57	0.048	0	2	USP18
1.162751	0.91	0.748	0	2	UBL5
1.16243	0.53	0.02	0	2	SLC29A4
1.160479	0.668	0.207	0	2	TUSC2
1.154902	0.598	0.112	0	2	SNHG19
1.148303	0.721	0.259	0	2	PRMT1
1.148111	0.724	0.304	0	2	SLC9A3R1
1.142963	0.568	0.075	0	2	PLA2G12B
1.141678	0.701	0.228	0	2	FAM213A
1.139467	0.807	0.237	0	2	GSTA1
1.137228	0.909	0.657	0	2	NDUFA1
1.136299	0.702	0.233	0	2	NDRG1
1.13579	0.591	0.097	0	2	NDUFAF6
1.131061	0.512	0.013	0	2	CACNA2D1
1.123479	0.652	0.174	0	2	UROD
1.109256	0.953	0.766	0	2	COX6B1
1.10462	0.577	0.082	0	2	RRAGD
1.101621	0.696	0.174	0	2	SERINC2
1.097854	0.745	0.292	0	2	RAB13
1.094595	0.618	0.132	0	2	FAM127B
1.084018	0.629	0.194	0	2	RALA
1.076306	0.664	0.192	0	2	HSF1
1.064128	0.543	0.041	0	2	FRZB
1.064066	0.55	0.031	0	2	GMPR

1.058063	0.676	0.231	0	2	RNF114
1.048403	0.616	0.177	0	2	FAM45A
1.044485	0.588	0.083	0	2	PTP4A3
1.0281	0.597	0.124	0	2	MAD1L1
1.027158	0.543	0.069	0	2	MPP6
1.019064	0.624	0.164	0	2	MRPL17
1.016881	0.819	0.272	0	2	PRAP1
1.015426	0.647	0.22	0	2	SCAN16-AS1
1.01169	0.619	0.177	0	2	TOMM40
1.000575	0.943	0.701	9.02E-298	2	SOD1
1.207136	0.832	0.492	1.01E-297	2	MRPS21
1.028535	0.885	0.503	1.30E-296	2	CTSD
1.147766	0.694	0.275	2.31E-294	2	NUCB2
1.221659	0.793	0.412	1.32E-293	2	TMEM147
1.120457	0.739	0.331	5.92E-293	2	COX20
1.198386	0.76	0.372	1.01E-291	2	TXNDC17
1.201461	0.769	0.359	1.55E-291	2	CACYBP
1.064986	0.707	0.292	3.56E-291	2	MRPL34
1.031594	0.66	0.255	3.64E-288	2	MRPL36
1.2133	0.784	0.406	9.46E-284	2	SNRPD1
1.206284	0.811	0.479	4.45E-281	2	LAMTOR5
1.170502	0.791	0.403	1.63E-279	2	VDAC2
1.223601	0.898	0.674	2.76E-279	2	PPDPF
1.110455	0.853	0.558	6.73E-276	2	NDUFA3
1.031682	0.731	0.311	5.48E-275	2	IDH2
1.009504	0.678	0.268	6.30E-274	2	HIGD1A
1.18481	0.835	0.519	1.58E-272	2	COPE
1.106331	0.904	0.673	2.32E-272	2	GPX4
1.001529	0.666	0.265	1.07E-270	2	COA4
1.155846	0.857	0.538	4.74E-270	2	NDUFC2
1.132056	0.766	0.369	2.96E-267	2	NUDC
1.217848	0.905	0.609	1.70E-265	2	LDHA
1.157085	0.845	0.586	1.08E-264	2	NDUFA11
1.047809	0.778	0.363	2.89E-263	2	ECHS1
1.044279	0.729	0.313	7.87E-261	2	PRDX4
1.147031	0.779	0.413	6.77E-259	2	BANF1
1.102645	0.825	0.487	5.12E-256	2	ATP6V0B
1.197646	0.807	0.445	3.30E-254	2	ATP5C1
1.120516	0.764	0.386	1.96E-252	2	UQCRFS1
1.167556	0.823	0.52	1.84E-249	2	RBM8A
1.054992	0.855	0.557	4.75E-242	2	NDUFB1
1.017321	0.855	0.597	1.29E-240	2	NEDD8
1.058458	0.767	0.43	4.10E-231	2	TCEB1
1.064031	0.812	0.502	1.91E-227	2	SNRPB
1.025873	0.765	0.416	2.03E-223	2	NDUFS8
1.042617	0.824	0.523	5.39E-217	2	COX5A
1.020497	0.785	0.533	8.01E-209	2	MYEOV2
1.004236	0.793	0.493	1.01E-204	2	NDUFB10
1.405549	0.603	0.316	2.04E-147	2	MT-ND6
1.000616	0.995	0.946	5.71E-59	2	MT-ND1
7.389386	0.963	0.037	0	3	NTS
4.309494	0.841	0.148	0	3	IGFBP1
3.602982	0.922	0.248	0	3	FGA
3.534629	0.998	0.593	0	3	SERPINA1
3.49058	0.92	0.269	0	3	FGB
3.455745	0.996	0.638	0	3	ALB
3.422879	0.888	0.181	0	3	TFPI
3.407986	0.929	0.29	0	3	FGG

3.132763	0.843	0.175	0	3	EFNA1
3.128552	0.669	0.032	0	3	SERPINE1
3.104956	0.759	0.1	0	3	IGFBP3
2.972287	0.799	0.135	0	3	CP
2.930035	0.836	0.204	0	3	APOB
2.894718	0.899	0.3	0	3	FN1
2.84679	0.878	0.139	0	3	SPINK1
2.827499	0.91	0.274	0	3	C3
2.696537	0.868	0.202	0	3	FGL1
2.68507	0.655	0.097	0	3	GDF15
2.667951	0.864	0.226	0	3	CFH
2.604577	0.792	0.136	0	3	TM4SF5
2.540923	0.773	0.228	0	3	NDRG1
2.481998	0.775	0.138	0	3	HPN
2.39538	0.855	0.242	0	3	GC
2.353692	0.907	0.335	0	3	TF
2.346706	0.951	0.508	0	3	P4HB
2.256902	0.701	0.122	0	3	AGR2
2.21452	0.804	0.192	0	3	C2
2.189196	0.809	0.213	0	3	C1S
2.148362	0.714	0.041	0	3	VNN1
2.13911	0.679	0.083	0	3	LRG1
2.125477	0.72	0.118	0	3	CFHR1
2.107553	0.665	0.123	0	3	PON1
2.08636	0.86	0.259	0	3	ORM1
2.065467	0.976	0.427	0	3	AMBP
2.030962	0.691	0.089	0	3	UGT2B15
2.001851	0.648	0.071	0	3	MET
1.982364	0.792	0.176	0	3	LGALS4
1.962091	0.525	0.021	0	3	HGF
1.950052	0.696	0.145	0	3	ITIH2
1.916147	0.741	0.219	0	3	A2M
1.907369	0.663	0.042	0	3	VNN2
1.804617	0.737	0.189	0	3	ASPH
1.78253	0.749	0.171	0	3	SERINC2
1.737851	0.728	0.175	0	3	C1R
1.703948	0.754	0.192	0	3	ASGR1
1.70221	0.93	0.325	0	3	MGST1
1.666689	0.588	0.123	0	3	SERPIND1
1.657606	0.714	0.24	0	3	CKS1B
1.638506	0.852	0.469	0	3	CLTA
1.625483	0.535	0.012	0	3	MUC13
1.613149	0.975	0.522	0	3	IFITM3
1.589162	0.737	0.197	0	3	PON2
1.576357	0.665	0.152	0	3	APCS
1.553955	0.823	0.384	0	3	BRI3
1.52893	0.507	0.117	0	3	CAV1
1.526532	0.573	0.072	0	3	TMEM45A
1.507922	0.65	0.159	0	3	ITIH3
1.506092	0.686	0.147	0	3	LAPTM4B
1.481239	0.549	0.071	0	3	CCL20
1.472332	0.869	0.467	0	3	PDIA3
1.460452	0.797	0.366	0	3	CAPZA2
1.441211	0.738	0.226	0	3	CERS2
1.435805	0.686	0.192	0	3	SLC50A1
1.421781	0.51	0.11	0	3	C15orf48
1.41881	0.707	0.224	0	3	SERPINF1
1.407334	0.543	0.047	0	3	ADGRG6

1.38371	0.578	0.092	0	3	PLOD2
1.382251	0.528	0.036	0	3	UGT2B11
1.371754	0.718	0.227	0	3	ANXA4
1.362534	0.695	0.249	0	3	ATP1B1
1.345185	0.573	0.134	0	3	SHC1
1.336554	0.752	0.255	0	3	AKR1C3
1.331215	0.781	0.309	0	3	PRDX4
1.305054	0.569	0.103	0	3	C5
1.287866	0.713	0.198	0	3	PERP
1.234153	0.513	0.056	0	3	C8B
1.23201	0.597	0.176	0	3	NNMT
1.219205	0.741	0.28	0	3	SYPL1
1.204524	0.904	0.295	0	3	VTN
1.166778	0.507	0.04	0	3	AGTR1
1.155265	0.566	0.126	0	3	ADAM15
1.151607	0.609	0.108	0	3	UGT2B4
1.142542	0.683	0.184	0	3	ORM2
1.133562	0.66	0.207	0	3	GGH
1.117349	0.546	0.075	0	3	SMPDL3A
1.072438	0.547	0.151	0	3	CAV2
1.067076	0.52	0.045	0	3	GOLM1
1.066858	0.621	0.202	0	3	GANAB
1.06423	0.527	0.131	0	3	PLOD3
1.060202	0.723	0.176	0	3	AZGP1
1.040283	0.54	0.102	0	3	SERPINA5
1.016261	0.597	0.189	0	3	SCAMP3
1.01101	0.633	0.166	0	3	C8G
1.004529	0.52	0.092	0	3	HABP2
1.542475	0.893	0.538	6.97E-303	3	HSP90B1
1.426062	0.86	0.478	1.06E-300	3	PDIA6
1.640402	0.773	0.37	4.85E-291	3	FDPS
1.264195	0.869	0.463	8.94E-290	3	ITGB1
1.19543	0.646	0.219	1.41E-289	3	S100A16
1.55666	0.633	0.23	2.20E-287	3	MT-ATP8
1.004629	0.629	0.226	7.55E-282	3	SLC39A1
1.633314	0.544	0.165	1.35E-276	3	HPX
1.011502	0.932	0.445	3.52E-267	3	SEPP1
1.045397	0.787	0.314	2.87E-262	3	KRT18
1.127006	0.732	0.331	1.42E-255	3	DPM3
1.137663	0.506	0.146	1.93E-254	3	PLG
1.476803	0.521	0.148	4.46E-251	3	TFF3
1.078471	0.568	0.188	1.02E-246	3	YBX3
1.143773	0.642	0.277	5.95E-241	3	DAP3
1.55911	0.824	0.458	2.69E-239	3	HSPA5
1.008493	0.574	0.206	9.07E-230	3	S100A13
1.283516	0.667	0.292	8.31E-228	3	TMEM141
1.029415	0.655	0.28	1.64E-220	3	LMAN1
1.162192	0.966	0.766	8.42E-218	3	COX6B1
1.176448	0.897	0.61	6.80E-208	3	LDHA
1.261297	0.646	0.259	9.41E-204	3	PTGR1
1.200138	0.681	0.309	1.27E-198	3	BNIP3
1.155968	0.883	0.597	8.56E-198	3	CALR
2.359848	0.996	0.984	3.58E-190	3	MT-CO3
2.197565	0.992	0.951	4.74E-186	3	MT-CYB
1.257809	0.992	0.95	2.01E-184	3	RPS5
1.225946	0.996	0.924	2.55E-184	3	GAPDH
2.167914	0.982	0.943	2.07E-171	3	MT-ND3
1.036026	0.88	0.627	9.74E-167	3	PPIB

1.011998	0.895	0.627	8.70E-157	3	ENO1
1.86116	0.992	0.958	1.23E-128	3	MT-ATP6
1.656424	0.994	0.987	1.54E-125	3	MT-CO2
1.409683	0.905	0.832	1.70E-116	3	MT-ND5
1.382875	0.88	0.691	6.59E-98	3	S100A10
1.401411	0.984	0.947	1.22E-97	3	MT-ND1
1.222543	0.992	0.974	2.09E-97	3	MT-ND4
1.122772	0.569	0.326	4.57E-54	3	NUPR1
4.535611	1	0.277	0	4	APOC3
3.94157	1	0.56	0	4	APOA2
3.869327	0.994	0.275	0	4	RBP4
3.801754	0.967	0.099	0	4	FXYD1
3.71721	0.996	0.375	0	4	TTR
3.566868	0.991	0.297	0	4	VTN
3.427983	0.952	0.086	0	4	COX7A1
3.388346	0.957	0.103	0	4	ALDOB
3.120605	0.776	0.09	0	4	SAA1
2.949215	0.966	0.151	0	4	KNG1
2.835876	0.958	0.213	0	4	TMEM176A
2.702262	0.957	0.247	0	4	TMEM176B
2.616666	0.944	0.235	0	4	IGFBP2
2.611453	0.952	0.172	0	4	ORM2
2.608593	0.907	0.1	0	4	HPD
2.60362	0.996	0.568	0	4	APOE
2.572031	0.974	0.339	0	4	TF
2.549138	0.962	0.347	0	4	BLVRB
2.531812	0.957	0.16	0	4	SERPINC1
2.528138	0.924	0.224	0	4	INSIG1
2.522048	0.95	0.174	0	4	LGALS4
2.496504	0.896	0.087	0	4	FBP1
2.481102	1	0.555	0	4	APOC1
2.475464	0.958	0.271	0	4	PRAP1
2.465301	0.883	0.096	0	4	KHK
2.376543	0.958	0.163	0	4	AHSG
2.356082	0.817	0.013	0	4	CYP2A7
2.341265	0.975	0.334	0	4	CYB5A
2.328629	0.98	0.353	0	4	DCXR
2.279783	0.942	0.168	0	4	AZGP1
2.275109	0.982	0.265	0	4	FABP1
2.259874	0.976	0.26	0	4	ORM1
2.252903	0.997	0.488	0	4	APOA1
2.213487	0.874	0.103	0	4	HGD
2.148033	0.971	0.429	0	4	ATOX1
2.078357	0.921	0.282	0	4	HINT2
2.069057	0.991	0.702	0	4	SOD1
2.02961	0.909	0.249	0	4	HAGH
2.008115	0.74	0.045	0	4	PCK1
1.998316	0.808	0.023	0	4	SLC22A7
1.995996	0.858	0.089	0	4	HMGCS2
1.992872	0.965	0.492	0	4	ROMO1
1.949811	0.778	0.017	0	4	PPP1R1A
1.939161	0.901	0.24	0	4	GSTA1
1.92362	0.844	0.041	0	4	CCL16
1.916038	0.935	0.122	0	4	ADIRF
1.911832	0.88	0.195	0	4	CHPT1
1.910852	0.894	0.274	0	4	GRHPR
1.893608	0.991	0.677	0	4	UQCRQ
1.891054	0.989	0.767	0	4	COX6B1

1.878361	0.827	0.129	0	4	ASS1
1.853715	0.988	0.434	0	4	AMBP
1.829302	0.932	0.214	0	4	HP
1.815497	0.885	0.187	0	4	ANG
1.815244	0.907	0.309	0	4	DNPH1
1.784279	0.888	0.23	0	4	EBP
1.760779	0.863	0.125	0	4	BAAT
1.75034	0.878	0.15	0	4	ITIH3
1.742971	0.839	0.104	0	4	SERPINA6
1.739666	0.946	0.366	0	4	ADI1
1.713768	0.893	0.231	0	4	TMEM14A
1.705782	0.977	0.573	0	4	PEBP1
1.700057	0.772	0.028	0	4	TAT
1.698737	0.84	0.076	0	4	HRG
1.688623	0.867	0.176	0	4	GPX3
1.683823	0.866	0.132	0	4	AGXT
1.682816	0.839	0.154	0	4	CES2
1.68199	0.889	0.247	0	4	NDUFV3
1.676719	0.755	0.119	0	4	SLPI
1.674435	0.9	0.109	0	4	HULC
1.670976	0.814	0.108	0	4	F10
1.668229	0.906	0.32	0	4	CISD3
1.654575	0.963	0.321	0	4	RARRES2
1.647354	0.976	0.657	0	4	NDUFA1
1.645891	0.822	0.091	0	4	GJB1
1.640855	0.949	0.537	0	4	NDUFC2
1.640768	0.934	0.384	0	4	POLR2I
1.614974	0.858	0.178	0	4	QPRT
1.612033	0.906	0.312	0	4	KRT18
1.611648	0.824	0.173	0	4	DHRS4L2
1.608924	0.57	0.037	0	4	SDS
1.606048	0.841	0.192	0	4	GALK1
1.60236	0.945	0.488	0	4	PRDX6
1.600452	0.704	0.021	0	4	CYP8B1
1.593199	0.945	0.289	0	4	IFI27
1.588681	0.782	0.122	0	4	RAMP1
1.584611	0.784	0.05	0	4	SEC14L2
1.584444	0.778	0.078	0	4	AKR7A3
1.584208	0.793	0.059	0	4	SAA4
1.582045	0.884	0.191	0	4	ASGR1
1.581764	0.952	0.45	0	4	MPC2
1.574691	0.978	0.325	0	4	APOH
1.564898	0.83	0.094	0	4	F12
1.56362	0.793	0.104	0	4	PCK2
1.560334	0.935	0.529	0	4	SLC25A5
1.560257	0.856	0.271	0	4	SUCLG1
1.557321	0.797	0.099	0	4	HSD17B6
1.55455	0.995	0.971	0	4	RPS16
1.533292	0.697	0.013	0	4	ZG16
1.529971	0.698	0.014	0	4	TAT-AS1
1.526454	0.91	0.301	0	4	SERPING1
1.519406	0.749	0.035	0	4	CPS1
1.51015	0.72	0.037	0	4	APOA5
1.49998	0.924	0.433	0	4	NUCKS1
1.481685	0.998	0.643	0	4	ALB
1.481494	0.806	0.154	0	4	SDC1
1.47989	0.887	0.193	0	4	ITIH1
1.467748	0.975	0.702	0	4	COX5B

1.459935	0.893	0.326	0	4	HSD17B10
1.451541	0.754	0.08	0	4	SLC2A2
1.447606	0.726	0.069	0	4	HAAO
1.44156	0.971	0.688	0	4	PSMA7
1.42501	0.759	0.138	0	4	GADD45G
1.424059	0.758	0.113	0	4	SLC27A5
1.423987	0.986	0.449	0	4	SEPP1
1.402323	0.817	0.184	0	4	STARD10
1.400926	0.675	0.036	0	4	ACOX2
1.386679	0.849	0.153	0	4	F2
1.384861	0.773	0.095	0	4	FMO5
1.375033	0.961	0.331	0	4	MGST1
1.374844	0.829	0.251	0	4	ACAA1
1.364799	0.861	0.298	0	4	CEBPD
1.359719	0.697	0.057	0	4	CLDN3
1.351354	0.789	0.138	0	4	CYP3A5
1.349018	0.655	0.034	0	4	GPT
1.340135	0.906	0.208	0	4	APOB
1.336938	0.78	0.134	0	4	MAT1A
1.336484	0.843	0.178	0	4	C4BPB
1.323076	0.729	0.123	0	4	TM7SF2
1.309833	0.697	0.053	0	4	ACSM2B
1.302259	0.713	0.059	0	4	ARG1
1.300649	0.844	0.23	0	4	FUOM
1.29788	0.782	0.164	0	4	PPP1R16A
1.283859	0.916	0.461	0	4	KRTCAP2
1.280654	0.805	0.238	0	4	RHOB
1.273175	0.708	0.078	0	4	MACROD1
1.269501	0.733	0.119	0	4	SELENBP1
1.269499	0.747	0.186	0	4	C20orf27
1.267423	0.688	0.09	0	4	FGGY
1.265475	0.705	0.089	0	4	BHMT
1.262644	0.968	0.606	0	4	ATP5J
1.259645	0.96	0.626	0	4	COX7B
1.25768	0.93	0.403	0	4	PTMS
1.251476	0.843	0.246	0	4	MRPL24
1.245547	0.896	0.312	0	4	PCBD1
1.243008	0.849	0.312	0	4	AKR1A1
1.238751	0.927	0.448	0	4	MRPL51
1.23449	0.882	0.364	0	4	NENF
1.233701	0.741	0.109	0	4	DHRS4
1.231659	0.661	0.04	0	4	NR1I3
1.230321	0.878	0.374	0	4	ERGIC3
1.224351	0.649	0.043	0	4	LINC01485
1.217032	0.919	0.465	0	4	NDUFA2
1.2122	0.804	0.116	0	4	APOC2
1.206366	0.852	0.291	0	4	DUSP23
1.201758	0.651	0.038	0	4	HFE2
1.199773	0.865	0.271	0	4	A1BG
1.196755	0.838	0.286	0	4	TMEM205
1.185507	0.823	0.271	0	4	NDUFS2
1.178196	0.762	0.129	0	4	COL18A1
1.170783	0.536	0.091	0	4	GPC3
1.164188	0.957	0.555	0	4	NDUFB1
1.163062	0.77	0.193	0	4	PHYH
1.161575	0.902	0.436	0	4	MRPL41
1.156364	0.884	0.377	0	4	RNF181
1.153658	0.844	0.321	0	4	MRPL14

1.148807	0.808	0.215	0	4	GATM
1.144472	0.94	0.544	0	4	ATP5O
1.144208	0.982	0.83	0	4	HINT1
1.142757	0.645	0.049	0	4	ACSM2A
1.141642	0.677	0.056	0	4	AMN
1.12202	0.746	0.149	0	4	ECHDC2
1.11661	0.849	0.255	0	4	AKR1C3
1.116173	0.675	0.086	0	4	SHMT1
1.113039	0.963	0.697	0	4	COX6A1
1.110347	0.605	0.029	0	4	ASPDH
1.110139	0.867	0.389	0	4	PSMD4
1.103102	0.8	0.268	0	4	FAM50A
1.096632	0.643	0.044	0	4	ETNK2
1.096079	0.985	0.757	0	4	NDUFA4
1.085297	0.847	0.303	0	4	PIN4
1.084344	0.74	0.145	0	4	PROC
1.080419	0.889	0.398	0	4	LAMTOR2
1.079923	0.952	0.676	0	4	EIF3K
1.073719	0.905	0.384	0	4	PDCD5
1.072233	0.651	0.051	0	4	CDHR5
1.071368	0.725	0.127	0	4	YBEY
1.06294	0.662	0.098	0	4	PEMT
1.062819	0.628	0.047	0	4	FTCD
1.050771	0.751	0.203	0	4	COPRS
1.049266	0.808	0.146	0	4	HPN
1.045413	0.778	0.147	0	4	TM4SF5
1.037758	0.821	0.243	0	4	TM4SF4
1.035322	0.707	0.147	0	4	HMGB3
1.033756	0.551	0.008	0	4	XPNPEP2
1.032147	0.84	0.338	0	4	EIF6
1.027752	0.678	0.137	0	4	SLC22A18
1.027415	0.588	0.06	0	4	GPR88
1.024277	0.616	0.048	0	4	AR
1.022524	0.616	0.089	0	4	CEBPA
1.020536	0.601	0.057	0	4	CYP2C9
1.019966	0.696	0.143	0	4	TCEA3
1.015407	0.805	0.31	0	4	YIF1A
1.015273	0.802	0.155	0	4	CD9
1.013637	0.901	0.306	0	4	NUPR1
1.012985	0.626	0.094	0	4	ACADSB
1.010126	0.601	0.101	0	4	TMEM97
1.00634	0.662	0.077	0	4	TFR2
1.003544	0.699	0.127	0	4	PON3
1.050735	0.928	0.452	5.23E-300	4	SLIRP
1.099186	0.934	0.492	8.15E-297	4	COX17
1.022728	0.86	0.364	5.46E-293	4	ECHS1
1.054671	0.947	0.55	6.65E-289	4	NDUFB4
1.033725	0.965	0.562	2.03E-282	4	GPX1
1.025916	0.938	0.538	4.05E-278	4	PRDX5
1.040111	0.902	0.449	2.27E-277	4	ATP5G1
1.019848	0.874	0.413	3.56E-277	4	TMEM147
1.004014	0.878	0.401	2.61E-274	4	SCP2
1.083932	0.961	0.673	4.11E-273	4	GPX4
1.080776	0.909	0.483	2.47E-266	4	FKBP2
1.062892	0.87	0.43	3.00E-264	4	ECH1
1.01027	0.917	0.651	2.23E-171	4	NEAT1
1.263002	0.582	0.375	1.08E-30	4	HIST1H4C
2.964829	0.932	0.446	0	5	JUND

2.455327	0.685	0.099	0	5	METRNL
2.401253	0.972	0.36	0	5	PTPRC
2.226436	0.828	0.218	0	5	ARL4C
2.159687	0.746	0.226	0	5	NKG7
2.068786	0.918	0.347	0	5	IRF1
2.055408	0.822	0.25	0	5	ZNF331
2.015141	0.855	0.271	0	5	REL
1.984526	0.733	0.207	0	5	PIK3R1
1.859631	0.706	0.153	0	5	GZMK
1.842429	0.885	0.246	0	5	LCP1
1.804806	0.71	0.155	0	5	TMEM2
1.803846	0.992	0.737	0	5	BTG1
1.795857	0.56	0.089	0	5	PRF1
1.788272	0.754	0.204	0	5	SYNE2
1.751844	0.868	0.371	0	5	RNF213
1.744677	0.87	0.395	0	5	CREM
1.744458	0.825	0.214	0	5	CST7
1.727536	0.984	0.715	0	5	DDX5
1.688981	0.923	0.545	0	5	NCL
1.686417	0.811	0.309	0	5	EZR
1.667677	0.757	0.133	0	5	RUNX3
1.666608	0.881	0.234	0	5	GZMA
1.662331	0.706	0.088	0	5	SYTL3
1.633015	0.956	0.446	0	5	CXCR4
1.625848	0.856	0.316	0	5	DUSP2
1.621746	0.975	0.696	0	5	CALM1
1.617614	0.704	0.091	0	5	RNF125
1.604809	0.835	0.308	0	5	CCL5
1.567171	0.738	0.223	0	5	RORA
1.55959	0.942	0.515	0	5	SRSF7
1.540746	0.552	0.089	0	5	GBP5
1.538691	0.908	0.436	0	5	PRRC2C
1.469358	0.803	0.297	0	5	EML4
1.466112	0.931	0.551	0	5	SRSF5
1.465462	0.706	0.176	0	5	CNOT6L
1.451732	0.762	0.173	0	5	FYN
1.426628	0.985	0.541	0	5	SRGN
1.388861	0.682	0.122	0	5	AKNA
1.386627	0.776	0.177	0	5	ETS1
1.384585	0.547	0.065	0	5	IL2RB
1.370161	0.862	0.28	0	5	HLA-F
1.365509	0.691	0.144	0	5	CTSW
1.363032	0.814	0.303	0	5	ELF1
1.357579	0.645	0.188	0	5	RNF19A
1.355922	0.508	0.11	0	5	MAP3K8
1.352749	0.547	0.126	0	5	TIPARP
1.350363	0.698	0.214	0	5	SMCHD1
1.330081	0.679	0.179	0	5	ABHD17A
1.312254	0.838	0.24	0	5	STK17B
1.303816	0.704	0.168	0	5	PDE4B
1.294355	0.693	0.159	0	5	APOBEC3G
1.28321	0.689	0.161	0	5	TNFRSF1B
1.28213	0.785	0.284	0	5	PDCD4
1.280424	0.618	0.09	0	5	IKZF3
1.263012	0.633	0.133	0	5	RAB8B
1.258133	0.685	0.187	0	5	CD3G
1.246802	0.767	0.269	0	5	ARID4B
1.245664	0.692	0.153	0	5	FAM46C

1.237444	0.862	0.281	0	5	IL2RG
1.228359	0.564	0.081	0	5	ITGA4
1.223112	0.998	0.888	0	5	HLA-A
1.191518	0.586	0.157	0	5	BOD1L1
1.190585	0.585	0.134	0	5	DDX3Y
1.187427	0.576	0.127	0	5	VPS37B
1.183875	0.628	0.105	0	5	PTPN22
1.177524	0.865	0.298	0	5	CD48
1.173603	0.993	0.855	0	5	HLA-C
1.168512	0.638	0.118	0	5	SKAP1
1.156938	0.681	0.177	0	5	GPR65
1.138334	0.661	0.186	0	5	ADGRE5
1.119517	0.663	0.155	0	5	GZMM
1.118495	0.569	0.118	0	5	SLFN5
1.096251	0.589	0.078	0	5	STAT4
1.092876	0.642	0.143	0	5	DOCK8
1.092693	0.557	0.089	0	5	ARAP2
1.083139	0.531	0.088	0	5	ARHGAP9
1.075979	0.584	0.117	0	5	CCND2
1.073357	0.669	0.181	0	5	APBB1IP
1.068027	0.611	0.125	0	5	CD247
1.067778	0.555	0.085	0	5	P11-138A9.2
1.049821	0.527	0.105	0	5	ZBTB1
1.04293	0.515	0.09	0	5	LINC-PINT
1.015192	0.586	0.106	0	5	CD96
1.014818	0.532	0.084	0	5	SH2D2A
1.008582	0.61	0.141	0	5	PTGER4
1.005552	0.623	0.138	0	5	EMB
1.292527	0.786	0.3	4.05E-303	5	AKAP13
1.279717	0.7	0.234	1.17E-301	5	ANKRD11
1.202028	0.521	0.108	1.10E-300	5	GZMH
1.385193	0.878	0.465	1.16E-300	5	GNAS
1.163872	0.715	0.208	1.91E-300	5	SAMSN1
1.048871	0.654	0.194	4.97E-299	5	DDX6
1.161827	0.62	0.17	5.40E-299	5	KIAA1551
1.096021	0.662	0.202	1.41E-296	5	USP15
1.298199	0.906	0.494	1.62E-295	5	7-Sep
1.261479	0.577	0.145	1.72E-293	5	SLA
1.054831	0.574	0.145	1.47E-291	5	BTN3A2
1.263356	0.69	0.208	9.64E-291	5	SLC2A3
1.137338	0.635	0.188	2.28E-290	5	CHD1
1.186906	0.857	0.295	2.52E-290	5	CD2
1.301153	0.762	0.26	1.51E-286	5	TNFAIP3
1.071898	0.845	0.283	2.80E-283	5	CD3E
1.463154	0.818	0.315	5.98E-282	5	NR4A2
1.460325	0.829	0.379	1.49E-281	5	ANKRD12
1.269376	0.845	0.4	4.90E-280	5	HNRNPU
1.002667	0.575	0.156	2.06E-278	5	VPS13C
1.126989	0.663	0.216	4.27E-278	5	MACF1
1.246859	0.72	0.264	1.84E-277	5	G3BP2
1.119322	0.607	0.184	5.09E-277	5	DNTTIP2
1.273255	0.869	0.425	2.25E-276	5	DDX24
1.065401	0.806	0.267	2.25E-273	5	FYB
2.313634	0.718	0.252	2.97E-273	5	CCL4
1.13771	0.733	0.275	3.96E-272	5	TLN1
1.13006	0.601	0.18	5.49E-272	5	KMT2A
1.064699	0.599	0.176	4.19E-266	5	UTRN
1.049563	0.857	0.303	8.77E-266	5	CD69

1.105285	0.683	0.208	1.71E-265	5	CYTIP
1.218332	0.639	0.22	2.88E-264	5	SMAP2
1.131185	0.649	0.215	5.52E-263	5	MYCBP2
1.212657	0.832	0.41	6.07E-261	5	SRRM2
1.171237	0.979	0.691	9.26E-261	5	HLA-E
1.069369	0.661	0.194	4.87E-260	5	CLEC2D
1.057441	0.614	0.197	9.48E-260	5	SMARCA5
1.040503	0.68	0.226	5.67E-254	5	TAP1
1.132338	0.778	0.318	3.99E-250	5	CD81
1.092899	0.767	0.311	2.26E-249	5	MSN
1.04134	0.76	0.294	1.93E-248	5	STK4
1.218829	0.832	0.414	2.54E-248	5	SFPQ
1.179067	0.653	0.238	6.58E-248	5	BPTF
1.083584	0.609	0.202	3.36E-246	5	IQGAP2
1.117996	0.8	0.353	2.37E-240	5	KMT2E
1.148635	0.675	0.215	4.48E-240	5	KLRB1
1.146755	0.793	0.37	2.48E-238	5	JAK1
1.286014	0.804	0.37	9.58E-238	5	YPEL5
1.04182	0.694	0.261	2.10E-237	5	STAT3
1.095366	0.656	0.236	1.23E-236	5	GCC2
1.051953	0.698	0.272	1.16E-234	5	BCLAF1
1.251483	0.613	0.219	5.52E-233	5	GOLGB1
1.083515	0.751	0.302	9.22E-232	5	PPP2R5C
1.199235	0.769	0.351	4.03E-231	5	DDX3X
1.303889	0.785	0.375	5.59E-231	5	FAM177A1
1.036136	0.706	0.281	5.31E-229	5	DHX36
1.072737	0.524	0.152	8.03E-226	5	DUSP5
1.010376	0.562	0.182	2.12E-225	5	BDP1
1.066173	0.506	0.14	2.24E-223	5	ZEB2
1.319874	0.919	0.618	1.64E-222	5	IL32
1.022939	0.912	0.537	3.09E-221	5	RBM39
1.087814	0.632	0.227	8.64E-220	5	UBE2S
1.004471	0.913	0.538	3.84E-215	5	YWHAZ
1.077031	0.633	0.201	1.96E-211	5	TRBC1
1.048799	0.705	0.303	7.18E-211	5	HP1BP3
1.078763	0.686	0.298	1.11E-210	5	CDV3
1.357692	0.674	0.284	8.88E-203	5	STAT1
1.041554	0.721	0.291	1.87E-201	5	ISG20
1.066968	0.817	0.397	1.13E-200	5	RARRES3
1.080206	0.662	0.246	1.43E-199	5	CLEC2B
1.027924	0.892	0.512	8.89E-198	5	PNRC1
1.021609	0.685	0.293	7.16E-197	5	ATRX
1.07478	0.84	0.41	1.94E-194	5	DDIT4
1.117257	0.855	0.464	6.26E-194	5	HSPA5
1.078737	0.665	0.247	4.08E-192	5	CD7
1.272455	0.779	0.343	2.09E-187	5	ANXA1
1.210983	0.888	0.543	4.39E-186	5	ZFP36L2
1.044235	0.955	0.733	2.35E-172	5	HSP90AB1
1.026274	0.588	0.305	2.14E-102	5	TUBA4A
1.009657	0.538	0.283	6.23E-93	5	CMC1
1.055773	0.757	0.496	1.93E-88	5	NFKBIA
3.895827	0.651	0.028	0	6	RBP7
3.707923	0.709	0.014	0	6	VWF
3.581886	0.861	0.028	0	6	RAMP2
3.453116	0.851	0.05	0	6	HSPG2
3.428825	0.93	0.109	0	6	IGFBP7
3.420276	0.865	0.102	0	6	GNG11
3.415827	0.743	0.013	0	6	PLVAP

3.362076	0.64	0.006	0	6	CLDN5
3.342262	0.855	0.076	0	6	SPARC
3.168076	0.848	0.095	0	6	EGFL7
3.142599	0.807	0.056	0	6	ENG
3.09279	0.748	0.087	0	6	ID1
2.973124	0.647	0.057	0	6	SPARCL1
2.959929	0.823	0.097	0	6	ID3
2.93862	0.732	0.182	0	6	SLC9A3R2
2.900713	0.659	0.006	0	6	RAMP3
2.84175	0.648	0.103	0	6	PLPP1
2.786052	0.812	0.191	0	6	TM4SF1
2.754735	0.767	0.15	0	6	GSN
2.751858	0.814	0.104	0	6	CRIP2
2.686376	0.956	0.297	0	6	IFI27
2.671999	0.771	0.075	0	6	TCF4
2.660475	0.578	0.042	0	6	MGP
2.605256	0.651	0.005	0	6	CLEC14A
2.59034	0.678	0.028	0	6	COL4A1
2.531054	0.743	0.071	0	6	PECAM1
2.505792	0.662	0.074	0	6	TIMP3
2.4742	0.595	0.017	0	6	AQP1
2.456783	0.926	0.494	0	6	FKBP1A
2.449944	0.809	0.204	0	6	IGFBP4
2.406782	0.598	0.145	0	6	INSR
2.305655	0.747	0.089	0	6	PTRF
2.279904	0.703	0.053	0	6	NPDC1
2.275565	0.666	0.023	0	6	ESAM
2.264459	0.58	0.021	0	6	FLT1
2.263469	0.668	0.1	0	6	EPAS1
2.245964	0.837	0.343	0	6	CD59
2.24394	0.675	0.004	0	6	ECSCR
2.221874	0.643	0.01	0	6	CALCRL
2.216663	0.731	0.144	0	6	TGFBR2
2.211335	0.643	0.003	0	6	EMCN
2.195182	0.647	0.102	0	6	HYAL2
2.176069	0.652	0.004	0	6	ADGRL4
2.171989	0.642	0.015	0	6	LDB2
2.16794	0.601	0.004	0	6	PCAT19
2.159606	0.61	0.042	0	6	COL4A2
2.137016	0.752	0.215	0	6	APP
2.136783	0.627	0.038	0	6	CD93
2.09147	0.57	0.013	0	6	PODXL
2.063731	0.794	0.231	0	6	A2M
2.057357	0.85	0.365	0	6	VAMP5
2.038438	0.594	0.004	0	6	CD34
2.037487	0.815	0.146	0	6	RNASE1
2.028819	0.68	0.118	0	6	CAV1
1.984965	0.522	0.065	0	6	SPRY1
1.969008	0.575	0.01	0	6	NOTCH4
1.951186	0.556	0.028	0	6	EMP1
1.950698	0.849	0.379	0	6	CCDC85B
1.943621	0.651	0.099	0	6	ARHGAP29
1.907934	0.52	0.017	0	6	TMEM88
1.888946	0.972	0.535	0	6	IFITM3
1.780039	0.678	0.108	0	6	MEF2C
1.750358	0.504	0.004	0	6	PTPRB
1.744949	0.551	0.087	0	6	LPAR6
1.724512	0.539	0.003	0	6	CDH5

1.714926	0.517	0.031	0	6	TMEM204
1.693273	0.59	0.117	0	6	NFIB
1.669279	0.507	0.005	0	6	MMRN2
1.650623	0.557	0.098	0	6	BCAM
1.629429	0.625	0.173	0	6	CNN3
1.594178	0.568	0.145	0	6	SEC14L1
1.566634	0.581	0.143	0	6	ICAM2
1.528015	0.547	0.11	0	6	PRKCDBP
1.488511	0.509	0.043	0	6	TINAGL1
1.469523	0.575	0.13	0	6	SERPINH1
1.60687	0.669	0.212	1.25E-300	6	SPTBN1
1.585238	0.697	0.228	4.48E-299	6	S100A16
1.863727	0.618	0.174	8.14E-292	6	CD9
1.590921	0.658	0.25	2.12E-250	6	RDX
1.89733	0.556	0.176	7.42E-245	6	PRCP
1.645245	0.692	0.284	1.36E-242	6	STOM
1.567354	0.601	0.208	9.37E-240	6	IL6ST
1.564444	0.58	0.184	3.71E-239	6	TSC22D1
1.724747	0.879	0.523	1.52E-238	6	VIM
1.726495	0.77	0.384	3.05E-230	6	SEPW1
1.538938	0.697	0.286	4.44E-225	6	MARCKSL1
1.53015	0.777	0.416	1.30E-224	6	RHOC
1.591051	0.788	0.413	5.96E-221	6	BST2
1.094087	0.999	0.951	2.38E-211	6	TMSB10
1.241236	0.509	0.166	5.84E-193	6	ETS2
1.19409	0.67	0.271	1.23E-188	6	NGFRAP1
1.186058	0.89	0.585	4.57E-188	6	IFITM2
1.325651	0.729	0.325	6.84E-186	6	TXNIP
1.2099	0.586	0.195	2.56E-185	6	GIMAP7
1.756511	0.553	0.195	7.31E-184	6	HES1
1.147536	0.578	0.216	6.93E-178	6	S100A13
1.146534	0.561	0.199	2.29E-176	6	YBX3
1.2761	0.609	0.257	4.04E-171	6	MGST2
1.169279	0.693	0.331	4.84E-165	6	FCGRT
1.163051	0.903	0.762	1.07E-164	6	ITM2B
1.014628	0.526	0.185	6.68E-161	6	WBP5
1.612723	0.591	0.255	2.11E-156	6	CTNNB1
1.19315	0.794	0.506	6.87E-153	6	ANXA2
1.250071	0.542	0.221	3.27E-152	6	PDLIM1
1.009568	0.574	0.21	2.80E-151	6	GIMAP4
1.416748	0.581	0.246	1.05E-150	6	MARCKS
1.038361	0.619	0.272	1.34E-150	6	CD151
1.157272	0.646	0.312	1.57E-147	6	RAB13
1.151235	0.789	0.48	4.33E-146	6	ITGB1
1.104179	0.902	0.698	6.32E-144	6	HLA-E
1.136464	0.901	0.645	6.88E-142	6	HSPB1
1.16208	0.716	0.41	5.83E-141	6	YWHAE
1.21711	0.588	0.258	7.83E-140	6	RHOB
1.293257	0.537	0.235	2.73E-136	6	OAZ2
1.185349	0.508	0.213	1.86E-132	6	TACC1
1.168882	0.764	0.533	7.43E-132	6	SNX3
1.101304	0.663	0.328	5.91E-130	6	CD81
1.067572	0.54	0.246	3.62E-126	6	SERPINB6
1.072953	0.67	0.38	8.04E-124	6	CDC37
1.251733	0.686	0.406	2.17E-114	6	LMNA
1.004502	0.516	0.24	3.89E-114	6	LEPROT
1.019982	0.686	0.425	1.65E-99	6	NAA38
1.103579	0.898	0.808	1.53E-92	6	SRP14

1.042727	0.546	0.276	1.05E-86	6	IFI6
1.390942	0.557	0.29	1.10E-75	6	RGCC
2.410955	0.505	0.272	2.04E-72	6	MTRNR2L8
1.062838	0.664	0.452	4.59E-63	6	IER2
1.201588	0.692	0.565	6.20E-48	6	FOS
2.463134	0.513	0.356	5.05E-38	6	MTRNR2L12
4.71476	0.938	0.332	0	7	GLUL
4.151913	0.862	0.077	0	7	CYP2E1
3.750154	0.916	0.12	0	7	HULC
3.617971	0.692	0.024	0	7	CYP3A4
3.33213	0.952	0.336	0	7	APOH
3.297507	0.924	0.366	0	7	DCXR
2.881405	0.809	0.196	0	7	AKR1C1
2.710488	0.61	0.033	0	7	HSD11B1
2.708117	0.938	0.347	0	7	CYB5A
2.512841	0.996	0.563	0	7	APOC1
2.487531	0.774	0.275	0	7	CBR1
2.438879	0.962	0.621	0	7	TXN
2.399891	0.724	0.067	0	7	ALDH1L1
2.319725	0.857	0.259	0	7	PTGR1
2.31831	0.725	0.123	0	7	HPD
2.292429	0.783	0.255	0	7	IGFBP2
2.280977	0.683	0.125	0	7	GPX2
2.237353	0.869	0.181	0	7	AHSG
2.236187	0.691	0.066	0	7	ANGPTL3
2.231549	0.693	0.143	0	7	SORD
2.201929	0.787	0.309	0	7	STRA13
2.176756	0.812	0.313	0	7	CES1
2.166639	0.574	0.084	0	7	ADH1B
2.083791	0.664	0.111	0	7	AKR1C2
2.082707	0.509	0.043	0	7	LINC00844
2.066962	0.679	0.167	0	7	APCS
2.025262	0.647	0.118	0	7	AKR1B10
2.002431	0.711	0.146	0	7	BAAT
1.978716	0.591	0.042	0	7	AQP9
1.969534	0.847	0.282	0	7	ALDH1A1
1.95769	0.894	0.345	0	7	MGST1
1.937154	0.769	0.234	0	7	HP
1.883403	0.788	0.182	0	7	SERPINC1
1.857447	1	0.567	0	7	APOA2
1.844455	0.936	0.581	0	7	PEBP1
1.834469	0.604	0.144	0	7	ASPSCR1
1.81193	0.679	0.15	0	7	MAT1A
1.624838	0.549	0.061	0	7	ADH1C
1.623366	0.575	0.123	0	7	PAH
1.585416	0.595	0.099	0	7	C4BPA
1.582583	0.608	0.138	0	7	CPB2
1.57719	0.642	0.129	0	7	HGD
1.497835	0.544	0.064	0	7	FMO3
1.478867	0.568	0.126	0	7	APOM
1.314855	0.501	0.061	0	7	MASP2
1.176996	0.819	0.274	0	7	SPP1
1.162551	0.894	0.292	0	7	RBP4
1.455804	0.587	0.155	2.50E-297	7	PCYT2
1.669219	0.882	0.462	8.86E-295	7	MPC2
1.718827	0.954	0.445	4.90E-294	7	AMBP
1.293635	0.535	0.12	1.01E-292	7	FMO5
1.746542	0.81	0.291	1.77E-292	7	PRAP1

1.552136	0.948	0.46	3.15E-291	7	SEPP1
1.299609	1	0.975	1.06E-277	7	FTL
1.817044	0.695	0.231	7.37E-276	7	GATM
1.806254	0.87	0.573	2.85E-275	7	TBCA
1.581701	0.533	0.127	3.87E-275	7	UGT2B4
2.302274	0.647	0.21	6.45E-273	7	SCD
1.726759	0.816	0.383	1.13E-266	7	ADI1
1.7971	0.728	0.294	1.23E-262	7	GRHPR
1.426745	0.954	0.684	1.86E-262	7	UQCRQ
1.337672	0.526	0.132	3.13E-259	7	HIBADH
1.390626	0.58	0.153	5.65E-259	7	PLG
1.776861	0.892	0.601	4.02E-256	7	DBI
1.417474	0.634	0.2	2.00E-254	7	C4BPB
1.697562	0.822	0.433	2.88E-253	7	ETFB
1.340546	0.575	0.17	5.46E-253	7	CRLS1
1.384567	0.588	0.178	3.63E-251	7	CYP27A1
2.319963	0.773	0.288	5.60E-247	7	FABP1
1.457083	0.568	0.145	5.31E-246	7	CFHR1
1.385811	0.575	0.168	7.77E-245	7	HRSP12
1.348137	0.77	0.269	1.32E-243	7	TMEM176B
1.427489	0.814	0.339	3.32E-241	7	RARRES2
1.347153	0.623	0.205	1.23E-240	7	STARD10
1.625021	0.709	0.266	1.54E-240	7	MDK
1.535042	0.939	0.709	1.63E-239	7	SOD1
3.522	0.6	0.218	6.87E-238	7	MT1E
1.609961	0.657	0.238	1.00E-237	7	ALDH2
1.178384	0.726	0.238	2.58E-233	7	TMEM176A
1.755809	0.691	0.26	1.70E-232	7	EPHX1
1.435638	0.561	0.181	7.48E-225	7	GCSH
1.141158	0.955	0.834	1.37E-220	7	HINT1
1.269337	0.611	0.202	1.56E-218	7	QPRT
1.221834	0.738	0.26	4.02E-216	7	GSTA1
1.486715	0.678	0.269	4.12E-216	7	ACAA1
1.208394	0.508	0.142	1.56E-211	7	SELENBP1
1.372775	0.631	0.198	1.85E-211	7	AZGP1
1.648737	0.562	0.195	2.90E-211	7	C7orf55
1.655859	0.614	0.223	5.32E-203	7	GGH
1.327822	0.846	0.58	1.07E-201	7	SHFM1
1.253475	0.655	0.251	1.56E-198	7	FUOM
1.262146	0.531	0.166	1.95E-198	7	PROC
1.451551	0.586	0.222	4.59E-198	7	TIMM17A
1.562557	0.789	0.503	4.62E-197	7	ANAPC11
1.320651	0.728	0.331	7.60E-197	7	PCBD1
1.496643	0.576	0.215	3.47E-193	7	POR
1.340175	0.666	0.28	4.08E-193	7	MPST
1.237477	0.715	0.319	1.02E-185	7	BNIP3
1.148802	0.539	0.181	2.43E-181	7	PXMP2
1.255334	0.603	0.234	2.55E-181	7	ECI2
1.258648	0.606	0.22	3.46E-177	7	ITIH1
1.080781	0.553	0.186	1.42E-174	7	PPP1R16A
1.21269	0.8	0.447	7.09E-172	7	ATOX1
1.27838	0.885	0.637	9.23E-170	7	PRDX1
1.539063	0.703	0.35	4.29E-167	7	TKT
1.234629	0.67	0.275	3.42E-158	7	AKR1C3
1.026307	0.52	0.184	1.19E-157	7	MSRB1
3.605155	0.643	0.349	2.70E-154	7	MT1X
1.208011	0.607	0.283	2.59E-149	7	MRPS33
1.152944	0.562	0.219	1.75E-148	7	CAT

1.048422	0.874	0.692	1.40E-144	7	HSPE1
1.03847	0.718	0.326	2.10E-141	7	KRT8
1.148127	0.773	0.459	3.18E-138	7	GSTO1
1.138272	0.803	0.503	2.35E-136	7	PRDX6
1.056066	0.545	0.228	7.90E-134	7	RAB4A
1.14318	0.8	0.526	1.51E-133	7	DDT
1.154044	0.647	0.33	4.85E-133	7	COA3
1.121088	0.723	0.433	1.41E-130	7	MDH2
1.07978	0.638	0.332	1.17E-129	7	MRPL55
1.144814	0.58	0.25	6.83E-129	7	HIST1H1C
3.185431	0.765	0.573	6.29E-127	7	MT2A
1.190762	0.676	0.361	8.94E-127	7	PSMB5
1.208535	0.704	0.401	5.03E-124	7	TALDO1
1.059768	0.569	0.257	5.77E-123	7	ADH5
1.166482	0.624	0.315	2.28E-122	7	PRDX3
1.051375	0.551	0.245	7.62E-114	7	ACAT1
1.060802	0.602	0.294	1.62E-113	7	MRPL12
1.164931	0.686	0.388	1.01E-111	7	FDPS
1.118272	0.638	0.37	3.38E-111	7	C7orf73
1.051643	0.52	0.219	2.22E-110	7	ANG
1.088655	0.615	0.311	2.72E-110	7	DECR1
1.060751	0.733	0.48	4.27E-110	7	SNRPE
1.019773	0.621	0.319	6.27E-109	7	NME1
1.14585	0.683	0.456	8.19E-100	7	MRPL41
1.008365	0.682	0.382	2.74E-97	7	ECHS1
1.474479	0.746	0.525	7.13E-80	7	SQSTM1
3.073652	0.6	0.048	0	8	KRT19
3.047186	0.972	0.667	0	8	S100A6
3.034025	0.686	0.113	0	8	DEFB1
2.935404	0.518	0.017	0	8	FXYD2
2.784296	0.561	0.013	0	8	KRT7
2.714126	0.719	0.135	0	8	SLPI
2.593065	0.799	0.241	0	8	ANXA4
2.577104	0.584	0.05	0	8	PDZK1IP1
2.44148	0.571	0.026	0	8	S100A14
2.309489	0.867	0.328	0	8	KRT18
2.286617	0.854	0.323	0	8	KRT8
2.082925	0.569	0.087	0	8	ELF3
2.004742	0.621	0.093	0	8	SPINT2
1.919177	0.567	0.09	0	8	TESC
1.763699	0.504	0.099	2.29E-294	8	ABCC3
2.821997	0.74	0.264	1.45E-274	8	ATP1B1
2.796027	0.738	0.282	6.48E-267	8	SPP1
1.637683	0.682	0.203	2.62E-263	8	TM4SF1
1.52844	0.57	0.135	1.09E-256	8	GPX2
1.450374	0.704	0.205	2.84E-247	8	CD24
1.949646	0.906	0.578	2.31E-235	8	S100A11
2.181415	0.706	0.262	6.92E-235	8	TM4SF4
1.919195	0.681	0.24	1.88E-230	8	C12orf75
1.637774	0.629	0.222	1.62E-206	8	PERP
1.535224	0.606	0.238	8.03E-162	8	S100A16
1.30451	0.916	0.647	6.23E-156	8	HSPB1
1.333892	0.8	0.481	3.92E-155	8	DSTN
1.399888	0.634	0.274	8.69E-155	8	CD151
1.390614	0.707	0.354	3.53E-153	8	CD59
1.285915	0.574	0.22	8.53E-148	8	S100A13
1.22588	0.904	0.771	9.27E-144	8	CALM2
1.750429	0.525	0.184	1.77E-139	8	SPINK1

1.206121	0.796	0.482	1.43E-136	8	ITGB1
1.242287	0.719	0.367	5.45E-129	8	TIMP1
1.352155	0.878	0.731	3.59E-127	8	TCEB2
1.279552	0.709	0.423	7.04E-123	8	VDAC1
1.455356	0.761	0.51	3.29E-121	8	ANXA2
1.830601	0.709	0.395	5.26E-119	8	CLU
1.392908	0.853	0.699	8.24E-117	8	S100A10
1.628072	0.888	0.659	9.16E-115	8	NEAT1
1.303743	0.755	0.48	6.53E-114	8	LAPTM4A
1.082505	0.541	0.223	4.12E-112	8	SPTBN1
1.421677	0.638	0.343	6.33E-107	8	ATP1A1
1.588282	0.609	0.333	1.19E-98	8	HSD17B11
1.20079	0.726	0.481	1.72E-94	8	TMED2
1.0239	0.574	0.261	1.40E-93	8	NBEAL1
1.12733	0.57	0.282	3.72E-92	8	TPM1
1.092545	0.605	0.31	5.73E-92	8	SPATS2L
1.213972	0.689	0.43	8.62E-92	8	CANX
1.056066	0.829	0.546	1.45E-89	8	IFITM3
1.18196	0.603	0.313	2.58E-89	8	C3
1.248036	0.784	0.655	6.67E-89	8	SEC61G
1.417617	0.613	0.327	1.86E-86	8	AC090498.1
1.110297	0.564	0.303	5.46E-79	8	CYSTM1
1.029497	0.532	0.281	7.46E-76	8	SYNGR2
1.160244	0.663	0.463	7.14E-75	8	TSPO
1.125613	0.621	0.351	2.65E-71	8	HSPA1B
1.058598	0.586	0.353	1.52E-63	8	VMP1
1.050999	0.513	0.286	7.44E-60	8	C4orf48
1.579385	0.504	0.362	4.31E-38	8	GSTP1
1.131018	0.611	0.573	3.69E-10	8	LAMTOR4
2.729774	0.93	0.187	0	9	AHSG
2.661774	0.728	0.139	0	9	APOC2
2.655762	0.806	0.21	0	9	ANG
2.63501	0.976	0.315	0	9	FGG
2.629387	0.909	0.184	0	9	SERPINC1
2.587403	0.837	0.259	0	9	EPHX1
2.576793	0.949	0.276	0	9	FGA
2.55286	0.9	0.286	0	9	A1BG
2.516294	0.921	0.301	0	9	APOC3
2.40661	0.98	0.296	0	9	RBP4
2.387448	0.963	0.295	0	9	FGB
2.354752	0.879	0.303	0	9	C3
2.348353	0.996	0.449	0	9	AMBP
2.328134	0.796	0.17	0	9	HPX
2.321137	0.692	0.16	0	9	AGXT
2.313073	0.903	0.341	0	9	RARRES2
2.303833	0.733	0.102	0	9	HRG
2.300408	0.781	0.097	0	9	C4BPA
2.289784	0.735	0.124	0	9	APOM
2.154393	0.83	0.174	0	9	F2
2.068493	0.664	0.103	0	9	UGT2B7
2.056521	0.857	0.178	0	9	KNG1
2.056229	0.963	0.344	0	9	APOH
2.050107	0.716	0.125	0	9	UGT2B4
2.041378	0.882	0.267	0	9	GC
2.002397	0.813	0.173	0	9	ITIH3
1.977433	0.638	0.134	0	9	GPX2
1.90209	0.576	0.066	0	9	ADH1C
1.859313	0.962	0.318	0	9	VTN

1.851213	0.795	0.201	0	9	ORM2
1.823053	0.622	0.121	0	9	LEAP2
1.798138	0.812	0.216	0	9	ITIH1
1.778411	0.722	0.072	0	9	ANGPTL3
1.775163	0.711	0.12	0	9	F12
1.733956	0.614	0.088	0	9	SAA4
1.715182	0.744	0.181	0	9	C8G
1.696501	0.74	0.151	0	9	PLG
1.661823	0.636	0.117	0	9	UGT2B15
1.633897	0.617	0.127	0	9	HSD17B6
1.520939	0.737	0.166	0	9	ITIH2
1.5046	0.574	0.106	0	9	LBP
1.434971	0.709	0.145	0	9	PON1
1.401848	0.629	0.111	0	9	LRG1
1.400351	0.643	0.116	0	9	CFB
1.395114	0.558	0.046	0	9	HPR
1.384844	0.678	0.141	0	9	CPB2
1.343008	0.653	0.133	0	9	SERPINA6
1.235586	0.552	0.064	0	9	MASP2
1.224163	0.572	0.097	0	9	TFR2
1.179534	0.584	0.094	0	9	AADAC
1.149294	0.544	0.095	0	9	REEP6
1.146252	0.621	0.124	0	9	CFI
1.070328	0.513	0.082	0	9	SERPINA4
2.104218	0.999	0.61	5.67E-296	9	SERPINA1
1.452264	0.698	0.168	1.41E-287	9	CP
2.973605	0.848	0.291	2.57E-287	9	FABP1
1.282461	0.622	0.137	1.02E-285	9	ASGR2
2.486843	0.878	0.284	1.82E-284	9	ORM1
1.592034	0.812	0.24	5.67E-278	9	TMEM176A
1.973374	1	0.653	2.02E-277	9	ALB
1.702411	0.796	0.256	2.89E-272	9	CFH
1.340534	0.645	0.134	4.19E-272	9	HPD
1.561397	0.791	0.239	3.53E-271	9	C1S
1.772924	0.784	0.234	4.25E-269	9	FGL1
1.112914	0.618	0.141	7.95E-269	9	SERPIND1
1.894096	0.993	0.573	9.28E-268	9	APOA2
1.496246	0.792	0.233	4.64E-262	9	APOB
1.629674	0.84	0.272	4.00E-261	9	TMEM176B
1.78126	0.683	0.173	3.02E-254	9	APCS
1.005776	0.546	0.124	4.10E-229	9	C5
1.482772	0.844	0.322	1.95E-226	9	AGT
1.529213	0.827	0.296	3.31E-224	9	PRAP1
1.956004	0.874	0.389	1.12E-215	9	CLU
1.855168	0.532	0.129	3.37E-215	9	AKR1B10
1.372058	0.663	0.204	1.36E-212	9	C4BPB
1.625393	0.979	0.503	1.11E-208	9	APOA1
1.842957	0.572	0.144	7.07E-201	9	ALDOB
1.6494	0.796	0.323	1.71E-200	9	SERPING1
1.890521	0.971	0.569	3.18E-200	9	APOC1
1.582821	0.857	0.354	1.39E-198	9	MGST1
1.224935	0.69	0.201	1.04E-197	9	AZGP1
1.311121	0.69	0.219	6.24E-196	9	ASGR1
1.189914	0.555	0.15	1.56E-188	9	RAMP1
1.194074	0.507	0.125	4.19E-187	9	AKR1C2
1.095842	0.611	0.183	1.04E-178	9	CES2
1.03037	0.597	0.18	5.27E-177	9	SERPINF2
1.247095	0.711	0.264	4.33E-168	9	TM4SF4

1.003779	0.784	0.331	5.36E-155	9	FN1
1.019301	0.548	0.175	4.30E-154	9	HRSP12
1.030329	0.631	0.224	1.26E-152	9	PON2
1.314501	0.638	0.239	2.58E-151	9	GATM
1.224775	0.784	0.361	6.83E-150	9	CYB5A
1.011373	0.522	0.162	4.65E-149	9	DHCR24
1.082623	0.794	0.32	8.16E-147	9	CES1
2.629999	0.876	0.628	2.60E-146	9	MT-ND4L
1.171605	0.98	0.58	3.47E-145	9	APOE
1.372149	0.694	0.268	4.33E-139	9	GSTA1
1.661295	0.993	0.987	5.50E-136	9	MT-CO2
2.207117	0.956	0.833	5.45E-135	9	MT-ND5
2.199676	0.992	0.974	3.50E-129	9	MT-ND4
1.13568	0.646	0.273	8.66E-122	9	GAMT
1.215311	0.503	0.164	3.71E-121	9	MAT1A
1.771945	0.99	0.96	3.02E-115	9	MT-ATP6
1.544473	0.697	0.318	2.11E-111	9	IFI27
2.378823	0.98	0.959	1.55E-110	9	MT-ND2
1.032601	0.895	0.545	2.70E-107	9	IFITM3
1.03078	0.709	0.331	8.53E-106	9	NUPR1
1.501063	0.989	0.953	5.06E-99	9	MT-CYB
1.032011	0.785	0.452	2.75E-96	9	ATOX1
1.301767	0.542	0.22	7.11E-96	9	SCD
1.047954	0.581	0.25	1.12E-94	9	SERPINF1
1.129332	0.996	0.992	1.63E-86	9	MT-CO1
1.040085	0.729	0.442	5.30E-86	9	ETFB
2.265591	0.985	0.945	1.39E-85	9	MT-ND3
1.114925	0.621	0.415	7.32E-47	9	LY6E
5.362278	0.853	0.042	0	10	TAGLN
5.241912	0.808	0.053	0	10	ACTA2
5.046139	0.755	0.029	0	10	RGS5
4.841252	0.907	0.058	0	10	MYL9
4.672214	0.984	0.126	0	10	IGFBP7
4.044598	0.813	0.049	0	10	TPM2
3.973616	0.865	0.086	0	10	BGN
3.875271	0.68	0.013	0	10	DCN
3.779004	0.648	0.015	0	10	COL3A1
3.763015	0.971	0.29	0	10	CALD1
3.667665	0.66	0.012	0	10	COL1A2
3.649651	0.731	0.074	0	10	NDUFA4L2
3.634532	0.786	0.047	0	10	MGP
3.618907	0.558	0.062	0	10	COL1A1
3.414604	0.634	0.071	0	10	SPARCL1
3.414215	0.87	0.094	0	10	SPARC
3.412214	0.668	0.022	0	10	SOD3
3.155208	0.791	0.035	0	10	PPP1R14A
3.14688	0.793	0.02	0	10	PLAC9
2.922977	0.525	0.025	0	10	THY1
2.889601	0.525	0.053	0	10	COLEC11
2.887822	0.731	0.084	0	10	MFGE8
2.764877	0.541	0.051	0	10	IGFBP5
2.721582	0.698	0.039	0	10	COL6A2
2.572021	0.68	0.024	0	10	LHFP
2.565905	0.772	0.115	0	10	ID3
2.542084	0.627	0.065	0	10	FRZB
2.422533	0.503	0.015	0	10	CRYAB
2.392469	0.537	0.019	0	10	TPPP3
2.353255	0.634	0.049	0	10	TINAGL1

2.286368	0.591	0.088	0	10	MYLK
2.284712	0.694	0.077	0	10	MAP1B
2.279748	0.598	0.013	0	10	PDGFRB
2.256944	0.627	0.054	0	10	COL4A2
2.195075	0.618	0.01	0	10	NOTCH3
2.159742	0.639	0.117	0	10	PRKCDBP
2.102186	0.547	0.022	0	10	PGF
2.061727	0.601	0.05	0	10	4-Sep
2.02152	0.556	0.047	0	10	COL4A1
1.864664	0.667	0.106	0	10	PTRF
1.83213	0.603	0.08	0	10	NR2F2
1.727924	0.516	0.066	0	10	ISYNA1
1.677294	0.528	0.025	0	10	TGFB1I1
1.785891	0.653	0.126	7.64E-302	10	CRIP2
2.83468	0.824	0.276	6.78E-300	10	TPM1
2.071172	0.72	0.18	3.01E-289	10	CNN3
4.012197	0.895	0.366	4.85E-281	10	TIMP1
1.702347	0.712	0.166	3.14E-273	10	GSN
1.84236	0.634	0.133	4.32E-269	10	CAV1
2.198224	0.981	0.559	1.70E-263	10	LGALS1
1.872849	0.61	0.123	1.17E-260	10	MEF2C
2.493885	0.717	0.206	4.08E-255	10	GPX3
1.224937	0.553	0.098	5.44E-247	10	TCF4
1.129651	0.642	0.128	2.28E-242	10	COX7A1
1.668868	0.509	0.093	1.90E-239	10	TIMP3
2.010195	0.603	0.14	2.67E-239	10	SERPINH1
2.022445	0.637	0.156	1.69E-235	10	COL18A1
3.454985	0.63	0.162	1.09E-230	10	ADIRF
2.180076	0.554	0.128	1.97E-226	10	CSRP2
1.753514	0.979	0.544	7.54E-218	10	IFITM3
2.03317	0.803	0.33	9.78E-218	10	SELM
2.096967	0.509	0.112	4.21E-205	10	PTP4A3
1.798293	0.644	0.191	2.44E-197	10	TSC22D1
2.791229	0.857	0.484	5.50E-190	10	DSTN
2.068001	0.67	0.238	1.13E-185	10	OAZ2
1.800078	0.914	0.53	2.98E-185	10	VIM
1.562886	0.943	0.771	1.10E-174	10	CALM2
1.573135	0.61	0.19	1.68E-163	10	WBP5
1.65795	0.579	0.186	9.82E-161	10	HCFC1R1
1.458792	0.921	0.572	5.65E-157	10	MT2A
1.676076	0.832	0.485	1.34E-141	10	ITGB1
1.357987	0.506	0.152	1.47E-139	10	PKIG
1.703101	0.513	0.145	9.14E-139	10	RBP1
1.103591	0.934	0.53	2.11E-137	10	CST3
1.57359	0.615	0.222	1.16E-135	10	FLNA
1.53544	0.677	0.261	4.12E-134	10	TUBA1A
1.494152	0.765	0.393	1.52E-132	10	SEPW1
1.462677	0.713	0.325	2.50E-131	10	SH3BGRL
1.518278	0.604	0.225	3.70E-128	10	IGFBP4
1.638701	0.917	0.65	2.89E-126	10	HSPB1
1.470292	0.656	0.278	5.22E-126	10	CD151
1.343617	0.955	0.886	3.92E-124	10	MYL6
1.483645	0.554	0.187	7.44E-121	10	CD9
1.287737	0.675	0.28	4.24E-120	10	NGFRAP1
1.258913	0.729	0.358	2.43E-114	10	CD59
1.346935	0.568	0.22	6.40E-110	10	LGALS3BP
1.468658	0.516	0.183	7.20E-107	10	PHLDA1
1.187143	0.898	0.765	6.00E-103	10	ITM2B

1.352667	0.798	0.505	4.83E-101	10	MGST3
1.26809	0.8	0.51	2.60E-99	10	7-Sep
1.884953	0.522	0.206	2.14E-97	10	EGR1
1.397878	0.769	0.483	7.85E-97	10	LAPTM4A
1.429405	0.724	0.411	1.19E-96	10	LMNA
1.235639	0.725	0.459	3.44E-89	10	EID1
1.292534	0.634	0.329	1.96E-82	10	TPM4
1.152973	0.661	0.331	2.26E-78	10	SERPING1
1.109939	0.523	0.229	6.68E-78	10	PDLIM1
2.030499	0.772	0.565	1.05E-74	10	FOS
1.086423	0.788	0.579	5.95E-74	10	MORF4L1
1.033829	0.566	0.265	1.54E-67	10	NBEAL1
1.02791	0.786	0.6	9.48E-64	10	CIRBP
2.414092	0.544	0.276	3.08E-63	10	MTRNR2L8
2.005113	0.713	0.519	1.83E-58	10	JUN
1.026051	0.525	0.269	2.83E-57	10	TCEAL4
1.678483	0.591	0.324	3.53E-57	10	CRIP1
1.150266	0.632	0.34	7.12E-57	10	FN1
2.234192	0.591	0.357	2.38E-49	10	MTRNR2L12
1.381603	0.539	0.355	3.76E-32	10	GADD45B
1.189607	0.544	0.357	3.56E-28	10	HSPA1B
1.461261	0.649	0.504	1.18E-26	10	HSPA1A
1.168467	0.774	0.667	9.74E-25	10	JUNB
1.000938	0.608	0.459	7.67E-22	10	IER2
3.877591	0.925	0.155	0	11	C1QB
3.703601	0.942	0.164	0	11	C1QA
3.269895	0.896	0.102	0	11	C1QC
3.093936	0.964	0.236	0	11	TYROBP
3.053107	0.648	0.035	0	11	CXCL9
3.049619	0.971	0.154	0	11	AIF1
2.944184	0.959	0.173	0	11	FCER1G
2.623029	0.829	0.132	0	11	CD14
2.555228	0.846	0.097	0	11	FAM26F
2.505819	0.896	0.148	0	11	CD68
2.410155	0.901	0.159	0	11	HLA-DQA1
2.328891	0.892	0.171	0	11	CAPG
2.113931	0.786	0.077	0	11	FCGR3A
2.045595	0.846	0.085	0	11	MS4A6A
2.018387	0.889	0.079	0	11	SPI1
1.990786	0.814	0.06	0	11	IGSF6
1.968699	0.863	0.112	0	11	HLA-DMB
1.938246	0.877	0.13	0	11	C1orf162
1.927937	0.708	0.032	0	11	TREM2
1.842959	0.904	0.149	0	11	LST1
1.815907	0.798	0.066	0	11	MS4A7
1.804359	0.781	0.056	0	11	FCGR2A
1.757072	0.682	0.088	0	11	HMOX1
1.724748	0.711	0.036	0	11	VSIG4
1.693626	0.684	0.08	0	11	GPNMB
1.650255	0.795	0.103	0	11	PLAUR
1.591097	0.742	0.04	0	11	LILRB4
1.584754	0.6	0.057	0	11	HBEGF
1.554902	0.802	0.084	0	11	TNFSF13B
1.543564	0.614	0.048	0	11	IL4I1
1.470492	0.781	0.086	0	11	FGL2
1.466337	0.759	0.065	0	11	RNASE6
1.457373	0.677	0.093	0	11	EPB41L2
1.452858	0.781	0.113	0	11	LGALS9

1.366208	0.733	0.114	0	11	TGFBI
1.309452	0.716	0.055	0	11	CYBB
1.27617	0.631	0.045	0	11	MS4A4A
1.266798	0.629	0.059	0	11	IFI30
1.25447	0.672	0.072	0	11	C1orf54
1.235221	0.627	0.052	0	11	CD163
1.226811	0.641	0.04	0	11	MSR1
1.225182	0.557	0.035	0	11	VMO1
1.219256	0.68	0.044	0	11	CSF1R
1.161128	0.66	0.039	0	11	CD86
1.159613	0.665	0.062	0	11	RAB31
1.141846	0.651	0.055	0	11	ADAP2
1.140064	0.694	0.085	0	11	KCTD12
1.126196	0.627	0.078	0	11	OLFML3
1.112316	0.586	0.051	0	11	BASP1
1.095408	0.643	0.065	0	11	LAIR1
1.078642	0.646	0.051	0	11	LY86
1.031922	0.622	0.062	0	11	CD300A
1.011593	0.52	0.026	0	11	KCNMA1
1.004458	0.542	0.041	0	11	LHFPL2
1.204538	0.631	0.09	9.49E-301	11	GM2A
1.276485	0.711	0.111	9.02E-289	11	CD4
1.829653	0.913	0.218	1.01E-278	11	HLA-DMA
1.262811	0.745	0.126	4.41E-275	11	SGK1
2.702607	0.981	0.349	4.56E-274	11	HLA-DPA1
1.632822	0.759	0.149	1.02E-265	11	LGMN
1.121269	0.74	0.134	5.54E-262	11	CXCL16
1.364049	0.742	0.14	1.04E-261	11	SLC16A3
2.62349	0.995	0.377	2.20E-261	11	HLA-DRA
2.595766	0.983	0.383	3.70E-259	11	HLA-DPB1
1.742735	0.841	0.209	2.97E-254	11	CTSH
2.168426	0.906	0.287	1.29E-246	11	CTSZ
1.222636	0.733	0.147	6.26E-245	11	CREG1
1.082498	0.675	0.109	8.93E-242	11	PLEK
2.74892	0.976	0.377	1.57E-241	11	HLA-DRB1
1.700808	0.908	0.236	3.81E-235	11	LYZ
1.197654	0.651	0.117	1.84E-234	11	ATF5
1.120202	0.667	0.116	3.87E-232	11	ABI3
1.36131	0.88	0.2	4.27E-230	11	HLA-DQB1
1.004171	0.578	0.089	1.25E-229	11	MAFB
2.704983	0.925	0.363	4.80E-229	11	CTSB
2.965006	0.995	0.625	6.70E-225	11	CD74
2.173552	0.937	0.364	1.24E-224	11	TYMP
2.173427	0.971	0.47	4.21E-218	11	NPC2
2.481163	0.99	0.588	4.92E-215	11	SAT1
1.143879	0.667	0.13	7.69E-215	11	DAB2
1.425417	0.665	0.13	1.23E-207	11	C15orf48
2.161713	0.99	0.58	2.72E-204	11	GPX1
2.882586	0.959	0.525	1.76E-203	11	PSAP
1.841603	0.961	0.41	7.81E-202	11	LAPTM5
1.585814	0.894	0.287	5.06E-201	11	CTSS
1.121237	0.687	0.146	2.74E-200	11	PLEKHO1
1.486415	0.899	0.27	1.36E-199	11	ITGB2
2.099782	0.988	0.583	1.00E-197	11	S100A11
2.000517	0.986	0.533	3.33E-188	11	CST3
3.102886	0.887	0.288	6.53E-188	11	SPP1
1.030335	0.595	0.125	1.25E-179	11	CAMK1
1.064317	0.769	0.184	1.15E-178	11	UCP2

2.053643	0.708	0.17	9.04E-178	11	RNASE1
1.100678	0.67	0.144	1.47E-177	11	CCL3
2.318988	1	0.976	2.93E-171	11	FTL
1.045898	0.764	0.213	1.05E-170	11	PDXK
1.374691	0.896	0.274	4.25E-170	11	FABP5
1.131216	0.812	0.219	4.97E-169	11	RGS10
1.175927	0.716	0.188	1.37E-168	11	LIPA
1.597717	0.923	0.466	5.45E-162	11	ATP6V1F
1.625371	0.892	0.379	8.67E-162	11	GRN
1.690212	0.911	0.385	4.90E-161	11	RNASET2
1.095836	0.658	0.153	9.85E-161	11	GBP1
1.059152	0.728	0.201	6.10E-155	11	PPT1
1.262206	0.754	0.234	3.74E-153	11	PLD3
1.472785	0.947	0.507	3.96E-153	11	VAMP8
1.097096	0.798	0.239	3.35E-152	11	PYCARD
1.855173	0.976	0.563	9.66E-151	11	LGALS1
1.261674	0.788	0.251	1.42E-150	11	MARCKS
1.892287	0.933	0.526	4.83E-148	11	CTSD
1.545979	0.742	0.239	7.46E-148	11	CTSL
1.36966	0.86	0.339	8.60E-147	11	FCGRT
1.010104	0.757	0.23	1.90E-146	11	TPP1
1.72079	0.978	0.578	3.54E-145	11	TUBA1B
1.546892	0.892	0.375	1.56E-142	11	SOD2
1.75058	0.969	0.583	1.25E-141	11	CSTB
1.124046	0.8	0.268	1.73E-141	11	ASAH1
1.636253	1	0.984	2.52E-140	11	FTH1
1.320963	0.848	0.293	2.04E-137	11	LGALS3
1.23837	0.841	0.326	2.14E-135	11	YWHAH
1.340784	0.892	0.348	9.85E-133	11	COTL1
1.152731	0.986	0.834	2.23E-131	11	OAZ1
1.235955	0.925	0.438	2.36E-126	11	PKM
1.179327	0.86	0.325	8.17E-122	11	RGS1
1.125523	0.617	0.168	9.90E-122	11	ACP5
1.469928	0.947	0.617	1.05E-120	11	CYBA
1.001436	0.571	0.153	2.45E-120	11	TFRC
1.019257	0.81	0.296	6.34E-118	11	STAT1
1.078446	0.795	0.28	8.06E-116	11	RGS2
1.01495	1	0.928	2.36E-108	11	GAPDH
1.012036	0.998	0.952	2.91E-107	11	TMSB10
1.213099	0.988	0.7	2.69E-106	11	CD63
1.063595	0.976	0.78	3.55E-102	11	YBX1
1.011612	0.836	0.367	7.86E-97	11	LITAF
1.007249	0.802	0.336	5.24E-96	11	CD81
1.101584	0.788	0.326	1.74E-95	11	CEBPD
1.157452	0.928	0.577	7.08E-91	11	PSME2
1.004184	1	0.95	1.01E-85	11	TMSB4X
1.002826	0.947	0.559	2.69E-79	11	SRGN
1.009603	0.952	0.703	6.58E-79	11	ALDOA
1.368739	0.945	0.588	1.58E-77	11	APOE
1.013806	0.928	0.578	8.94E-51	11	HMGN2
1.989931	0.971	0.465	2.95E-188	12	JUND
2.169117	0.647	0.177	3.32E-129	12	GZMK
1.439192	0.798	0.28	7.36E-127	12	TNFAIP3
1.449598	0.853	0.359	1.54E-125	12	RSRP1
1.20099	0.895	0.305	2.06E-124	12	CD3E
1.482928	0.982	0.82	6.55E-123	12	HSP90AA1
1.26853	0.927	0.386	1.97E-121	12	PTPRC
1.281591	0.887	0.317	1.24E-118	12	CD2

1.111134	0.623	0.162	1.73E-117	12	RUNX3
1.302357	0.733	0.245	2.20E-110	12	ARL4C
1.443486	0.927	0.56	1.28E-109	12	DNAJA1
1.768717	0.751	0.263	1.10E-107	12	GZMA
1.45122	0.84	0.338	3.35E-106	12	DUSP2
1.064619	0.704	0.207	4.67E-106	12	CD3G
1.121962	0.66	0.204	2.09E-103	12	ETS1
1.185614	0.832	0.322	4.59E-101	12	CD48
1.392766	0.704	0.241	3.49E-93	12	CST7
1.037011	0.984	0.889	2.86E-91	12	HLA-B
1.100585	0.877	0.36	1.88E-90	12	CD3D
1.236433	0.966	0.748	2.96E-90	12	BTG1
1.097071	0.955	0.745	3.27E-83	12	HSPA8
1.001186	0.798	0.344	1.87E-77	12	EVL
1.086419	0.757	0.338	2.41E-77	12	CD81
1.672539	0.581	0.206	2.61E-77	12	IL7R
1.001691	0.636	0.228	9.39E-75	12	CYTIP
1.350262	0.665	0.277	4.58E-72	12	ZNF331
1.21683	0.89	0.557	1.60E-69	12	ZFP36L2
1.055303	0.599	0.23	2.95E-67	12	SYNE2
1.023528	0.872	0.534	4.44E-66	12	SRSF7
1.099181	0.51	0.186	1.30E-65	12	ZNF292
1.268702	0.738	0.362	2.14E-61	12	ANXA1
1.019119	0.675	0.297	4.85E-56	12	RGCC
1.157714	0.552	0.221	3.32E-52	12	TRBC1
1.051152	0.683	0.34	6.60E-51	12	TXNIP
1.344117	0.814	0.555	1.32E-47	12	PGK1
1.109024	0.882	0.628	1.33E-47	12	LDHA
1.295837	0.584	0.309	1.02E-45	12	CORO1B
1.293102	0.885	0.644	5.29E-42	12	ENO1
8.514217	0.932	0.158	0	13	IGHG3
7.317563	0.846	0.016	0	13	IGHGP
7.158673	0.908	0.061	0	13	IGHG4
6.647792	0.929	0.15	0	13	IGHG1
6.302515	0.917	0.059	0	13	JCHAIN
5.598794	0.862	0.044	0	13	IGHG2
3.872944	0.985	0.056	0	13	MZB1
2.641854	0.865	0.03	0	13	DERL3
1.709234	0.8	0.035	0	13	CD79A
1.625447	0.794	0.108	0	13	PIM2
1.458787	0.637	0.007	0	13	TNFRSF17
1.089357	0.575	0.007	0	13	FCRL5
2.118117	0.96	0.255	1.56E-243	13	FKBP11
7.092782	0.985	0.4	1.71E-214	13	IGKC
1.597352	0.852	0.192	3.80E-206	13	ITM2C
1.399297	0.689	0.125	9.01E-203	13	ANKRD28
2.943472	0.997	0.702	9.34E-203	13	SSR4
6.360766	0.923	0.261	1.13E-197	13	IGLC2
2.383593	0.982	0.474	1.88E-179	13	XBP1
2.301627	0.942	0.412	1.45E-167	13	HERPUD1
1.556064	0.862	0.282	6.25E-144	13	DNAJB9
5.466741	0.763	0.191	1.09E-139	13	IGLC3
1.094936	0.56	0.107	1.71E-133	13	PRDM1
1.251954	0.898	0.338	3.84E-124	13	PRDX4
1.169977	0.825	0.29	1.32E-113	13	SPCS3
1.045383	0.766	0.26	3.63E-109	13	ERLEC1
1.381819	0.898	0.393	4.08E-107	13	SEC11C
1.131727	0.948	0.551	2.86E-86	13	SPCS2

1.128914	0.523	0.14	4.30E-83	13	ANKRD37
6.052459	0.56	0.157	5.20E-81	13	IGHA1
1.392076	0.935	0.518	1.54E-74	13	JUN
1.101832	0.766	0.356	7.77E-63	13	HSPA1B
4.396059	0.861	0.192	1.23E-165	14	IGLC3
3.48756	0.996	0.403	2.41E-127	14	IGKC
4.129003	0.892	0.264	4.51E-127	14	IGLC2
1.537614	0.656	0.136	1.20E-125	14	CD27
1.081374	0.996	0.369	1.10E-124	14	SNHG25
1.731874	0.842	0.31	4.79E-104	14	ISG20
1.535417	0.865	0.33	1.01E-78	14	RGS1
3.090915	0.556	0.159	1.27E-70	14	IGHG1
1.26339	0.981	0.889	3.97E-69	14	HLA-B
1.041771	0.768	0.326	2.26E-48	14	LSP1
1.103608	0.981	0.894	9.26E-41	14	HLA-A
1.046774	0.533	0.193	1.37E-40	14	UCP2
1.11565	0.819	0.472	2.88E-37	14	PSMB9
1.558527	0.571	0.268	1.95E-33	14	CD7
1.214521	0.695	0.366	1.67E-32	14	CD3D
1.101518	0.668	0.325	4.73E-31	14	CD2
1.213024	0.668	0.357	9.68E-30	14	COTL1
1.026316	0.687	0.35	7.96E-28	14	TRAC
1.336759	0.598	0.31	4.50E-24	14	TRBC2
1.321806	0.51	0.254	5.77E-21	14	NKG7
1.178705	0.83	0.705	3.83E-15	14	SSR4
2.540142	0.708	0.063	0	15	KIAA0101
2.113089	0.625	0.04	0	15	MKI67
2.462689	0.577	0.057	6.34E-254	15	TOP2A
1.879959	0.577	0.058	1.47E-252	15	NUSAP1
2.094896	0.625	0.072	8.93E-249	15	TYMS
2.314874	0.593	0.062	3.05E-248	15	UBE2C
1.983414	0.518	0.06	1.08E-190	15	CENPF
1.65074	0.542	0.069	5.11E-182	15	BIRC5
3.074203	0.921	0.28	6.77E-179	15	STMN1
3.358959	0.917	0.301	1.96E-174	15	HMGB2
1.31052	0.518	0.095	2.00E-118	15	SMC2
1.809054	0.64	0.144	2.00E-115	15	PTTG1
1.971904	0.98	0.768	8.80E-110	15	HMGB1
1.660123	0.64	0.164	7.01E-105	15	SMC4
2.289678	0.925	0.582	8.71E-102	15	HMGN2
2.05624	0.901	0.596	6.69E-89	15	H2AFZ
2.345996	0.862	0.469	8.11E-84	15	TUBB
2.243893	0.905	0.583	2.20E-83	15	TUBA1B
1.61175	0.996	0.964	1.39E-82	15	ACTB
1.626485	0.569	0.157	1.19E-81	15	PCNA
1.655089	0.98	0.875	2.11E-80	15	PFN1
1.267313	0.526	0.133	2.51E-79	15	DNAJC9
1.35612	0.992	0.956	2.72E-79	15	PTMA
1.739581	0.877	0.436	3.07E-79	15	CORO1A
2.94755	0.767	0.384	1.29E-72	15	HIST1H4C
1.719988	0.676	0.251	5.53E-69	15	ANP32E
1.225618	0.51	0.14	7.00E-68	15	MCM7
1.849959	0.779	0.355	1.11E-66	15	COTL1
1.251978	0.96	0.861	4.37E-64	15	CFL1
1.477401	0.802	0.432	9.16E-64	15	ANP32B
1.524158	0.806	0.468	5.57E-62	15	H2AFV
1.810792	0.7	0.327	4.49E-61	15	DUT
1.388432	0.561	0.19	1.55E-59	15	TMPO

1.493794	0.794	0.435	1.69E-59	15	CALM3
1.610251	0.755	0.413	4.15E-59	15	DEK
1.178981	0.514	0.154	5.37E-58	15	H2AFX
1.212339	0.743	0.329	2.53E-53	15	RAC2
1.14355	0.538	0.178	4.19E-53	15	NUDT1
1.555189	0.656	0.252	2.15E-52	15	NKG7
1.346474	0.814	0.575	8.23E-51	15	RAN
1.75899	0.66	0.268	2.90E-50	15	GZMA
1.18412	0.549	0.197	2.04E-49	15	DNMT1
1.087684	0.557	0.187	1.04E-48	15	APOBEC3G
1.318337	0.609	0.243	1.10E-48	15	CKS2
1.162619	0.565	0.214	1.27E-48	15	DDX39A
1.421425	0.735	0.327	1.35E-48	15	CRIP1
1.302939	0.609	0.223	1.31E-46	15	ITM2A
1.179063	0.881	0.603	4.32E-46	15	HMGN1
1.327889	0.676	0.342	1.67E-45	15	IDH2
1.229298	0.711	0.327	5.70E-45	15	LSP1
1.17037	0.866	0.703	5.29E-44	15	ARPC2
1.320294	0.767	0.472	4.68E-42	15	PSMB9
1.030906	1	0.951	6.04E-42	15	TMSB4X
1.198857	0.605	0.255	8.49E-42	15	C12orf75
1.323396	0.759	0.444	1.89E-41	15	CBX3
1.207123	0.613	0.274	5.38E-41	15	CKS1B
1.112813	0.747	0.335	1.15E-40	15	CCL5
1.296506	0.719	0.43	1.83E-40	15	RANBP1
1.15964	0.905	0.818	5.43E-38	15	ACTG1
1.125923	0.628	0.305	5.29E-36	15	BUB3
1.027015	0.775	0.423	6.21E-36	15	GMFG
1.097088	0.834	0.537	2.15E-35	15	ARHGDIB
1.013199	0.7	0.356	4.80E-35	15	LDHB
1.196425	0.791	0.582	7.94E-34	15	PSME2
1.002839	0.514	0.221	1.13E-32	15	SLBP
1.041709	0.751	0.502	2.57E-32	15	PPP1CA
1.039408	0.589	0.301	2.27E-31	15	RPA3
1.00831	0.806	0.579	4.93E-31	15	SRSF3
1.001507	0.711	0.434	9.52E-31	15	SNRPD1
1.101193	0.68	0.391	3.25E-30	15	PSMB8
1.08802	0.696	0.424	3.50E-30	15	SIVA1
1.051881	0.585	0.286	3.54E-30	15	NASP
1.043618	0.621	0.337	1.87E-29	15	CARHSP1
1.023671	0.842	0.74	3.32E-29	15	SUB1
1.055824	0.735	0.526	2.86E-28	15	SNRPB
1.032963	0.609	0.344	1.91E-24	15	H2AFY
1.013944	0.7	0.435	1.28E-23	15	CKLF
1.000267	0.925	0.822	3.57E-20	15	HSP90AA1
1.012403	0.644	0.402	8.83E-20	15	ACTR3
1.128305	0.644	0.438	8.44E-17	15	TUBB4B
1.563151	0.581	0.412	1.25E-09	15	ARL6IP1
