# Supplementary material for: The Role of Ferroptosis and Cuproptosis in Curcumin against Hepatocellular Carcinoma
Source: Molecules. 2023 Feb 8;28(4):1623. doi: 10.3390/molecules28041623 (PMC9964324; doi:10.3390/molecules28041623)
Supplement: Supplementary file 1 [file molecules-28-01623-s001.zip › Figure S1.pdf]

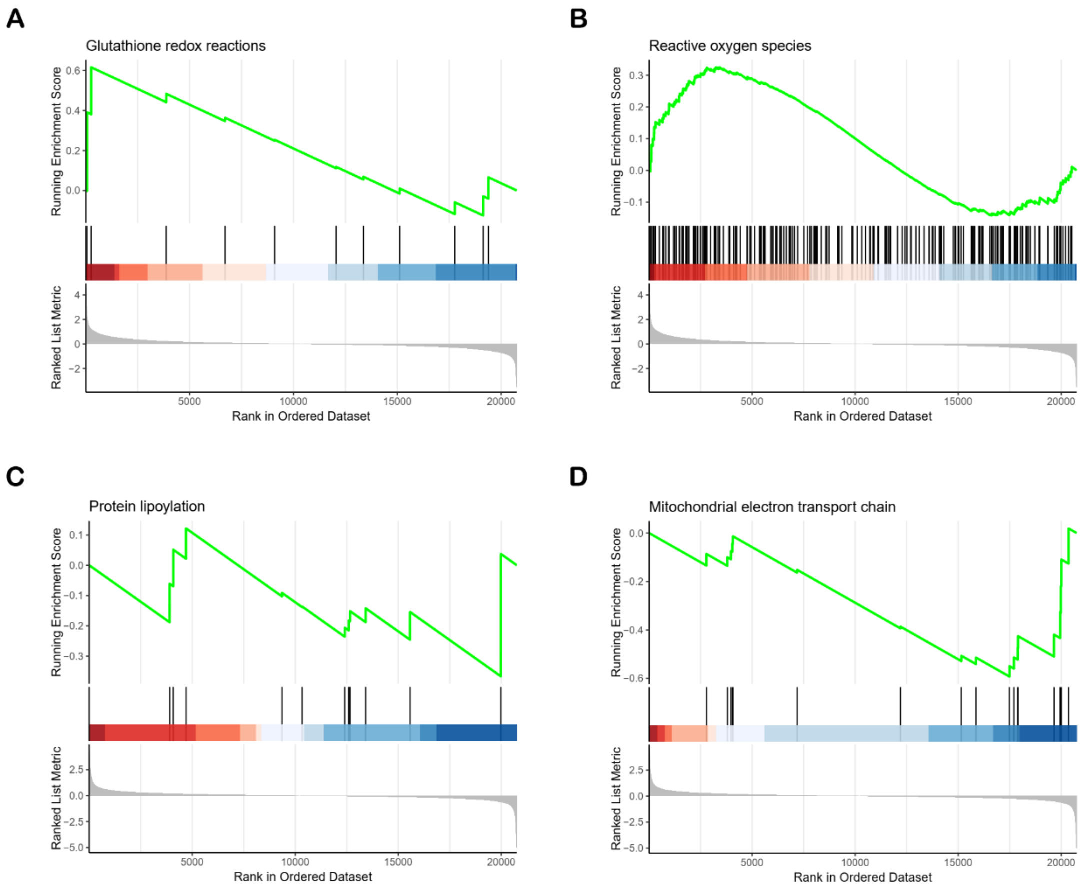

Figure S1. Gene set enrichment analysis (GSEA) of characterized events in ferroptosis and cuproptosis. (A) GSEA of glutathione redox reactions showed that glutathione was over depleted in PLC cells after curcumin treatment. (B) GSEA of reactive oxygen species showed that the level of reactive oxygen species was elevated in PLC cells after curcumin treatment. (C) GSEA of protein lipoylation showed that the level of lipoylation was inhibited in KMCH cells after curcumin treatment. (D) GSEA of mitochondrial electron transport chain showed that the pathway was suppressed in KMCH cells after curcumin treatment.
